# Global comparative analysis of ESTs from the southern cattle tick, *Rhipicephalus (Boophilus) microplus*

**DOI:** 10.1186/1471-2164-8-368

**Published:** 2007-10-12

**Authors:** Minghua Wang, Felix D Guerrero, Geo Pertea, Vishvanath M Nene

**Affiliations:** 1 Lorus Therapeutics Inc; 2 Meridian Road, Toronto, ON M9W 4Z7, Canada; 2 USDA-ARS, Knipling Bushland U.S. Livestock Insect Research Laboratory; 2700 Fredericksburg Rd., Kerrville, TX 78028, USA; 3 The J. Craig Venter Institute, 9712 Medical Center Drive, Rockville, Maryland 20850, USA

## Abstract

**Background:**

The southern cattle tick, *Rhipicephalus (Boophilus) microplus*, is an economically important parasite of cattle and can transmit several pathogenic microorganisms to its cattle host during the feeding process. Understanding the biology and genomics of *R. microplus *is critical to developing novel methods for controlling these ticks.

**Results:**

We present a global comparative genomic analysis of a gene index of *R. microplus *comprised of 13,643 unique transcripts assembled from 42,512 expressed sequence tags (ESTs), a significant fraction of the complement of *R. microplus *genes. The source material for these ESTs consisted of polyA RNA from various tissues, lifestages, and strains of *R. microplus*, including larvae exposed to heat, cold, host odor, and acaricide. Functional annotation using RPS-Blast analysis identified conserved protein domains in the conceptually translated gene index and assigned GO terms to those database transcripts which had informative BlastX hits. Blast Score Ratio and SimiTri analysis compared the conceptual transcriptome of the *R. microplus *database to other eukaryotic proteomes and EST databases, including those from 3 ticks. The most abundant protein domains in BmiGI were also analyzed by SimiTri methodology.

**Conclusion:**

These results indicate that a large fraction of BmiGI entries have no homologs in other sequenced genomes. Analysis with the PartiGene annotation pipeline showed 64% of the members of BmiGI could not be assigned GO annotation, thus minimal information is available about a significant fraction of the tick genome. This highlights the important insights in tick biology which are likely to result from a tick genome sequencing project. Global comparative analysis identified some tick genes with unexpected phylogenetic relationships which detailed analysis attributed to gene losses in some members of the animal kingdom. Some tick genes were identified which had close orthologues to mammalian genes. Members of this group would likely be poor choices as targets for development of novel tick control technology.

## Background

*Rhipicephalus (Boophilus) microplus*, the tropical or southern cattle tick, is one of the most economically important tick vectors of pathogens that affect the global cattle population [1]. The tick transmits protozoan (*Babesia bovis *and *Babesia bigemina*) and prokaryotic (*Anaplasma marginale*) organisms that cause babesiosis and anaplasmosis, which can result in severe agricultural losses in milk and beef production and restriction in traffic of livestock. The impact of *R. microplus *upon the US cattle industry was such that the US Department of Agriculture (USDA) led a campaign in the mid-20th century which eradicated the tick from the US [2]. The tick remains prevalent in Mexico and, since over a million cattle are imported annually into the US from Mexico, an extensive USDA quarantine program is in place to keep *Boophilus *ticks from reestablishing in the US [3].

Acaricides play a critical role in maintaining the success of the USDA quarantine program and in controlling tick infestations in Mexico and other parts of the world. However, reports of acaricide resistant *R. microplus *populations in Mexico [4,5] and *R. microplus *outbreaks in the US [6] highlight the need for development of novel tick control methodologies. Understanding the genome and the gene expression profile of the tick should facilitate the development of these control technologies. Several reports have described projects centered on the acquisition and analysis of tick expressed sequence tags (ESTs). Most of the reports focused on the genes transcribed in the salivary glands of ticks such as *Rhipicephalus appendiculatus *[7], *Amblyomma variegatum *[8] and *Ixodes scapularis *[9]. Additionally, the isolation of 1,344 ESTs from ovaries, salivary glands and hemocytes of *R. microplus *has been reported, however, the sequences have not been submitted to Genbank [10]. Genes expressed in salivary glands and ovaries are attractive targets for study because these tissues are involved in critical tick-host-pathogen interactions. In a more general approach, we have developed a *R. microplus *EST database, BmiGI [11], derived from various tissues, lifestages and tick strains, to facilitate research using molecular biological and genomic approaches to design novel tick control technologies. It is hoped the analysis of the database will lead to discovery of genes which can overcome tick control problems due to acaricide resistance and identify gene-based vulnerabilities in the processes involved in pathogen infection and transmission. In BmiGI Version 1, 53 putative acaricide resistance-associated sequences were identified. In the present study, we have assembled an updated gene index [12] which contains more than double the number of ESTs of Version 1. We present the Gene Ontology (GO) annotation analysis and RPS-Blast identification of conserved protein domains from BmiGI Version 2. Using the comparative genomics analytical tools Blast Score Ratio [13] and SimiTri [14] which provide visual outputs to allow global comparisons between genomes, we compared the proteome resulting from the conceptual translation of the *R. microplus *EST database with the proteomes from *Homo sapiens*, *Anopheles gambiae*, *Drosophila melanogaster*, *Caenorhabditis elegans*, and *Saccharomyces cerevisiae*. We also performed more detailed SimiTri comparisons using several of the most abundant protein domains in the proteome of *R. microplus*.

## Results and discussion

### BmiGI statistics and GO annotation

In the first version of BmiGI, ESTs were clustered and assembled into tentative consensus (TC) sequences using TIGR's autoannotation pipeline tools, and non-clustered, non-overlapping sequences defined as singleton sequences. A total of 20,417 ESTs were analyzed and the assembly yielded 8,270 unique members, including 5,760 TCs and 2,510 singleton ESTs [11]. In the second version of BmiGI, the total number of new ESTs sequenced was 22,095. These new sequences were combined with the ESTs in the BmiGI Version 1 for clustering to generate BmiGI Version 2, resulting in 9,403 TCs and 4,240 singletons.

The number of novel sequences obtained significantly decreased as EST sequencing proceeded. The first 20,417 ESTs resulted in 8,270 unique members of BmiGI, a return rate of 41%. The second set, comprised of 22,095 ESTs, resulted in an additional 5,373 new members of BmiGI, a return rate of 24%. By the final stages of the second round of EST sequencing, a return rate of approximately 5% was being observed and further EST sequencing of this pooled normalized cDNA library no longer seemed an efficient use of resources. Future EST sequencing would likely be more efficient if performed on libraries synthesized from targeted tissues of specific interest, such as synganglia, ovaries or salivary glands. Sequencing of several targeted libraries is underway.

The latest release of the annotation for the *D. melanogaster *genome sequence [15] notes 19,783 protein-coding transcripts. The latest genome assembly for the *A. gambiae *[16] has noted 14,089 gene transcripts. Assuming *R. microplus *has a similar number of transcripts as these two arthropods, the BmiGI set of 13,643 unique transcripts represents a significant fraction of the likely set of protein-coding transcripts in *R. microplus*. However, it is likely that BmiGI contains ESTs which are derived from non-coding RNAs, as EST databases have been shown to contain non-coding RNAs [17]. Additionally, during use of BmiGI following annotation by BLAST analysis, it was noticed that some sequences had very high amino acid identity to bovine sequences. These likely resulted from bovine blood remaining in the gut of the adult ticks, one of the lifestages sampled and included in the pooled RNA used to synthesize the cDNA library. Additionally, some sequences appeared to be of protozoan origin and might have originated from commensual organisms within the tick or from a sample of *Babesia bovis*-infected larvae included during the library synthesis. The autoannotation pipeline used for assembling the gene index was not readily adaptable to remove bovine or protozoan sequences and this should be considered when using BmiGI. However, in our experience, these do not form an appreciable fraction of the BmiGI entries and should be easily identifiable by their high nucleotide identity to bovine or protozoan sequences in GenBank Blast search results.

Functional annotation of BmiGI to assign GO terms and assist in identification of gene function was performed with two different methods, the TIGR autoannotation pipeline and the PartiGene open source software package. In the BmiGI Version 2 web-hosted annotation, the GO analysis is presented using the TIGR autoannotation pipeline in which the cutoff for annotation was based on a search of a non-redundant protein database with E < 1 × 10^-27^. The top protein hit with at least 75% similarity and 50% coverage was taken and the GO terms assigned to this protein transferred to the TC query. The stringent cutoff is utilized to minimize annotations of false positives from the Blast analysis and singletons are not assigned GO terms. As noted in Table 1, the TIGR analysis assigned 1369, 1321, and 1253 TCs to the molecular function, biological process, and cellular component ontologies, respectively. Catalytic activity and binding are the top two assigned GO terms in the molecular function category. Physiological process and cellular process are the top two assigned GO terms for the biological process category. Finally, intracellular and cell are the top two assigned GO terms for the cellular component category.

**Table 1:** Gene ontology assignment using different cutoffs

Category	TIGR E < 1 × 10^-27^	PartiGene E < 1 × 10^-25^		PartiGene E < 1 × 10^-8^	
*Total annotated*	TCs -	TCs 2615	singletons 730	TCs 3608	singletons 1096
*Molecular function*	1369	2297	644	3147	971

Catalytic activity	745	1190	332	1503	470
Binding	702	1191	329	1649	517
Molecular function unknown	252	49	7	72	12
Transport activity	216	160	0	216	74
Structural molecule activity	174	117	0	154	28
Transcription regulator activity	111	27	10	45	14
Signal transducer activity	89	79	25	130	37
Enzyme regulator activity	72	65	14	97	28
Antioxidant activity	21	4	0	6	0
Motor Activity	14	8	0	11	0

*Biological process*	1321	1804	508	2373	736

Physiological process	1212	1044	274	1326	394
Cellular process	645	17	4	24	10
Nucleic acid metabolism	438	412	130	577	195
Development	409	90	31	128	44
Regulation of biological process	357	7	0	11	1
Biological process unknown	220	11	4	23	6
Behavior	77	2	0	5	1

*Cellular component*	1253	1231	356	1700	539

Intracellular	1204	938	245	1218	365
Cell	1164	388	124	566	196
Cellular component unknown	291	9	5	20	7
Extracellular	90	66	9	107	15
Unlocalized	15	9	5	9	6

We wished to attempt to predict gene function for TCs which were designated as unknowns and not assigned GO terms by the TIGR pipeline and to include GO annotation analysis for singletons when possible. Thus, we tried the software annot8r_blast2go in the PartiGene pipeline [18], using Blast E-values of 1 × 10^-8 ^and 1 × 10^-25 ^(Table 1). When the E-value is set at 1 × 10^-25^, 2,615 TCs (28% of the total TCs) and 730 singletons (17% of the total singletons) can be assigned a GO annotation. When the E-value is set at 1 × 10^-8^, 3,608 TCs (38%) and 1096 singletons (26%) can be assigned one or more GO terms. Thus, 66% of the members of BmiGI could not be assigned GO annotation, even using a relatively liberal E-value in the Blast. Singletons were annotated at a lower ratio of the total possible than TCs, most likely due to the singletons generally containing shorter sequence lengths compared to TCs. It is possible that some singletons represent transcripts from low copy number genes which might be unique to ticks or from genes with low sequence identity to those from organisms better represented in gene and protein sequence databases.

### Global comparative genomics

We were interested in determining how related the genome of *R. microplus *is to other metazoan genomes. SimiTri [14] was developed for that purpose and is capable of globally comparing a target genome to three other genomes with the results displayed in an easily interpreted triangular graphic. In fact, SimiTri analysis was used to compare EST and whole genome databases from several nematode species, including *C. elegans*, *Haemonchus contortus*, and *Nippostrongylus brasiliensis*, and visualize evolutionary relationships between these nematodes [19]. Hughes et al. [20] used SimiTri analysis for similar purposes in comparisons of translated ESTs from various beetles to the proteomes of *D. melanogaster*, *H. sapiens*, and *C. elegans*. However, since our research priorities are aimed at developing novel control technologies for cattle pests in general and *R. microplus *most specifically, our comparative analyses were guided by these priorities. We wished to use comparative genome analysis to help prioritize selection of possible gene or protein targets for developing novel control technologies, which could include vaccines or design of novel inhibitors aimed at selected gene products. Ideally, a control technology would present no toxicity to non-target organisms, with mammalian toxicity presenting greatest concern. Naturally, an anti-tick control technology which is highly toxic to cattle would be of limited use when applied to cattle compared to an effective approach with high target specificity. Thus we selected the genome of *H. sapiens *as the representative mammalian genome for comparative genome analysis with the BmiGI database, feeling that coding regions without orthologous members in mammals would provider better targets for further investigations. Likewise, as *R. microplus *is an arthropod, we selected the well-characterized genome of *D. melanogaster *for these comparisons. As cattle can be parasitized internally by nematodes, we selected *C. elegans *as a well-studied representative for the genome of that type of organism. Finally, the genome of *A. gambiae *was of interest as this organism is a blood-feeding arthropod vectoring a number of organisms which parasitize human red blood cells in a broadly similar fashion as *B. bovis *and *B. bigemina *parasitize cattle red blood cells.

**Figure 1:**
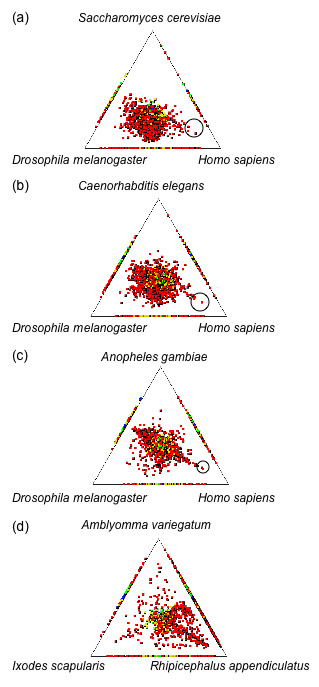
**SimiTri profile of predicted *R. microplus *genes**. The predicted protein-coding region for each *R. microplus *TC or singleton was searched against the protein databases for whole genomes (a-c) using BlastP or tick EST databases (d) using TBlastN (E value < 1 × 10^-8^). Three translated databases are selected for comparison in each profile. The position for each tile represents its similarity to the hits in each different genome as calculated by Blast search raw scores. The color is coded based on the highest Blast score as: red > 300; yellow > 200; green > 150; blue > 100 and purple < 100.

Positional clustering reveals the relationship between genes from *R. microplus *and those from the queried genomes. Additionally, genes which locate along an edge of the triangle have no significant match to the database represented on the opposing vertex of the triangle. Using the conceptually translated sequences of BmiGI, the *R. microplus *data was compared with combinations of data derived from the genome sequences of four other metazoans (*H. sapiens*, *D. melanogaster*, *C. elegans*, and *A. gambiae*) and the unicellular organism *S. cerevisiae *(Figure 1). When compared with *S. cerevisiae*, *D. melanogaster *and *H. sapiens *(Figure 1a), the tick's sequences group closer to *D. melanogaster *than the other two organisms, with the tick appearing most distant from *S. cerevisiae*, an expected result since *S. cerevisiae *is a unicellular organism. Although both *R. microplus *and *D. melanogaster *are arthropods, some *R. microplus *genes appear to be more similar to human genes than *D. melanogaster *as evidenced in Figure 1a. Genes with these atypical gene similarities were selected for more detailed examination of their Blast results and will be discussed later. Upon replacement of *S. cerevisiae *with *C. elegans *(Figure 1b), most of the predicted relationships appear clustered near the center, but careful examination shows slightly greater clustering toward the *D. melanogaster *genome than *C. elegans *or *H. sapiens*. Replacement of *C. elegans *with *A. gambiae *resulted in a roughly symmetrical alignment between *D. melanogaster *and *A. gambiae*, although some atypical genes cluster near *H. sapiens *(Figure 1c). Finally, when the *R. microplus *genes were compared with the three tick EST databases, *R. appendiculatus*, *I. scapularis*, and *A. variegatum*, most genes are clustered closer to *R. appendiculatus *than the other two ticks (Figure 1d). This result is consistent with the phylogenetic classifications of these 4 species of ticks [21].

**Figure 2:**
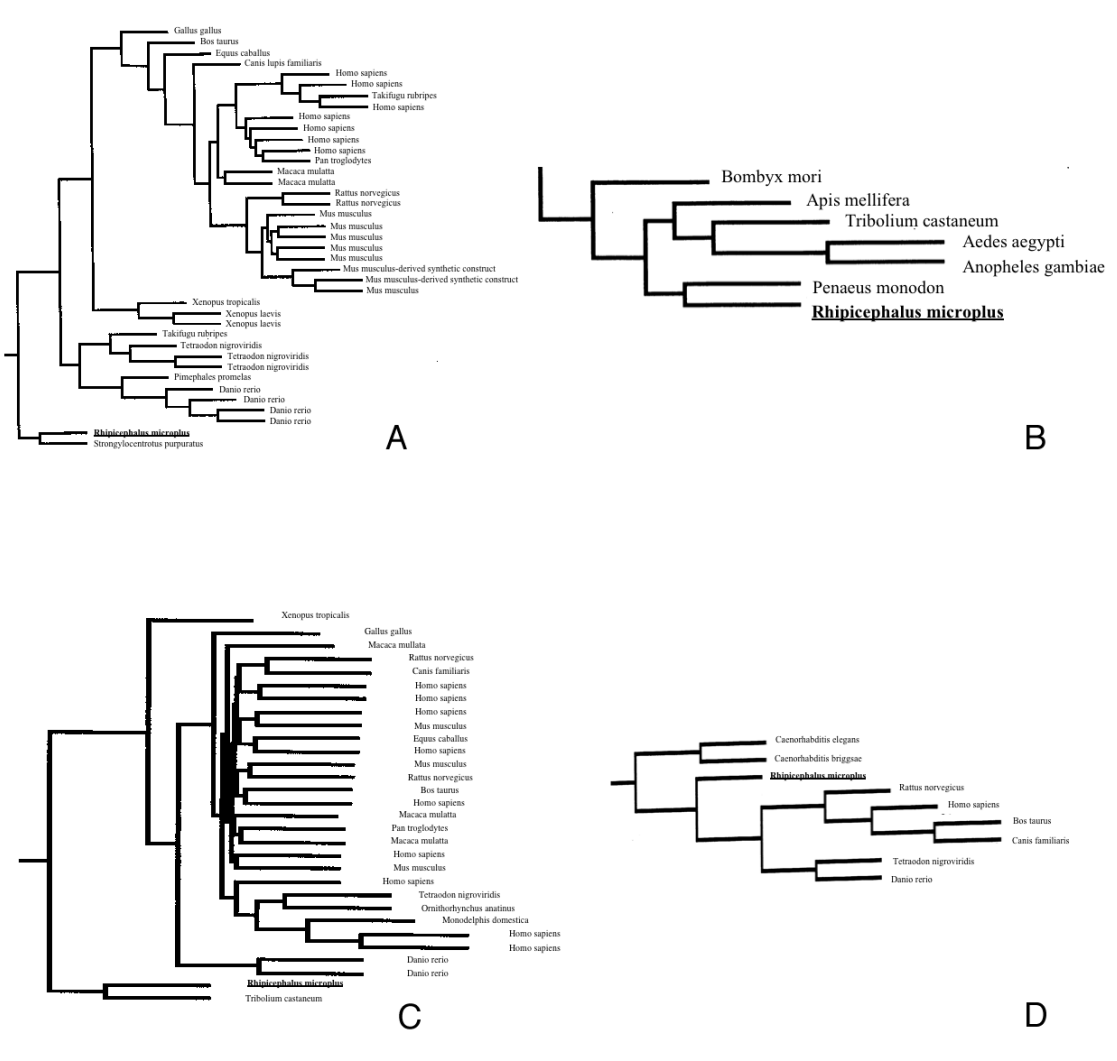
**Phylogenetic tree analysis of atypical genes from SimiTri analysis**. The atypically clustering genes circled in Figure 1a-c were analyzed by BlastP analysis and phylogenetic trees generated with outputs scaled to show distance between sequences. The BmiGI entries were TC14523 (a), TC9268 (b), TC13445 (c) and TC12600 (d) and the TC query sequence is noted in the figure as Rhipicephalus microplus in bold underlined text. The alignments used to generate the trees are included as Additional files 2, 4, 6, 8 in text format. The entire tree is included in pdf file format as Additional files 1, 3, 5, and 7 and only subtrees showing the nearest related sequences are included here.

Phylogenetically, ticks and insects belong to the arthropod phylum and would be expected to share more gene similarity than genes from ticks and mammals. However, SimiTri analysis revealed some atypical genes which were observed to cluster closer to human genes than either *D. melanogaster *or *A. gambiae *genes. The three most atypical genes are circled in Figure 1a–c and listed in Table 2. TC14523 contains the closest similarity to human genes in all the three comparisons of Figure 1a–c. The top Blast hit for TC14523 is Dusty protein kinase (E-Value = 1 × 10^-138^), a protein with a dual functional kinase domain, whose specific biological role has not yet been identified. While most databases classify this protein as a receptor interacting kinase, detailed analysis demonstrated that this is a single copy unique kinase which seems to be present in all vertebrates [22]. An EST [GenBank:CD782617.1] from *R. appendiculatus*, a three host tick parasitizing livestock, shows 91% nucleotide identity to TC14523. Thus, at least two ticks possess this gene and it would be very interesting to know the function of this gene in humans and ticks. Perhaps the function would reveal why the atypical sequence similarity exists for this gene of *R. microplus*. To further investigate the relationship of TC14523 to other Dusty protein kinases with a more powerful method of phylogenetic analysis, we performed a BlastP and Clustal analysis of the conceptual coding region of TC14523 followed by generation of the corresponding phylogenetic tree. Figure 2a shows the coding region of TC14523 is most closely related a Dusty protein kinase from sea urchin with similarities to several Dusty protein kinases from various aquatic species and mammals. In fact, other than two Dusty protein kinases from *Apis mellifera*, arthropod insect sequences seem to be absent from the tree of close relatives to the *R. microplus *sequence (Figure 2a, Additional files 1 and 2). Perhaps this gene has been lost from most insects and the absence of this gene from dipterans would explain why TC14523 clustered near *H. sapiens *in the SimiTri analysis of Figure 1a–c.

**Table 2:** Atyptical genes in SimiTri analysis

	Genes	BlastX hits
Figure 1a	TC14523	Dusty protein kinase (NP_991190)
	TC7573	Destrin (XP534337)
	TC13445	COP1 protein (NP_071902)

Figure 1b	TC14523	Dusty protein kinase (NP_991190)
	TC9268	Syntenin (AAK13497)
	TC13445	COP1 protein (NP_071902)

Figure 1c	TC14523	Dusty protein kinase (NP_991190)
	TC12600	Laminin_A (Q4RST5)
	TC7573	Destrin (XP534337)

Two other atypical genes in Table 2, TC7573 and TC9268 are related to actin binding. The top Blast hit for TC7573 is Destrin (E-Value = 1 × 10^-78^), an actin-depolymerizing factor [23], however, TC7573 shows over 99% nucleotide identity to ESTs from various *Bos taurus *cDNA libraries, including those from skin, liver, and placenta, and it is likely TC7573 is of bovine origin. TC9268 contains 2 PDZ (postsynaptic density protein, disc-large, zonulin-1) domains and has sequence similarity to syntenin (E-Value = 1 × 10^-76^), which is involved in diverse physiological processes resulting from its interaction with signaling and adhesion molecules [24]. An EST [GenBank:CD791887] from *R. appendiculatus *has 85% nucleotide identity in the 5' region of TC9268, so this gene is present in at least two species of ticks. BlastP and phylogenetic tree analysis (Figure 2b) shows the coding region from TC9268 is very similar to coding regions from shrimp, honey bee, red fluor beetle, and several mosquitoes. A *Drosophila *gene does not appear in the phylogenetic tree (Figure 2b, Additional files 3 and 4), indicating a closely related sequence to TC9268 is not present in drosophilids, perhaps being lost from that group of species. In fact, *C. elegans *does not show a close relative of TC9268 and the absence of this gene coding region from both *C. elegans *and *D. melanogaster *would explain its atypical clustering in Figure 1b.

The top Blast hit for TC13445 is COP1 (constitutive photomorphogenic 1; E-Value = 1 × 10^-91^), a protein acting as an E3 ubiquitin ligase involved in light signaling in plants and tumorigenesis in mammals [25]. A *R. appendiculatus *EST [GenBank:CD779568.1] has 94% nucleotide identity to TC13445. The BlastP and tree analysis (Figure 2c) shows that other than a *Tribolium castaneum *orthologue, sequences with high similarity to TC13445 seem to be generally absent from insects. The *T. castaneum *orthologue has the closest relationship to the *R. microplus *gene, and there is significant similarity to orthologues from organisms as diverse as fishes, primates, and several species of plants. Although *D. melanogaster *and *Drosophila pseudoobscura *do have sequences with limited similarity (E-Value = 1 × 10^-13^) to TC13445, the tree analysis showed both *Drosophila *sequences fell somewhat distant from the TC13445 (Figure 2c, Additional files 5 and 6). The nematodes do not appear to have a close relative of TC13445 either and, coupled with the absence of an orthologue in *D. melanogaster *with close similarity to TC13445, helps explain the atypical clustering of this sequence in Figures 1a and 1b.

TC12600 possesses significant sequence similarity to a protein (Q4RST5) that contains a Laminin_A domain (E-Value = 1 × 10^-124^), which exists in the extracellular space, functioning in the signaling process for morphogenesis and also playing a structural role [26]. BlastP analysis shows the closest relatives to the *R. microplus *sequence are zebrafish, pufferfish and various mammals (Figure 2d), with an absence of insect sequences in the phylogenetic tree (Figure 2d, Additional files 7 and 8) indicating this gene may have been lost from most insects. It is possible these atypically clustering sequences, TC14523, TC9268, TC13445, and TC12600, could represent examples of convergent evolution resulting from the parasitic lifestyle of ticks on their mammalian hosts subjecting the tick to similar environmental and evolutionary pressures as mammals. However, the atypical clustering seen in the SimiTri analysis (Figure 1a–c, Table 2) appears to more likely result from gene loss in the arthropod selected as a query genome, an event more easily visualized by phylogenetic tree analysis of aligned sequences from many genomes than SimiTri analysis which is limited to three genomes. Additionally, the dipterans are better represented among arthropods having sequenced genomes or significant collections of ESTs. As more non-dipteran arthropod sequences become available, a better understanding of phylogenetic relationships will develop.

**Figure 3:**
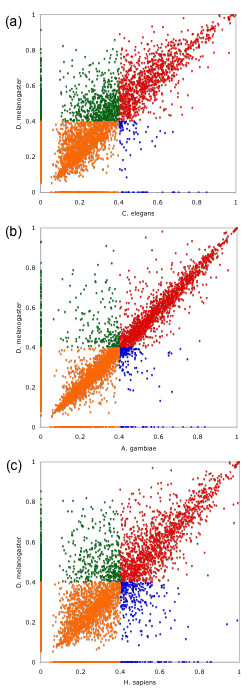
**Comparative genomics analysis of *R. microplus *genes using Blast Score Ratio**. The BLAST score ratio (BSR) approach [13] was adopted to compare the predicted BmiGI genes to other genomes. The quadrant A (orange) represents genes present only in *R. microplus *while genes in quadrant B (green) are most similar to the genome on the y axis. Genes in quadrant C (red) are equally similar to both genomes, and genes in quadrant D (blue) are most similar to genomes on the x axis. The comparisons have been performed using three different genomes on the x axis, *C. elegans *(a), *A. gambiae *(b) and *H. sapiens *(c) against *D. melanogaster *on the y axis.

While SimiTri analysis provides a visualization of the similarity of the target genomes to three other genomes, it has been argued that the calculated distance for each gene, which is normalized using the sum of the blast scores against the three genomes, can only represent the relative distance to the three genomes. It would be preferable to have a measure that is independent of the genome of comparison. Also, the most highly conserved sequences are crowded around the center and there is no clear-cut division for designating conserved genes and genome specific genes. The Blast Score Ratio (BSR) approach was proposed as an improvement to SimiTri analysis and BSR was used to compare the predicted BmiGI genes to those from other genomes [13]. Using BSR and a Blast search E-value of 1 × 10^-8^, the translated BmiGI was compared to the proteomes of *C. elegans, A. gambiae, D. melanogaster *and *H. sapiens *in various combinations (Figure 3). In all three comparisons, *D. melanogaster *was the proteome queried in the Y axis and over 4,000 data points have Blast hits. The coordinates of each data point represent the distances to two different genomes, which are calculated by the Blast score divided by the reference. The reference is the score of each gene Blasted against itself and serves as a normalization factor which distinguishes the BSR approach from SimiTri. A useful check of similarity in the BSR analysis is to divide the plots into quadrants defined by lines extended from both the X and Y axis at the 0.4 mark [13]. The quadrant A (orange) represents genes present only in *R. microplus *while genes in quadrant B (green) are most similar to the genome on the y axis. Genes in quadrant C (red) are equally similar to both genomes, and genes in quadrant D (blue) are most similar to genomes on the x axis. A common feature to all three plots is that more of the data are in the region above a diagonal line of slope = 1, indicating these peptides are more related to those of the proteome along the Y axis, *D. melanogaster*. Approximately 2,300 peptides were consistently found in BSR quadrant A of all three plots, the quadrant closest to the plot's origin, indicating these genes do not have close matches in either of the other two queried proteomes. Also, by comparing the number of peptides most similar to *D. melanogaster *(803 peptides; Figure 3a Quadrant B) to the number most similar to *C. elegans *(93 peptides; Figure 3a Quadrant D), the difference is striking. Not surprisingly, this difference decreases when substituting the proteome of *A. gambiae *for that of *C. elegans *(Figure 3b). In this case 303 and 183 peptides occur in Quadrants B and D, respectively. It can be seen that more points are clustered around the diagonal line in Figure 3b than Figures 3a or 3c, indicating the *R. microplus*, *D. melanogaster*, and *A. gambiae *proteomes present a closer relationship than those used for the other two plots. This also is reflected in the number of points in Quadrant C, which represent genes common to all three proteomes. There are 1113, 1613, and 1367 peptides in Quadrant C of Figure 3a, 3b, and 3c, respectively. Another interesting point from Figure 3c is that the numbers of peptides in Quadrant B (closer to *D. melanogaster*) and D (closer to *H. sapiens*) are 529 and 332 respectively. There are more peptides with closer similarity to *H. sapiens *as opposed to *D. melanogaster *than might be expected based on the phylogenetic distances between the 3 species. By comparison, the BSR analysis with *D. melanogaster *and *C. elegans *(Figure 3) revealed 803 and 93 peptides in Quadrants B and D, respectively. A detailed analysis of the peptides in Quadrant D of Figures 3a and 3c is underway to understand the unexpectedly large number of peptides with greater similarity to the human proteome. It is likely that gene loss events in *D. melanogaster*, *H. sapiens*, and *C. elegans *are affecting this distribution as we discussed earlier in the atypically clustering genes from the SimiTri analysis. It is also of note that BmiGI was derived from ticks which were not treated to remove commensal microbes. Some of these ticks were known to be infected with *B. bovis *and *B. bigemina*, and as noted previously, some sequences in BmiGI appear to be from *B. bovis*. The genome analysis for *B. bovis *was not available at the time BmiGI Version 2 was developed. Although the percentage is expected to be low, some of the "unique" sequences in BSR Quadrant A probably result from these commensal organisms. It is also noteworthy that the atypical genes which SimiTri analysis revealed as more similar to *H. sapiens *than *D. melanogaster *mapped in the Quadrant D region of Figure 3c (closer to *H. sapiens*, data not shown), indicating consistent results from both approaches.

As the comparative analysis was performed with BmiGI, which is an incomplete database of tick gene coding regions, the SimiTri and BSR plots might present different results once the entire tick genome is available for analysis. In fact, once the *I. scapularis *genome is available and annotated [27], a more comprehensive SimiTri and BSR analysis could be easily done and the *I. scapularis *results compared to these presented in our study. It is our feeling that *R. microplus *gene coding regions not currently represented in BmiGI are genes expressed in either highly specialized tissues or at very low levels in the tick. A significant fraction of these genes might be involved in regulatory processes or gene cascades and could have conserved features across a number of arthropod, or even eukaryotic, classes of organisms. These genes would have plotted in the central portions of SimiTri plots and in Quadrant C of BSR plots. However, it is likely that a number of low abundance tick-specific genes have not been discovered during our EST sequencing and would not be in BmiGI. Genes with little or no similarity to those from organisms used in the SimiTri analysis would result in data points along the edges of the plot, while in BSR analysis, these individuals would plot in Quadrant A. Additionally, the Blast analysis E-value can be adjusted to act as a filter on the SimiTri and BSR results. As both SimiTri and BSR only plot sequences which pass the designated Blast E-value cutoff, these comparisons can be made more or less stringent by varying the E-value. If a query sequence does not have a Blast hit to any organism in GenBank, that sequence will not get plotted during either SimiTri or BSR analysis.

BmiGI is composed of 69% assembled TCs and 31% singletons. Thus the prot4EST translation data contains a significant proportion of small proteins resulting from translation of incomplete open reading frames and a 3' end bias is certainly present in EST databases generated from polyA RNA selection methodology as used in deriving BmiGI. In the prot4EST translations of BmiGI, 18% and 11% of the 9,494 TCs yielded translation products of < 80 and < 60 amino acids, respectively. The 4,238 singleton translation product set had 29% and 11% of its members with < 80 and < 60 amino acids, respectively. The combination of the likely 3' end bias of BmiGI and the generally less conserved nature of 3' untranslated regions could contribute to bias results of the BSR analysis toward proteins without matches to the other two queried organisms, thus plotting in Quadrant A. Although this possible bias should be kept in mind, the prot4EST polypeptide prediction pipeline contains ESTscan2.0 to recognize and separate probable protein coding regions from 5' and 3' untranslated regions [28], reducing their impact on the BSR analysis. As discussed in the previous paragraph, Blast E-value will also affect BSR. These small translation products are less likely to have Blast hits to any organism than longer proteins and, if this happens, would not appear on the BSR plots.

### Protein domain analysis

**Figure 4:**
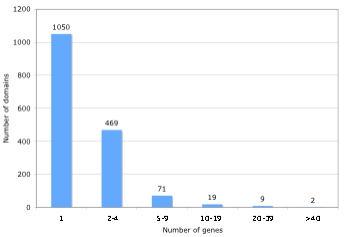
**Distribution of protein domains found in BmiGI2**. The putative translated sequences in BmiGI2 were queried against the Conserved Domain Database using RPS-Blast with a cutoff of E-value < 1 × 10^-8^. The protein domains were grouped based on the number of BmiGI entries that contains the domain in question. The total number of domains in each group is indicated above the bar.

Since the blast search only gives hits for a fraction of BmiGI, we performed a protein domain analysis to extract more information about the coding sequences in the *R. microplus *database. The RPS-Blast searches conducted against the conserved domain database produced 3252 BmiGI entries which have hits representing 1620 unique domains (E-value of 1 × 10^-8^). The distribution of the number of BmiGI entry hits versus number of domains is illustrated in Figure 4, showing that 1050 domains were represented only once in BmiGI, while 2 domains were represented in over 40 BmiGI entries. The ten most common domains were listed in Table 3, and these common domains can be categorized into two groups. One group, which includes WD40, RNA recognition motif (RRM), serine proteinase inhibitor (serpin), and the Kunitz-type protease inhibitor (KU) domains, consists of protein domains that are common structural modules involved in protein-protein interactions. The six other domains are mainly enzymatically functional and include serine/threonine protein kinase catalytic (STKc), mixed function oxidase (P450), protease (Trypsin-like protease, peptidase M13), esterase (carboxyesterase), and Ubiquitin-conjugating enzyme E2 catalytic domains.

**Table 3:** The most abundant domains in BmiGI version 2

Conserved domain	CDD Accession number	Number of hits	Percentage of the total hits
WD40	29257	58	1.78
RNA recognition motif (RRM)	47687	44	1.35
P450	40168	39	1.20
Trypsin like serine protease	29152	34	1.05
STKc kinase	29142	30	0.92
Peptidase M13	41481	29	0.89
Ubiquitin-conjugating enzyme E2, catalytic domain	29157	27	0.83
KU domain	29009	26	0.80
Serine Proteinase Inhibitors (Serpin)	29117	22	0.68
Carboxylesterase	40235	21	0.66

**Figure 5:**
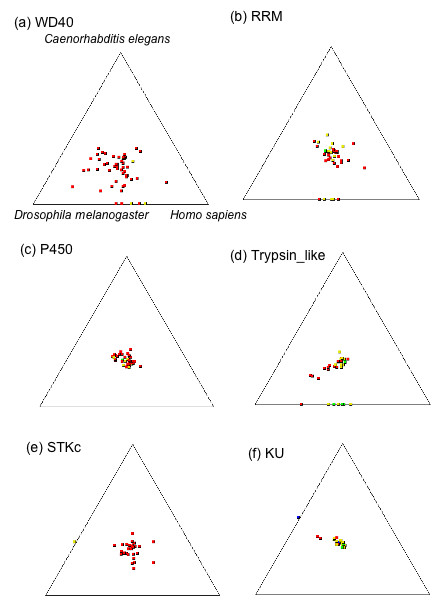
**SimiTri profiles of 6 selected protein domains**. The five most abundant protein domains and the KU domain from RPS-Blast search (Table 3) were chosen for SimiTri profile analysis. The data for the genes in each protein domain was extracted from Figure 1b. The protein domains illustrated are: WD40 in (a), RNA recognition motif in (b), P450 in (c), Trypsin-like protease in (d), Serine/Threonine Kinase catalytic domain in (e) and Kunitz Unit domain in (f).

To further analyze these domains, we examined the SimiTri profile of sequences identified as containing the top five domains of Table 3 by comparing the corresponding sequences from *R. microplus *with the sequences from the proteomes of *C. elegans*, *D. melanogaster *and *H. sapiens *(Figure 5). We also included the KU domain in this analysis because the KU domain has been partially characterized in *I. scapularis *[9]. With the exception of the WD40 domain, most data points are clustered in the center, indicating close similarities among all the proteomes for these sequences. For the WD40, RRM and Trypsin-like domains, some domain family members are not found in *C. elegans*, indicated by the data points aligned on the SimiTri plot line joining *D. melanogaster *and *H. sapiens *(Figure 5a, 5b and 5d). Also, there is possibly divergent evolution in the WD40 sequences as the data are somewhat spread out in the plot (Figure 5a) as opposed to the tighter clustering seen in the other domain sequence plots (Figure 5b–f). Interestingly, for both the STKc and KU domains, one *R. microplus *sequence is found conserved among *D. melanogaster *and *C. elegans *and absent in *H. sapiens*, as it maps to the line joining *D. melanogaster *and *C. elegans *(Figure 5e, f). It is possible the corresponding STKc and KU orthologs have been lost during the evolutionary development of the higher organism, *H. sapiens*. Each of these domains play roles in fundamental cellular processes. For example, WD domains are involved in diverse functions such as G-protein coupling, RNA-processing, vesicular trafficking and cell division [29]. The RRM domain provides a plastic RNA-binding platform to regulate post-transcriptional gene expression [30]. Of particular importance are the KU and protease domains, as these domains are vital to the tick in their role as blood-feeding organisms [31].

## Conclusion

This study presents the analysis of BmiGI Version 2, which contains a significant fraction of the coding regions of the genome of *R. microplus*. Our results indicate that many genes of *R. microplus *are unique and have no homologs in other sequenced genomes. With E-value = 1 × 10^-8^, only 34% of the 13,765 members of BmiGI can be assigned one or more GO terms using this relatively liberal Blast E-value.

Among the BmiGI members which had Blast hits, BSR analysis found approximately 2,300 *R. microplus *sequences which did not have a close match to sequences from *D. melanogaster*, *H. sapiens*, *C. elegans*, or *A. gambiae*. This highlights there have been unique gene evolutionary processes in ticks and emphasizes the importance of sequencing a tick genome to better understand tick biology. In the absence of whole genome sequence [32], EST data is a good resource for gene discovery and will facilitate the study of acaricide resistance mechanisms in *R. microplus*. Our global comparative analysis identified some tick genes with unexpected phylogenetic relationships which detailed analysis attributed to gene losses in some members of the animal kingdom.

## Methods

### *R. microplus *EST sequences

The construction of the *R. microplus *normalized cDNA library, generation of ESTs, and assembly into the *R. microplus *gene index have been described [11]. Briefly, a single normalized cDNA library was synthesized from pooled RNA samples which had been purified from ticks subjected to various environmental exposures, including heat shock, cold shock, host odor, infection with *B. bovis*, and various acaricides. The acaricide exposure experiments were performed with several strains of *R. microplus *which varied in their levels of susceptibility to pyrethroid, organophosphate and the formamidine amitraz. We also included RNA purified from eggs, nymphs, adults and dissected adult tick organs.

### Comparative genomic analysis

The ESTs were clustered and assembled into tentative consensus (TC) sequences using TIGR's autoannotation pipeline tools [33], and non-clustered, non-overlapping sequences defined as singleton sequences. GO terms [34] were assigned automatically using customized script based on BlastX search results. We also used PartiGene [35] as another pipeline for EST analysis, as this open source analytical package provided some powerful annotation options to compare to the TIGR autoannotation pipeline results. BmiGI Version 2 was analyzed by PartiGene and, to maintain consistency, the TC and singleton numerical designations in BmiGI were kept identical. The protein coding regions of *R. microplus *were determined by applying prot4EST [28] to BmiGI and using data from Uniprot for the Blastp [36]. GO terms were assigned based solely on Blast E-values using the annot8r module from the PartiGene package.

For the SimiTri analysis [14], the program was downloaded [37] and BlastP searches were performed using the prot4EST translated sequences from BmiGI and a cutoff E-value < 1 × 10^-8^. Blosum62 was used as the matrix for these searches for its strength in detecting weak similarities between proteins. The predicted proteomes of *S. cerevisiae*, *C. elegans*, *A. gambiae*, *D. melanogaster *and *H. sapiens *were downloaded [38]. TblastN searches were performed against EST databases from the ticks, *R. appendiculatus *and *A. variegatum *[39] and *I. scapularis *[40]. The atypically clustering *R. microplus *genes were analyzed by BlastP analysis of the conceptual open reading frames as noted in BmiGI Version 2 followed by generation of phylogenetic trees using ClustalW [41]and programs from the Phylip phylogeny inference package [42]. In the Phylip package, SEQBOOT was used at default values except with 1000 replicates, PROTPARS at default values except with 10 jumbles, and CONSENSE and at all default values. The consensus trees were viewed using TREEVIEW [43]. Subtrees containing sequences closely related to the BmiGI entry are displayed as figures while the entire tree and the alignment used to produce the tree are included as Additional files 1, 2, 3, 4, 5, 6, 7, 8.

In the BLAST Score Ratio (BSR) approach [13], the conceptual translation of the *R. microplus *reference genome (BmiGI Version 2) was compared with various proteome pairs of *S. cerevisiae*, *C. elegans*, *A. gambiae*, *D. melanogaster *and *H. sapiens *which served as Query_1 _and Query_2_. The graphical output files are plotted and divided into four quadrants using the BSR threshold value of 0.4.

Protein domain analysis were performed by RPS-Blast search against the Conserved Domain Database [44] using a cutoff E-value < 1 × 10^-8^. The translated sequences for the domains of interest were extracted from BmiGI and subjected to SimiTri analysis using the protocols described above.

## Competing interests

The author(s) declares that there are no competing interests.

## Authors' contributions

MW carried out the comparative genome analysis with SimiTri and BSR, implemented the PartiGene pipeline analysis and drafted the manuscript. GP and VMN participated in the bioinformatic analysis with the TIGR pipeline. FG conceived of the study, and participated in its design and coordination and helped to draft and revise the manuscript. All authors read and approved the final manuscript.

## Supplementary Material

Additional file 1 Phylogenetic tree of TC14523. The data provided shows the entire tree generated from the TC14523 alignment Click here for file

Additional file 2 Alignment from Clustal W for TC14523. This file shows the alignment which was used to generate the phylogenetic tree for TC14523 Click here for file

Additional file 3 Phylogenetic tree of TC9268. The data provided shows the entire tree generated from the TC9268 alignment Click here for file

Additional file 4 Alignment from Clustal W for TC9268. This file shows the alignment which was used to generate the phylogenetic tree for TC9268 Click here for file

Additional file 5 Phylogenetic tree of TC13445. The data provided shows the entire tree generated from the TC13445 alignment Click here for file

Additional file 6 Alignment from Clustal W for TC13445. This file shows the alignment which was used to generate the phylogenetic tree for TC13445 Click here for file

Additional file 7 Phylogenetic tree of TC12600. The data provided shows the entire tree generated from the TC12600 alignment Click here for file

Additional file 8 Alignment from Clustal W for TC12600. This file shows the alignment which was used to generate the phylogenetic tree for TC12600 Click here for file
