# Supplementary material for: Global comparative analysis of ESTs from the southern cattle tick, Rhipicephalus (Boophilus) microplus
Source: BMC Genomics. 2007 Oct 12;8:368. doi: 10.1186/1471-2164-8-368 (PMC2100071; doi:10.1186/1471-2164-8-368)
Supplement: Additional file 2 — Alignment from Clustal W for TC14523. This file shows the alignment which was used to generate the phylogenetic tree for TC14523 [file 1471-2164-8-368-S2.pdf]

Alignment view for rid: 8JNGSBU014, query ID: lcl|1_27736, database: nr

Mouse over the sequence identifer for sequence title

```
1_27736         2     RLARCICSQFRERLRSSHEQFQAAMRQLEAXPLGPSGAHRNAKD---QGTKAAPPRLARC  58
AAV40860        573   K...S......T..Y....A.A.SL....ERHT.RLERTEDLWL---RVR.DHA.....L  629
AAH67573        168   K..KS......T..N....A.A.SL....E---.HT.RLERTE.LWLRVR.DHA.....L  224
Q4VSN2          573   K...S......T..N....A.A.SL....ERHT.RLERTEDLWL---RVR.DHA.....L  629
Q4VSN1          542   K..KS......T..N....A.A.SL....E---.HT.RLERTE.LWLRVR.DHA.....L  598
AAP42423        514   K..KS......T..N....A.A.SL....E---.HT.RLERTE.LWLRVR.DHA.....L  570
NP_991190       514   K..KS......T..N....A.A.SL....E---.HT.RLERTE.LWLRVR.DHA.....L  570
NP_001020439    582   K..KS......T..N....A.A.SL.....---.H..RLE.TE.LWLKVR.DHA.....L  638
ABD72471        558   K..KS......T..N....A.A.SL.....---.H..RLEKTE.LWLKVR.DHA.....L  614
NP_001035038    582   K..KS......T..N....A.A.SL.....---.H..RLEKTE.LWLKVR.DHA.....L  638
NP_056190       580   K..KS......T..N....A.A.SL.....---.H..RLEKTE.LWLRVR.DHA.....L  636
NP_001029338    581   K..KS......T..N....A.A.SL.....---.H..RLEKTE.LWLRVR.DHA.....L  637
Q6XUX3          580   K..KS......T..N....A.A.SL.....---.H..RLEKTE.LWLRVR.DHA.....L  636
AAH72406        580   K..KS......T..N....A.A.SL.....---.H..RLEKTE.LWLRVR.DHA.....L  636
NP_001091920    593   K..KS......S..NN...S.STSL....QKHS.RLEKTEEQRM---KVR.VYA.....L  649
EDM09796        480   K..KS......T..N....A.A.SL.....---.H..RLEKTE.LWLKVR.DHA.....L  536
BAD32232        309   K..KS......T..N....A.A.SL.....---.H..RLEKTE.LWLKVR.DHA.....L  365
BAE26444        556   K..KS......T..N....A.A.SL.....---.H..RLEKTE.LWLKVR.DHA.....L  612
NP_955750       578   K..KS......T..N....A.A.SL.....---.H..RLEKTE.LWLKVR.DHA.....L  634
NP_766104       578   K..KS......T..N....A.A.SL.....---.H..RLEKTE.LWLKVR.DHA.....L  634
NP_989837       581   K..KS......T..N....A.A.SL....D---.H..RLEKTE.LWLKVR.DHA.....L  637
EDL39678        578   K..KS......T..N....A.A.SL.....---.H..RLEKTE.LWLKVR.DHA.....L  634
ABK42490        165   K..KS......T..N....A.A.SL.....---.H..RLEKTE.LWLKVR.DHA.....L  221
ABK42489        165   K..KS......T..N....T.A.SL.....---.H.SRLEKTE.LWLKVR.DHA.....L  221
ABD77593        555   K..KS......T..N....A.ASSL....E---.HT.RLERTE.LWLRVR.DHA.....L  611
AAH89380        101   K..KS......T..N....A.A.SL.....---.H..RLEKTE.LWLKVR.DHA.....L  157
XP_001489301    567   K..KS......T..N....A.A.SL.....---.H..RLEKTE.LWLKVR.DHA.....L  623
NP_001019995    579   K..KS......T..N....A.A.SL.....---.H..RLEKTE.LWLKVR.DHA.....L  635
BAA32317        16    K..KS......T..N....A.A.SL.....---.H..RLEKTE.LWLRVR.DHA.....L  72
BAC34316        41    K..KS......T..N....A.A.SL.....---.H..RLEKTE.LWLKVR.DHA.....L  97
AAH97571        569   K..KS.....CK..K....A.A.SLK...---V.H..RLKKTN.LWLRVR.DHA.....L  625
NP_001007477    571   K..KS.....CK..K....A.A.SLK...---V.H..RLEKTN.LWLRVR.DHA.....L  627
NP_001087088    569   K..KS.....CK..K....A.A.SLK...---V.H..RLEKTN.LWLRVR.DHA.....L  625
AAY81956        522   K.SKI.LM..GDK.E...DS....L.SI.NYYS.KLERTEEQRI---ALR.YHA....KL  578
NP_001071281    560   K.SKI.LM..GDK.E...DS....L.SI.NYYS.KLERTEEQRI---ALR.YHA....KL  616
NP_001027827    545   K...S......T..N....A.A.SL....ERHT.RLERTEDLWL---RVR.DHA.....L  601
NP_955749       580   K..KS......T..N....A.A.SL.....---.H..RLEKTE.LWLRVR.DHA.....L  636
AAH53627        580   K..KS......T..N....A.A.SL.....---.H..RLEKTE.LWLRVR.DHA.....L  636
CAG06841        598   K...S......T..Y....A.A.SL....ERHT.RLERTEDLWL---RVR.DHA.....L  654
CAF88742        212   K...S......T..Y....A.A.SL....ERHT.RLERTEDLWL---RVR.DHA.....L  268
XP_001507776    402   K..KS......T..N....A.A.SL.....---.H..RLEKTE.LWLKVR.DHA.....L  458

1_27736         59    ALESTSLRDEILYGMPQLGRELGRGQYGVVYSSEPWGGRS-PCAVK----S---V-----  105
AAV40860        630   S...R....VV.H.K.K..............LCDS...HN-...L.----.---.-----  676
AAH67573        225   S...R....IL.H.K.K..............LCDS.A..H-...L.----.---.-----  271
```

```
Q4VSN2         630   S...R....VL.H.K.K..............LCDN...HY-...L.----.---.-----  676
Q4VSN1         599   S...R....IL.H.K.K..............LCDS.A..H-...L.----.---.-----  645
AAP42423       571   S...R....IL.H.K.K..............LCDS.A..H-...L.----.---.-----  617
NP_991190      571   S...R....IL.H.K.K..............LCDS.A..H-...L.----.---.-----  617
NP_001020439   639   S...R..Q.VL.HRK.K..Q...........LCDN...HF-...L.----.---.-----  685
ABD72471       615   S...R..Q.VL.HRK.K..Q...........LCDN...HF-...L.----.---.-----  661
NP_001035038   639   S...R..Q.VL.HRK.K..Q...........LCDN...HF-...L.----.---.-----  685
NP_056190      637   S...C..Q.VL.HRK.K..Q...........LCDN...HF-...L.----.---.-----  683
NP_001029338   638   S...R..Q.VL.HRK.K..Q...........LCDN...HF-...L.----.---.-----  684
Q6XUX3         637   S...R..Q.VL.HRK.K..Q...........LCDN...HF-...L.----.---.-----  683
AAH72406       637   S...R..Q.VL.HRK.K..Q...........LCDN...HF-...L.----.---.-----  683
NP_001091920   650   .........MV.....K.E..I..........CRS...VT-H....----.---.-----  696
EDM09796       537   S...R..Q.VL.HRK.K..Q...........LCDN...HF-...L.----.---.-----  583
BAD32232       366   S...R..Q.VL.HRK.K..Q...........LCDN...HF-...L.----.---.-----  412
BAE26444       613   S...R..Q.VL.HRK.K..Q...........LCDN...HF-...L.----.---.-----  659
NP_955750      635   S...R..Q.VL.HRK.K..Q...........LCDN...HF-...L.----.---.-----  681
NP_766104      635   S...R..Q.VL.HRK.K..Q...........LCDN...HF-...L.----.---.-----  681
NP_989837      638   S...R..Q.VL.H.K.K..............LCDS...HF-...L.----.---.-----  684
EDL39678       635   S...R..Q.VL.HRK.K..Q...........LCDN...HF-...L.----.---.-----  681
ABK42490       222   S...R..Q.VL.HRK.K..............LCDN...HF-...L.----.---.-----  268
ABK42489       222   S...R..Q.VL.HRK.K..Q...........LCDN...HF-...L.----.---.-----  268
ABD77593       612   S...R....IL.H.K.K..............LCDN.A..H-...L.----.---.-----  658
AAH89380       158   S...R..Q.VL.HRK.K..Q...........LCDN...HF-...L.----.---.-----  204
XP_001489301   624   S...R..Q.VL.HRK.K..Q...........LCDS...HF-...L.----.---.-----  670
NP_001019995   636   S...R..Q.VL.HRK.K..Q...........LCDS...HF-...L.----.---.-----  682
BAA32317       73    S...R..Q.VL.HRK.K..Q...........LCDN...HF-...L.----.---.-----  119
BAC34316       98    S...R..Q.VL.HRK.K..Q...........LCDN...HF-...L.----.---.-----  144
AAH97571       626   S...R..Q.VL.H.K.RI.............LCDS...HF-...L.----.---.-----  672
NP_001007477   628   S...R..Q.VL.H.K.RI.............LCDS...HF-...L.----.---.-----  674
NP_001087088   626   S...R..Q.VL.H.K.RI.............LCDS...HF-...L.----.---.-----  672
AAY81956       579   ......MI.VVR..K.HCAE.I......I.FACDG...KAG...I.----.---.-----  626
NP_001071281   617   ......MI.VVR..K.HCAE.I......I.FACDG...KAG...I.----.---.-----  664
NP_001027827   602   S...R....VL.H.K.K..............LCDN...HY-...L.----.---.-----  648
NP_955749      637   S...C..Q.VL.HRK.K..Q...........LCDN...HF-...L.----.---.-----  683
AAH53627       637   S...R..Q.VL.HRK.K..Q...........LCDN...HF-...L.----.---.-----  683
CAG06841       731                .K.K..............LCDS...HN-...L.----.---.-----  764
CAG06841       655   S...R....VV.H.E.                                              670
CAF88742       345                .K.K..............LCDS...HN-...L.----.---.-----  378
CAF88742       269   S...R....VV.H.E.                                              284
AAH48204       1                              ......LCDN...HF-...L.----.---.-----  22
XP_001507776   459   S...R..Q.VL.HRK.K..............LCDS...HF-...L.----.---.-----  505
ABG54351       17                       ....H.T..S..HGK-.K.-.-DV.I.----R---I-----  42
NP_200569      785                      ....H.T..S..HGK-.K.-.-DV.I.----R---I-----  810
EAZ03683       821                      ....S.TF.T..HGK-.R.-.-DV.I.RIKK.---C-----  850
EAZ39614       900                      ....S.TF.T..HGK-.R.-.-DV.I.RIKK.---C-----  929
AAU87044       50                       ....S.AF.T..HGK-.R.-.-DV.I.RIKK.---C-----  79
XP_640615      336                    V.N.I.S.K..S.-.LGY.L.-T-.V.I.----K---L-----  363
EAY83740       4032                     ....S.TF.T..HGK-.R.-T-DV.I.RIKK.---C-----
```

```
4061
EAZ21079       4076                    ....S.TF.T..HGK-.R.-T-DV.I.RIKK.---C-----
4105
ABG54350       17                      ....S.TF.T..HGK-.R.-.-DV.I.RIKK.---C-----  46
XP_695118      567                   ..QSI...NF.E.F.GRLRSDNT-.V...----A---CREN--  599
NP_001067178   823                     ....S.TF.T..HGK-.R.-T-DV.I.RIKK.---C-----  852
ABK06418       18                      K...S.TF.T..HGK-.R.-T-DV.I.RIKR.---C-----  47
CAN82462       1122                    ....S.TF.T..HGK-.R.-T-DV.I.----R---INDRCF
1152
NP_181050      978                     K...S.TF.T..HGK-.R.-T-DV.I.RIKR.---C-----
1007
AAN33122       561                   ..EQI...NF.E.F.GRLRADNT-.V...-----.CRET-----  593
CAN65102       930                     K...S.TF.T..HGK-.R.-.-DV.I.----R---IKKIC-  959
NP_034324      563                   ..EQI...NF.E.F.GRLRADNT-.V...-----.CRET-----  595
EDL06992       570                   ..EQI...NF.E.F.GRLRADNT-.V...-----.CRET-----  602
XP_341877      563                   ..EQI...NF.E.F.GRLRADNT-.V...-----.CRET-----  595
XP_001498813   348                   ..EQI...NF.E.F.GRLRADNT-LV...-----.CRET-----  380
ABE86676       905                      ...S.TF.T..HGK-.R.-T-DV.I.RIKN.---C-----  933
XP_851836      561                   ..EQI...NF.E.F.GRLRADNT-LV...-----.CRET-----  593
XP_001169294   435                   ..EQI...NF.E.F.GRLRADNT-LV...-----.CRET-----  467

1_27736        106   --VP------PDDKH--WNDLAMEFFYTR---SVP-----D---HDRIVQIRGSVIDHNY  144
AAV40860       677   --..------.....--.....L..H...---TL.-----K---.E.L.DLH......T.  715
AAH67573       272   --..------.....--.....L..H...---.L.-----K---.E.L.NLH......S.  310
Q4VSN2         677   --..------.....--.....L..H...---TL.-----K---.E.L.DLH......T.  715
Q4VSN1         646   --..------.....--.....L..H...---.L.-----K---.E.L.NLH......S.  684
AAP42423       618   --..------.....--.....L..H...---.L.-----K---.E.L.NLH......S.  656
NP_991190      618   --..------.....--.....L..H...---.L.-----K---.E.L.NLH......S.  656
NP_001020439   686   --..------..E..--.....L..H.M.---.L.-----K---.E.L.DLH.....Y..  724
ABD72471       662   --..------..E..--.....L..H.M.---.L.-----K---.E.L.DLH.....Y..  700
NP_001035038   686   --..------..E..--.....L..H.M.---.L.-----K---.E.L.DLH.....Y..  724
NP_056190      684   --..------..E..--.....L..H.M.---.L.-----K---.E.L.DLH.....Y..  722
NP_001029338   685   --..------..E..--.....L..H.M.---.L.-----K---.E.L.DLH.....Y..  723
Q6XUX3         684   --..------..E..--.....L..H.M.---.L.-----K---.E.L.DLH.....Y..  722
AAH72406       684   --..------..E..--.....L..H.M.---.L.-----K---.E.L.DLH.....Y..  722
NP_001091920   697   --..------.....--........H...---.IA-----E---.....AVI......G.  735
EDM09796       584   --..------..E..--.....L..H.M.---.L.-----K---.E.L.DLH.....Y..  622
BAD32232       413   --..------..E..--.....L..H.M.---.L.-----K---.E.L.DLH.....Y..  451
BAE26444       660   --..------..E..--.....L..H.M.---.L.-----K---.E.L.DLH.....Y..  698
NP_955750      682   --..------..E..--.....L..H.M.---.L.-----K---.E.L.DLH.....Y..  720
NP_766104      682   --..------..E..--.....L..H.M.---.L.-----K---.E.L.DLH.....Y..  720
NP_989837      685   --..------..E..--.....L..H.M.---.LQ-----S---.E.L.DLH.....YG.  723
EDL39678       682   --..------..E..--.....L..H.M.---.L.-----K---.E.L.DLH.....Y..  720
ABK42490       269   --..------..E..--..G..L..H.M.---.L.-----K---.E.L.DLH.....Y..  307
ABK42489       269   --..------..E..--.....L..H.M.---.L.-----K---.E.L.DLH.....Y..  307
ABD77593       659   --..------.....--.....L..H...---.L.-----K---.E.L.NLH......S.  697
AAH89380       205   --..------..E..--.....L..H.M.---.L.-----K---.E.L.DLH.....Y..  243
XP_001489301   671   --..------..E..--.....L..H.M.---.L.-----K---.E.L.DLH.....YS.  709
```

```
NP_001019995   683   --..------..E..--.....L..H.M.---.L.-----K---.E.L.DLH.....YS.  721
BAA32317       120   --..------..E..--.....L..H.M.---.L.-----K---.E.L.DLH.....Y..  158
BAC34316       145   --..------..E..--.....L..H.M.---.L.-----K---.E.L.DLH.....Y..  183
AAH97571       673   --..------..E..--.....L..H.M.---.L.-----K---.E.L.DLH.....YS.  711
NP_001007477   675   --..------..E..--.....L..H.M.---.L.-----K---.E.L.DLH.....YS.  713
NP_001087088   673   --..------..E..--.....L..H.M.---.L.-----K---.E.L.DLH.....YS.  711
AAY81956       627   --..------S.ES.--.........Y.N.---.I.-----.---.K...KL...I...S.  665
NP_001071281   665   --..------S.ES.--.........Y.N.---.I.-----.---.K...KL...I...S.  703
NP_001027827   649   --..------.....--.....L..H...---TL.-----K---.E.L.DLH......T.  687
NP_955749      684   --..------..E..--.....L..H.M.---.L.-----K---.E.L.DLH.....Y..  722
AAH53627       684   --..------..E..--.....L..H.M.---.L.-----K---.E.L.DLH.....Y..  722
CAG06841       765   --..------.....--.....L..H...---TL.-----K---.E.L.DLH......T.  803
CAF88742       379   --..------.....--.....L..H...---TL.-----K---.E.L.DLH......T.  417
XP_969042      512                              SK---TIS-----E---.P...KL......YT.  533
AAH48204       23    --..------..E..--.....L..H.M.---.L.-----K---.E.L.DLH.....Y..  61
XP_001507776   506   --..------..E..--.....L..H.M.---.L.-----K---.E.L.DLH.....YG.  544
ABG54351       43    --KASCFAGK.SERERLIE.FWK.ALLLS---.L------H---.PNV.SFY.I.R.GPD  88
NP_200569      811   --KASCFAGK.SERERLIE.FWK.ALLLS---.L------H---.PNV.SFY.I.R.GPD  856
EAZ03683       851   --FT------GRSSE--LER..N..WREA---EIL-----SKLH.PNV.AFY.V.K.G--  890
EAZ39614       930   --FT------GRSSE--LER..N..WREA---EIL-----SKLH.PNV.AFY.V.K.G--  969
AAU87044       80    --FT------GRSSE--LER..H..WREA---EIL-----SKLH.PNV.AFY.V.K.G--  119
XP_640615      364   --------------.--E.NEET.ILVQ.---ELQILKEIR---.PQ...FL.--VSR.-  398
EAY83740       4062  --FA------GRSSE--QEK.TKD.WREA---QIL-----SKLH.PNV.AFY.V.P.GT-
4102
EAZ21079       4106  --FA------GRSSE--QEK.TKD.WREA---QIL-----SKLH.PNV.AFY.V.P.GT-
4146
ABG54350       47    --FA------GRSSE--QER.TG..WGEA---EIL-----SKLH.PNV.AFY.V.K.G--  86
XP_695118      600   --L.------AE--.--K.KFL..ARILK---QY------.---.PN..KLI.VCTQKQ-  634
NP_001067178   853   --FA------GRSSE--QEK.TKD.WREA---QIL-----SKLH.PNV.AFY.V.P.GT-  893
ABK06418       48    --FI------GRSSE--QER.TS..WHEA---EIL-----SKLH.PNVMAFY.V.K.G--  87
CAN82462       1153  AGK.------SEQER--MR.----D.WNE---AIKLADL-H---.PNV.AFY.V.L.G--
1191
NP_181050      1008  --FI------GRSSE--QER.TS..WHEA---EIL-----SKLH.PNVMAFY.V.K.G--
1047
AAN33122       594   --L.------..--------.KAK.LQEA---RILKQY--N---.PN..RLI.VCTQKQ-  628
CAN65102       960   --FT------SRSSE--QER.TI..WREA---DIL-----SKLH.PNV.AFY.V.--.DG  999
NP_034324      596   --L.------..--------.KAK.LQEA---RILKQY--N---.PN..RLI.VCTQKQ-  630
EDL06992       603   --L.------..--------.KAK.LQEA---RILKQY--N---.PN..RLI.VCTQKQ-  637
XP_341877      596   --L.------..--------.KAK.LQEA---RILKQY--N---.PN..RLI.VCTQKQ-  630
XP_001498813   381   --L.------..--------.KAK.LQEA---RILKQY--S---.PN..RLI.VCTQKQ-  415
ABE86676       934   --FA------GRFSE--QER.TKD.WREAKIL.TL-----H---.PNV.AFY.V.P.G--  973
XP_851836      594   --L.------..--------.KAK.LQEA---RILKQY--S---.PN..RLI.VCTQKQ-  628
XP_001169294   468   --L.------..--------.KAK.LQEA---RILKQY--S---.PN..RLI.VCTQKQ-  502

1_27736        145   AG-GT--T--PAV--LLVM------DR-------MQK-DLYTALR---A---G---LSWP  174
AAV40860       716   ..-.S--S--I..--..I.------E.-------LHR-....G.K---.---.---..LQ  745
AAH67573       311   S.-.S--S--I..--..I.------E.-------LHR-....G.K---.---.---..LK  340
Q4VSN2         716   ..-.S--S--I..--..I.------E.-------LHR-....G.K---.---.---..LQ  745
```

```
Q4VSN1          685   S.-.S--S--I..--..I.------E.-------LHR-....G.K---.---.---..LK  714
AAP42423        657   S.-.S--S--I..--..I.------E.-------LHR-....G.K---.---.---..LK  686
NP_991190       657   S.-.S--S--I..--..I.------E.-------LHR-....G.K---.---.---..LK  686
NP_001020439    725   G.-.S--S--I..--..I.------E.-------LHR-....G.K---.---.---.TLE  754
ABD72471        701   G.-.S--S--I..--..I.------E.-------LHR-....G.K---.---.---.TLE  730
NP_001035038    725   G.-.S--S--I..--..I.------E.-------LHR-....G.K---.---.---.TLE  754
NP_056190       723   G.-.S--S--I..--..I.------E.-------LHR-....G.K---.---.---.TLE  752
NP_001029338    724   G.-.S--S--I..--..I.------E.-------LHR-....G.K---.---.---.TLE  753
Q6XUX3          723   G.-.S--S--I..--..I.------E.-------LHR-....G.K---.---.---.TLE  752
AAH72406        723   G.-.S--S--I..--..I.------E.-------LHR-....G.K---.---.---.TLE  752
NP_001091920    736   G.M.C--S--....--..L.------E.--------..R-..H..IK---.---N---MEL.  766
EDM09796        623   G.-.S--S--V..--..I.------E.-------LHR-....G.K---.---.---..LE  652
BAD32232        452   G.-.S--S--V..--..I.------E.-------LHR-....G.K---.---.---.TLE  481
BAE26444        699   G.-.S--S--V..--..I.------E.-------LHR-....G.K---.---.---.TLE  728
NP_955750       721   G.-.S--S--V..--..I.------E.-------LHR-....G.K---.---.---..LE  750
NP_766104       721   G.-.S--S--V..--..I.------E.-------LHR-....G.K---.---.---.TLE  750
NP_989837       724   G.-.S--S--I..--..I.------E.-------LHR-....G.K---.---.---.ELE  753
EDL39678        721   G.-.S--S--V..--..I.------E.-------LHR-.F..G.K---.---.---.TLE  750
ABK42490        308   G.-.S--S--V..--..I.------E.-------LHR-.F..G.K---.---.---.TLE  337
ABK42489        308   G.-.S--S--V..--..I.------E.-------LHR-.F..G.K---.---.---.TLE  337
ABD77593        698   G.-.S--S--I..--..I.------E.-------LHR-....G.K---.---.---.VLK  727
AAH89380        244   G.-.S--S--V..--..I.------E.-------LHR-....G.K---.---.---.TLE  273
XP_001489301    710   G.-.S--S--I..--..I.------E.-------LHR-....G.K---.---.---.TLE  739
NP_001019995    722   G.-.S--S--I..--..I.------E.-------LHR-....G.K---.---.---.ALE  751
BAA32317        159   G.-.S--S--I..--..I.------E.-------LHR-....G.K---.---.---.TLE  188
BAC34316        184   G.-.S--S--V..--..I.------E.-------LHR-....G.K---.---.---.TLE  213
AAH97571        712   G.-.S--S--I..--..IT------E.-------LHR-...VG.K---T---.---.TLE  741
NP_001007477    714   G.-.S--S--I..--..IT------E.-------LHR-...VG.K---T---.---..LE  743
NP_001087088    712   G.-.S--S--I..--..IT------E.-------LHR-...VG.K---T---.---..LE  741
AAY81956        666   G.-.F--GFGS..--..IS------.-------LSR-...CGI.---.---.---...L  697
NP_001071281    704   G.-.F--GFGS..--..IS------.-------LSR-...CGI.---.---.---...L  735
NP_001027827    688   ..-.S--S--I..--..I.------E.-------LHR-....G.K---.---.---..LQ  717
NP_955749       723   G.-.S--S--I..--..I.------E.-------LHR-....G.K---.---.---.TLE  752
AAH53627        723   G.-.S--S--I..--..I.------E.-------LHR-....G.K---.---.---.TLE  752
CAG06841        804   ..-.S--S--I..--..I.------E.-------LHR-....G.K---.---.---..LQ  833
CAF88742        418   ..-.S--S--I..--..I.------E.-------LHR-....G.K---.---.---..LQ  447
XP_969042       534   G.-.S--S--....--..I.------E.-------.TR-..HCG..---N---.---...V  563
AAH48204        62    G.-.S--S--I..--..I.------E.-------LHR-....G.K---.---.---.TLE  91
XP_001507776    545   G.-.S--S--I..--..I.------E.-------LHR-....G.K---T---S---SHH.  574
ABG54351        89    GS-LA--.--V.E--FM.N------GS-------LKQ-F.QKKD.---T-------IDRR  117
NP_200569       857   GS-LA--.--V.E--FM.N------GS-------LKQ-F.QKKD.---T-------IDRR  885
EAZ03683        891   P.-..--L--AT.--TEF.------VNGSLRHVLQR.-.K.LDR.---K-----------  922
XP_001308850    304                                                               .  304
EAZ39614        970   P.-..--L--AT.--TEF.------VNGSLRHVLQR.-.K.LDR.---K-----------
1001
AAU87044        120   P.-..--L--AT.--TEF.------VNGSLRHVLQR.-.K.LDR.---K-----------  151
XP_640615       399   ----E--K--DEI--HIIT------EF-------.DGG..FD..I---F---.DIP.T.K  429
EAY83740        4103  -.-..--L--AT.TEFM.N------GS-------LRN-V.LRKD.---M-------.DRR
```

```
4132
EAZ21079      4147  -.-..--L--AT.TEFM.N------GS-------LRN-V.LRKD.---M-------.DRR
4176
XP_001320204  254                                          ..HK..---N---NPEQ.NPT  268
ABG54350      87    P.-..--L--AT.--TEY.V-----.G-------SLR-HVLVRKD---R---H---.DRR  117
XP_695118     635   -----------PI--YII.------EL-------V.GG.FL.F..---TEGHN---.KSS  662
NP_001067178  894   -.-..--L--AT.TEFM.N------GS-------LRN-V.LRKD.---M-------.DRR  923
XP_001309559  317                                                              .  317
ABK06418      88    P.-..--L--AT.--TEY.---------------VNG-S.RHV.L---S---NRH-.DRR  117
CAN82462      1192  P.-.S--V--AT.--TEY.VNGSLRNS-------L..-N-----E---K---N---.DKR
1222
XP_001327096  290                                          ..AL..---T---KKP-I.AS  303
NP_181050     1048  P.-..--L--AT.--TEY.---------------VNG-S.RHV.L---S---NRH-.DRR
1077
AAN33122      629   -----------PI--YI..------EL-------V.GG.FL.F..------------TEG  650
CAN65102      1000  P.-A.--L--AT.--TEY.------VDGSLRHVLLR.-.R.LDR.---K-----------
1031
NP_034324     631   -----------PI--YI..------EL-------V.GG.FL.F..------------TEG  652
EDL06992      638   -----------PI--YI..------EL-------V.GG.FL.F..------------TEG  659
XP_341877     631   -----------PI--YI..------EL-------V.GG.FL.F..------------TEG  652
XP_001498813  416   -----------PI--YI..------EL-------V.GG.FL.F..TEG.---R---.RMK  443
ABE86676      974   P.-..LA.--V.E--YM.H------GS-------LRN-V.LKKE.---V-------.DRR
1004
XP_851836     629   -----------PI--YI..------EL-------V.GG.FL.F..TEG.---R---.RMK  656
XP_001169294  503   -----------PI--YI..------EL-------V.GG.FL.F..------------TEG  524

1_27736       175   ARLR-VAL------DVVQGIRFLHSQGLVHRDIKLKNVLLDRADR----A----KLTDLG  219
AAV40860      746   E..Q-I..------...E..........L............KQN.----.----.I....  790
AAH67573      341   E..L-I..------...E..........L............KQN.----.----.I....  385
Q4VSN2        746   E..Q-I..------...E......N...L............KQN.----.----.I....  790
Q4VSN1        715   E..L-I..------...E..........L............KQN.----.----.I....  759
AAP42423      687   E..L-I..------...E..........L............KQN.----.----.I....  731
NP_991190     687   E..L-I..------...E..........L............KQN.----.----.I....  731
NP_001020439  755   T..Q-I..------...E.......................KQN.----.----.I....  799
ABD72471      731   T..Q-I..------...E.......................KQN.----.----.I....  775
NP_001035038  755   T..Q-I..------...E.......................KQN.----.----.I....  799
NP_056190     753   T..Q-I..------...E.......................KQN.----.----.I....  797
NP_001029338  754   T..Q-I..------...E.......................KQN.----.----.I....  798
Q6XUX3        753   T..Q-I..------...E.......................KQN.----.----.I....  797
AAH72406      753   T..Q-I..------...E.......................KQN.----.----.I....  797
NP_001091920  767   E..H-...------..AE.V.Y...L...............KH..----G----.I....  811
EDM09796      653   T..Q-I..------...E.......................KQN.----.----.I....  697
BAD32232      482   T..Q-I..------...E.......................KQN.----.----.I....  526
BAE26444      729   T..Q-I..------...E.......................KQN.----.----.I....  773
NP_955750     751   T..Q-I..------...E.......................KQN.----.----.I....  795
NP_766104     751   T..Q-I..------...E.......................KQN.----.----.I....  795
NP_989837     754   T..Q-I..------...E...Y...................KKN.----.----.I....  798
EDL39678      751   T..Q-I..------...E.......................KQN.----.----.I....  795
```

```
ABK42490      338   T..Q-I..------...E.......................KQN.----.----.I....  382
ABK42489      338   T..Q-I..------...E.......................KQN.----.----.I....  382
ABD77593      728   E..Q-I..------...E......G...L............KQN.----.----.I....  772
AAH89380      274   T..Q-I..------...E.......................KQN.----.----.I....  318
XP_001489301  740   T..Q-I..------...E.V.....................KQN.----.----.I....  784
NP_001019995  752   T..Q-I..------...E..............V........KQN.----.----.I....  796
BAA32317      189   T..Q-I..------...E.......................KQN.----.----.I....  233
BAC34316      214   T..Q-I..------...E.......................KQN.----.----.I....  258
AAH97571      742   T..Q-I..------...E......N................KKH.----.----.I....  786
NP_001007477  744   T..Q-I..------...E......N................KKH.----.----.I....  788
NP_001087088  742   T..Q-I..------...E......N..V.............KKH.----.----.I....  786
AAY81956      698   E.IQ-I..------..LE...Y..........V........IEN.----.----....F.  742
NP_001071281  736   E.IQ-I..------..LE...Y..........V........IEN.----.----....F.  780
NP_001027827  718   E..Q-I..------...E......N...L............KQN.----.----.I....  762
NP_955749     753   T..Q-I..------...E.......................KQN.----.----.I....  797
AAH53627      753   T..Q-I..------...E.......................KQN.----.----.I....  797
CAG06841      834   E..Q-I..------...E..........L............KQN.----.----.I....  878
CAF88742      448   E..Q-I..------...E..........L............KQN.----.----.I....  492
XP_969042     564   V...-I.I------...E...Y...................GE..----.----....F.  608
AAH48204      92    T..Q-I..------...E.......................KQN.----.----.I....  136
ABG54351      118   K..I-I.M------.TAF.MEY..GKNI..F.L.CE.L.VNMR.PQRPIC----.IG...  166
NP_200569     886   K..I-I.M------.TAF.MEY..GKNI..F.L.CE.L.VNMR.PQRPIC----.IG...  934
EAZ03683      923   -..I-I.M------.AAF.LEY...KNI..F.L.CD.L.VNLK.Q----SRPIC.VG.F.  970
XP_001308850  305   TD.T-I..Y-----..AR.MK...A.NII...L.TL...I.DKK.----.----..S.F.  350
EAZ39614      1002  -..I-I.M------.AAF.LEY...KNI..F.L.CD.L.VNLK.Q----SRPIC.VG.F.
1049
AAU87044      152   -..I-I.M------.AAF.LEY...KNI..V.L.CD.L.VNLK.Q----SRPIC.VG.F.  199
XP_640615     430   EK..-IS.------.IA.SC....AR.IL...L.SQ.I..STNR.----.----..C...  474
EAY83740      4133  K..I-I.M------.AAF.MEY...KSI..F.L.CD.L.VNLR.PQRPIC----.VG.F.
4181
EAZ21079      4177  K..I-I.M------.AAF.MEY...KSI..F.L.CD.L.VNLR.PQRPIC----.VG.F.
4225
XP_001320204  269   T.SL-I..------T.AR.LEY...K.VI...L.SL.....DNNN----.----.IC.F.  313
ABG54350      118   K..I-I.M------.AAF.MEY...KNT..F.L.CD.L.VNLK.P----SRPIC.VG.F.  166
XP_695118     663   MLI.-M.E------N.AS.LAY.E.KKCI...VAAR.C.VGEESV----V----.IS.F.  707
NP_001067178  924   K..I-I.M------.AAF.MEY...KSI..F.L.CD.L.VNLR.PQRPIC----.VG.F.  972
XP_001309559  318   ...TAL.Y------Q.AS.MA....N.I....L.TM.I...ES.A----.----RIA.F.  363
ABK06418      118   K..I-I.M------.AAF.MEY...KSI..F.L.CD.L.VNLK.P----.RPIC.VG.F.  166
CAN82462      1223  K..L-I.M------..AF.MEY..GKNI..F.L.SD.L.VNLR.PHRPIC----.VG...
1271
XP_001327096  304   KKTS-I.F------.IAR.MNY...RHII...L.SP.....DNG.----.----.IC.F.  348
NP_181050     1078  K..I-I.M------.AAF.MEY...KSI..F.L.CD.L.VNLK.P----.RPIC.VG.F.
1126
AAN33122      651   ....-.KTLLQMVG.AAA.MEY.E.KCCI...LAAR.C.VTEKNV----L----.IS.F.  701
CAN65102      1032  -..L-I.M------.AAF.MEY...KNI..F.L.CD.L.VNLK.PLRPIC----.VG.F.
1079
NP_034324     653   ....-.KTLLQMVG.AAA.MEY.E.KCCI...LAAR.C.VTEKNV----L----.IS.F.  703
EDL06992      660   ....-.KTLLQMVG.AAA.MEY.E.KCCI...LAAR.C.VTEKNV----L----.IS.F.  710
```

```
XP_341877      653   ....-.KTLLQMVG.AAA.MEY.E.KCCI...LAAR.C.VTEKNV----L----.IS.F.  703
XP_001498813   444   TL.Q-MVG------.AAA.MEY.E.KCCI...LAAR.C.VTEKNV----L----.IS.F.  488
ABE86676       1005  K.IM-I.M------.AAF.MEY..LKNI..F.L.CD.L.VNLG.PERPVC----.VG.F.
1053
XP_851836      657   TL.Q-MVG------.AAA.MEY.E.KCCI...LAAR.C.VTEKNV----L----.IS.F.  701
XP_001169294   525   ....-.KTLLQMVG.AAA.MEY.E.KCCI...LAAR.C.VTEKNV----L----.IS.F.  575

1_27736        220   F--C---K---P--E--A----M--MSGSI------VGTPIH-MAPE-L-F--T------  244
AAV40860       791   .--.---.---.--.--.----.--.....------......-....-.-.--.------  815
AAH67573       386   .--.---.---.--.--.----.--.....------......-....-.-.--.------  410
Q4VSN2         791   .--.---.---.--.--.----.--.....------......-....-.-.--.------  815
Q4VSN1         760   .--.---.---.--.--.----.--.....------......-....-.-.--.------  784
AAP42423       732   .--.---.---.--.--.----.--.....------......-....-.-.--.------  756
NP_991190      732   .--.---.---.--.--.----.--.....------......-....-.-.--.------  756
NP_001020439   800   .--.---.---.--.--.----.--.....------......-....-.-.--.------  824
ABD72471       776   .--.---.---.--.--.----.--.....------......-....-.-.--.------  800
NP_001035038   800   .--.---.---.--.--.----.--.....------......-....-.-.--.------  824
NP_056190      798   .--.---.---.--.--.----.--.....------......-....-.-.--.------  822
NP_001029338   799   .--.---.---.--.--.----.--.....------......-....-.-.--.------  823
Q6XUX3         798   .--.---.---.--.--.----.--.....------......-....-.-.--.------  822
AAH72406       798   .--.---.---.--.--.----.--.....------......-....-.-.--.------  822
NP_001091920   812   .--.---.---.--.--.----.--.....------......-....-.-.--S------  836
EDM09796       698   .--.---.---.--.--.----.--.....------......-....-.-.--.------  722
BAD32232       527   .--.---.---.--.--.----.--.....------......-....-.-.--.------  551
BAE26444       774   .--.---.---.--.--.----.--.....------......-....-.-.--.------  798
NP_955750      796   .--.---.---.--.--.----.--.....------......-....-.-.--.------  820
NP_766104      796   .--.---.---.--.--.----.--.....------......-....-.-.--.------  820
NP_989837      799   .--.---.---.--.--.----.--.....------......-....-.-.--.------  823
EDL39678       796   .--.---.---.--.--.----.--.....------......-....-.-.--.------  820
ABK42490       383   .--.---.---.--.--.----.--.....------......-....-.-.--.------  407
ABK42489       383   .--.---.---.--.--.----.--.....------......-....-.-.--.------  407
ABD77593       773   .--.---.---.--.--.----.--.....------......-....-.-.--.------  797
AAH89380       319   .--.---.---.--.--.----.--.....------......-....-.-.--.------  343
XP_001489301   785   .--.---.---.--.--.----.--.....------......-....-.-.--.------  809
NP_001019995   797   .--.---.---.--.--.----.--.....------......-....-.-.--.------  821
BAA32317       234   .--.---.---.--.--.----.--.....------......-....-.-.--.------  258
BAC34316       259   .--.---.---.--.--.----.--.....------......-....-.-.--.------  283
AAH97571       787   .--.---.---.--.--.----.--.....------......-....-.-.--S------  811
NP_001007477   789   .--.---.---.--.--.----.--.....------......-....-.-.--S------  813
NP_001087088   787   .--.---.---.--.--.----.--.....------......-....-.-.--S------  811
AAY81956       743   .--.---I---T--.--V----.--.L...------...V..-....-.-L--S------  767
NP_001071281   781   .--.---I---T--.--V----.--.L...------...V..-....-.-L--S------  805
NP_001027827   763   .--.---.---.--.--.----.--.....------......-....-.-.--.------  787
NP_955749      798   .--.---.---.--.--.----.--.....------......-....-.-.--.------  822
AAH53627       798   .--.---.---.--.--.----.--.....------......-....-.-.--.------  822
CAG06841       879   .--.---.---.--.--.----.--.....------......-....-.-.--.------  903
CAF88742       493   .--.---.---.--.--.----.--.....------......-....-.-.--.------  517
XP_969042      609   .--.---I---.--.--.----.--....V------...V..-....-.-L--S------  633
```

```
AAH48204       137   .--.---.---.--.--.----.--.....------......-....-.-.--.------  161
ABG54351       167   LSKV---.---Q--K--T----L--V..GV------R..LPW-....-.-L--S------  193
NP_200569      935   LSKV---.---Q--K--T----L--V..GV------R..LPW-....-.-L--S------  961
EAZ03683       971   L--S---.---I--KRNT----L--V..GV------R..LPW-....-.-L--N------  997
XP_001308850   351   .--S---.QMDS--N--Q----L--.TMN-------....HW-....-.-L--ANNGPQT  383
EAZ39614       1050  L--S---.---I--KRNT----L--V..GV------R..LPW-....-.-L--N------
1076
AAU87044       200   L--S---.---I--KRNT----L--A..GV------R..LPW-....-.-L--N------  226
XP_640615      475   L--A---R--------------.--FEER.NKRYTC...E.W-....-VCL--D------  503
EAY83740       4182  L--SRI-.---R--N--T----L--V..GV------R..LPW-....-.-L--N------
4208
EAZ21079       4226  L--SRI-.---R--N--T----L--V..GV------R..LPW-....-.-L--N------
4252
XP_001320204   314   M--V---R---T--R--D----SRP.T.-M------I..VHW-....-V-LMS.------  341
ABG54350       167   L--S---.---I--KRNT----L--V..GV------R..LPW-....-.-L--N------  193
XP_695118      708   M--S---R---E--.--QDGVYS--AT.GM------KQI.VKWT...A.-N--Y------  738
NP_001067178   973   L--SRI-.---R--N--T----L--V..GV------R..LPW-....-.-L--N------  999
XP_001309559   364   L--SGNMR---D--N--K----D--LY.TL-------...HY-T...-V-L--AR-----  391
ABK06418       167   L--S---.---I--KRNT----L--VT.GV------R..LPW-....-.-L--S------  193
CAN82462       1272  L--S---.---VKCQ--T----L--I..GV------R..LPW-....-.-L--N------
1298
XP_001327096   349   Y--S---RVA-D--D--T----D--VMTKN------....HW-....-.-L--D------  375
NP_181050      1127  L--S---.---I--KRNT----L--VT.GV------R..LPW-....-.-L--S------
1153
AAN33122       702   M--S---R---E--.--.DGIYA--A.AGL------RQV.VKWT...A.-N--Y------  732
CAN65102       1080  L--S---.---I--KRNT----L--V..GV------R..LPW-....-.-L--N------
1106
NP_034324      704   M--S---R---E--.--.DGIYA--A.AGL------RQV.VKWT...A.-N--Y------  734
EDL06992       711   M--S---R---E--.--.DGIYA--A.AGL------RQV.VKWT...A.-N--Y------  741
XP_341877      704   M--S---R---E--.--.DGIYA--A.AGL------RQV.VKWT...A.-N--Y------  734
XP_001498813   489   M--S---R---E--.--.DGIYA--A..GL------RQV.VKWT...A.-N--Y------  519
ABE86676       1054  L--SRI-.---R--N--T----L--V..GV------R..LPW-....-.-L--D------
1080
XP_851836      702   M--S---R---E--.--.DGIYA--A..GL------RQV.VKWT...A.-N--Y------  732
XP_001169294   576   M--S---R---E--.--.DGVYA--A..GL------RQV.VKWT...A.-N--Y------  606

1_27736        245   --G---R---YD---NSVDTYAFGILFW-YICA-GHV---KLPYV-FEQCQTKDQLWSCV  287
AAV40860       816   --.---K---..---....V........-.L..-.S.---...EA-..K.SS.....NN.  858
AAH67573       411   --.---K---..---....V........-.L.S-.S.---...EA-..K.AS.....TN.  453
Q4VSN2         816   --.---K---..---....V........-.L..-.S.---...EA-..K.SS.....NN.  858
Q4VSN1         785   --.---K---..---....V........-.L.S-.S.---...EA-..K.AS.....TN.  827
AAP42423       757   --.---K---..---....V........-.L.S-.S.---...EA-..K.AS.....TN.  799
NP_991190      757   --.---K---..---....V........-.L.S-.S.---...EA-..K.AS.....TN.  799
NP_001020439   825   --.---K---..---....V........-...S-.S.---...EA-..R.AS..H..NN.  867
ABD72471       801   --.---K---..---....V........-...S-.S.---...EA-..R.AS..H..NN.  843
NP_001035038   825   --.---K---..---....V........-...S-.S.---...EA-..R.AS..H..NN.  867
NP_056190      823   --.---K---..---....V........-...S-.S.---...EA-..R.AS..H..NN.  865
NP_001029338   824   --.---K---..---....V........-...S-.S.---...EA-..R.AS..H..NN.  866
Q6XUX3         823   --.---K---..---....V........-...S-.S.---...EA-..R.AS..H..NN.  865
```

```
AAH72406       823   --.---K---.----....V..........-...S-.S.-----...EA-..R.AS..H..NN.  865
NP_001091920   837   --.---K---.----...........L.-.V..-.....-----...QA-....AN..H..TS.  879
EDM09796       723   --.---K---.----....V..........-...S-.SI----...EA-..R.AS..H..NN.  765
BAD32232       552   --.---K---.----....V..........-...S-.SI----...EA-..R.AS..H..NN.  594
BAE26444       799   --.---K---.----....V..........-...S-.SI----...EA-..R.AS..H..NN.  841
NP_955750      821   --.---K---.----....V..........-...S-.SI----...EA-..R.AS..H..NN.  863
NP_766104      821   --.---K---.----....V..........-...S-.SI----...EA-..R.AS..H..NN.  863
NP_989837      824   --.---K---.----....V..........-...S-..------...EA-..R.AS..H..NN.  866
EDL39678       821   --.---K---.----....V..........-...S-.SI----...EA-..R.AS..H..NN.  863
ABK42490       408   --.---K---.----....V..........-...S-.SI----...EA-..R.AS..H..NN.  450
ABK42489       408   --.---K---.----....V..........-...S-.SI----...EA-..R.AS..H..NN.  450
ABD77593       798   --.---K---.----....V.........-.L.T-.S.-----...EA-..R.SS.....TN.  840
AAH89380       344   --.---K---.----....V..........-...S-.SI----...EA-..R.AS..H..NN.  386
XP_001489301   810   --.---K---.----....V..........-...S-.S.-----...EA-..R.AS..H..NN.  852
NP_001019995   822   --.---K---.----....V..........-...S-.S.-----...EA-..R.AS..H..NN.  864
BAA32317       259   --.---K---.----....V..........-...S-.S.-----...EA-..R.AS..H..NN.  301
BAC34316       284   --.---K---.----....V..........-...S-.SI----...EA-..R.AS..H..NN.  326
AAH97571       812   --.---K---.----....V..........-...S-.S.-----...EA-..K.AS..H..NN.  854
NP_001007477   814   --.---K---.----....V..........-...S-.S.-----...EA-..K.AS..H..NN.  856
NP_001087088   812   --.---K---.----....V..........-...S-.S.-----...EA-..K.AS..H..NN.  854
AAY81956       768   --.---H---.---S...V.........-.L..-.....---R...T-...FHN.EL..TS.  810
NP_001071281   806   --.---H---.---S...V.........-.L..-.....---R...T-...FHN.EL..TS.  848
NP_001027827   788   ------------------------------------------------------------  787
NP_955749      823   ------------------------------------------------------------  822
AAH53627       823   ------------------------------------------------------------  822
CAG06841       904   --.---K---.----....V.........-.L..-.S.----...EA-..K.SS.....NN.  946
CAF88742       518   --.---K---.----....V.........-.L..-.S.----...EA-..K.SS.....NN.  560
XP_969042      634   --.---.---.---S...V..........-.....-.Q.----...NH-.D.F.N.E...NS.  676
AAH48204       162   ------------------------------------------------------------  161
ABG54351       194   --.---K---SNMVSEKI.V.S...VM.-ELLT-.-----EE..A-DMH.ASI--IGGI.  235
NP_200569      962   --.---K---SNMVSEKI.V.S...VM.-ELLT-.-----EE..A-DMH.ASI--IGGI.
1003
EAZ03683       998   --.SSNK---VS---EK..VFS...VM.-E.LT-.-----EE..A---NMHYGAIIGGI.
1039
XP_001308850   384   PGA---Q---.---TK..V...A.VM.-EALV-KEI-----..HGM.PM.IVA.VMMND  427
EAZ39614       1077  --.SSNK---VS---EK..VFS...VM.-E.LT-.-----EE..A---NMHYGAIIGGI.
1118
AAU87044       227   --.SSNK---VS---EK..VFS...VM.-E.LT-.-----EE..A---NMHYGAIIGGI.  268
XP_640615      504   --Q---S---.S---TA..VFS...VL                                    519
EAY83740       4209  --.SSS.---VS---EK..VFS...AL.-E.LT-.-----EE..A-NMH.GAI--IGGI.
4250
EAZ21079       4253  --.SSS.---VS---EK..VFS...AL.-E.LT-.-----EE..A-NMH.GAI--IGGI.
4294
XP_001320204   342   --P---F---.---ER..V.S...FL.-ELLT-.-----QM..---KDM.ANQIIRTVT  380
ABG54350       194   --.SSSK---VS---EK..VFS...VL.-E.LT-.-----EE..A---NMHYGAIIGGI.  235
XP_695118      739   --.---.---.T---TES.VWS..V.L.-ETFS-RG.TPYTI.T.-MSNQ..R.EVERGY  784
NP_001067178   1000  --.SSS.---VS---EK..VFS...AL.-E.LT-.-----EE..A-NMH.GAI--IGGI.
1041
```

```
XP_001309559  392   --K---.---.G---PK..S.S...VL.-EMET-.LI---P-----.REKTH.EIIDHV.  430
ABK06418      194   --.SSSK---VS---EK..VFS...VL.-E.LT-.-----EE..A---NMHYGAIIGGI.  235
CAN82462      1299  --.---SSSLVS---EK..VFS...VM.-ELLT-.-----EE..A---DLHYGAIIGGI.
1340
XP_001327096  376   --N---QSS-.N---HMI.V.SY..VL.-E.T.-QA.-----.---RDLDSPQIIAKV.  416
NP_181050     1154  --.SSSK---VS---EK..VFS...VL.-E.LT-.-----EE..A---NMHYGAIIGGI.
1195
AAN33122      733   --.---.---.S---SES.VWS....L.-ETFSL.AS---PY.NL-TN.-..RE----F.  771
CAN65102      1107  --.SSNK---VS---EK..VFS...VL.-E.LT-.-----EE..A---NMHYGAIIGGI.
1148
NP_034324     735   --.---.---.S---SES.VWS....L.-ETFSL.AS---PY.NL-TN.-..RE----F.  773
EDL06992      742   --.---.---.S---SES.VWS....L.-ETFSL.AS---PY.NL-TN.-..RE----F.  780
XP_341877     735   --.---.---.S---SES.VWS....L.-ETFSL.AS---PY.NL-TN.-..RE----F.  773
XP_001498813  520   --.---.---.S---SES.VWS....L.EAFSL-.AS---PY.NL-SN.-..RE----F.  558
ABE86676      1081  --.NSS.---VS---EK..IFS...TM.-E.LT-.-----EE..A-NMH.GAI--IGGI.
1122
XP_851836     733   --.---.---.S---SES.VWS....L.EAFSL-.AS---PY.NL-SN.-..RE----F.  771
XP_001169294  607   --.---.---.S---SES.VWS....L.-ETFSL.AS---PY.NL-SN.-..RE----F.  645

1_27736       288   RK-----GAR---PERL-PQFTD-DCW-R-LMEQCWASDPQ  316
AAV40860      859   ..-----..----....-.C.DE-E..-Q-...A..NG..   886
AAH67573      454   K.-----.C.---....-.V.DE-E..-Q-...A..NG..   481
Q4VSN2        859   K.-----..----....-.C.DE-E..-Q-...A..NG..   886
Q4VSN1        828   K.-----.C.---....-.V.DE-E..-Q-...A..NG..   855
AAP42423      800   K.-----.C.---....-.V.DE-E..-Q-...A..NG..   827
NP_991190     800   K.-----.C.---....-.V.DE-E..-Q-...A..NG..   827
NP_001020439  868   .R-----..----....-.V.DE-E..-Q-...A..DG..   895
ABD72471      844   .R-----..----....-.V.DE-E..-Q-...A..DG..   871
NP_001035038  868   .R-----..----....-.V.DE-E..-Q-...A..DG..   895
NP_056190     866   .R-----..----....-.V.DE-E..-Q-...A..DG..   893
NP_001029338  867   .R-----..----....-.V.DE-E..-Q-...A..DG..   894
Q6XUX3        866   .R-----..----....-.V.DE-E..-Q-...A..DG..   893
AAH72406      866   .R-----..----....-.V.DE-E..-Q-...A..DG..   893
NP_001091920  880   K.-----.V.---....R...D.-AS.-N-..KSS..GE.   908
EDM09796      766   .R-----.T.---....-.V.DE-E..-Q-...A..DG..   793
BAD32232      595   .R-----.T.---....-.V.DE-E..-Q-...A..DG..   622
BAE26444      842   .R-----.T.---....-.V.DE-E..-Q-...A..DG..   869
NP_955750     864   .R-----.T.---....-.V.DE-E..-Q-...A..DG..   891
NP_766104     864   .R-----.T.---....-.V.DE-E..-Q-...A..DG..   891
NP_989837     867   .R-----.V.---....-.V.DE-E..-Q-...A..DG.    893
EDL39678      864   .R-----.T.---....-.V.DE-E..-Q-...A..DG..   891
ABK42490      451   .R-----.T.---....-.V.DE-E..-Q-...A..DG..   478
ABK42489      451   .R-----.T.---....-.V.DE-E..-Q-...A..DG..   478
ABD77593      841   K.-----.S.---....-AS.DE-E..-Q-...A..NG..   868
AAH89380      387   .R-----.T.---....-.V.DE-E..-Q-...A..DG..   414
XP_001489301  853   .R-----..----....-.V.DE-E..-Q-...A..DG..   880
NP_001019995  865   .R-----..----....-.V.DE-E..-Q-...A..DG..   892
BAA32317      302   .R-----..----....-.V.DE-E..-Q-...A..DG..   329
```

BAC34316	327	.R-----.T.-------.-.V.DE-E..-Q-...A..DG..	354
AAH97571	855	..-----..-------...-TI.DE-E..-K-...A..NG..	882
NP_001007477	857	..-----..-------...-AI.DE-E..-K-...A..NG..	884
NP_001087088	855	..-----..-------...-TI.DE-E..-K-...A..NG..	882
AAY81956	811	K.ALMIV.I.-------.-.S.D.-E..-.-.......SGE.	843
NP_001071281	849	K.ALMIV.I.-------.-.S.D.-E..-.-.......SGE.	881
NP_001027827	788	-------..-------...-.C.DE-E..-Q-...A..NG..	813
NP_955749	823	-------..-------...-.V.DE-E..-Q-...A..DG..	848
AAH53627	823	-------..-------...-.V.DE-E..-Q-...A..DG..	848
CAG06841	947	..------.EA---TL..	955
CAF88742	561	..------.EA---TL..	569
XP_969042	677	..------.I.----..C.-AH.NQ-A..-N-.......AE.	704
AAH48204	162	-------..-------...-.V.DE-E..-Q-...A..DG..	187
ABG54351	236	NN-----AL.----.-KI-..WC.-PE.-KG...S..T.E.	263
NP_200569	1004	NN-----AL.----.-KI-..WC.-PE.-KG...S..T.E.	1031
EAZ03683	1040	NN-----TL.----.P-V-.ASC.-PE.R.-.......P..	1067
XP_001308850	428	LR-----PHI---.KDT-.PAFE-.------..K....RN.	452
EAZ39614	1119	NN-----TL.----.P-V-.ASC.-PE.R.-.......P..	1146
AAU87044	269	NN-----TL.----.H-V-.ASC.-PE.R.-.......P..	296
EAY83740	4251	NN-----TL.----.P-I-.KNCE-PE.-.Q......SA..	4278
EAZ21079	4295	NN-----TL.----.P-I-.KNCE-PE.-.Q......SA..	4322
XP_001320204	381	EL-----.E.PPI..DC-..HLA-----K-.ITK..SQ..E	409
ABG54350	236	NN-----TL.----.T-I-.G.C.-.E.-.T...E...PN.	263
XP_695118	785	.M-----P.-----.NNC-.----.-EIY-T-..C...QY..R	809
NP_001067178	1042	NN-----TL.----.P-I-.KNCE-PE.-.Q......SA..	1069
XP_001309559	431	NR-----.W.---LPLS-RTVP.-SLR-.-.ITR..SEN.	458
ABK06418	236	NN-----TL.----.T-V-.NYC.-PE.-.M.......P..	263
CAN82462	1341	SN-----TL.----.S-V-.E.C.-PE.-.A...R..S.E.	1368
XP_001327096	417	SS-----DF.----.P-I-.EG.HP.IV-N-.IK...DR..	444
NP_181050	1196	NN-----TL.----.T-V-.NYC.-PE.-.M.......P..	1223
AAN33122	772	E.------.H.LPC..LC-.----.-AVF-.-.......YE.	799
CAN65102	1149	SN-----TL.----.T-V-.SSC.-PE.-.T.......PN.	1176
NP_034324	774	E.------.H.LPC..LC-.----.-AVF-.-.......YE.	801
EDL06992	781	E.------.H.LPC..LC-.----.-AVF-.-.......YE.	808
XP_341877	774	E.------.H.LPC..LC-.----.-AVF-.-.......YE.	801
XP_001498813	559	E.------.G.LPC..LC-.----.-AVF-.-.......YE.	586
ABE86676	1123	SN-----TL.----.S-I-.KRC.-SE.K.-...E..SP..E	1151
XP_851836	772	E.------.G.LPC..LC-.----.-AVF-.-.......YE.	799
XP_001169294	646	E.------.G.LPC..LC-.----.-AVF-.-.......YE.	673
