# Supplementary figures and images for: Global comparative analysis of ESTs from the southern cattle tick, Rhipicephalus (Boophilus) microplus
Source: BMC Genomics. 2007 Oct 12;8:368. doi: 10.1186/1471-2164-8-368 (PMC2100071; doi:10.1186/1471-2164-8-368)

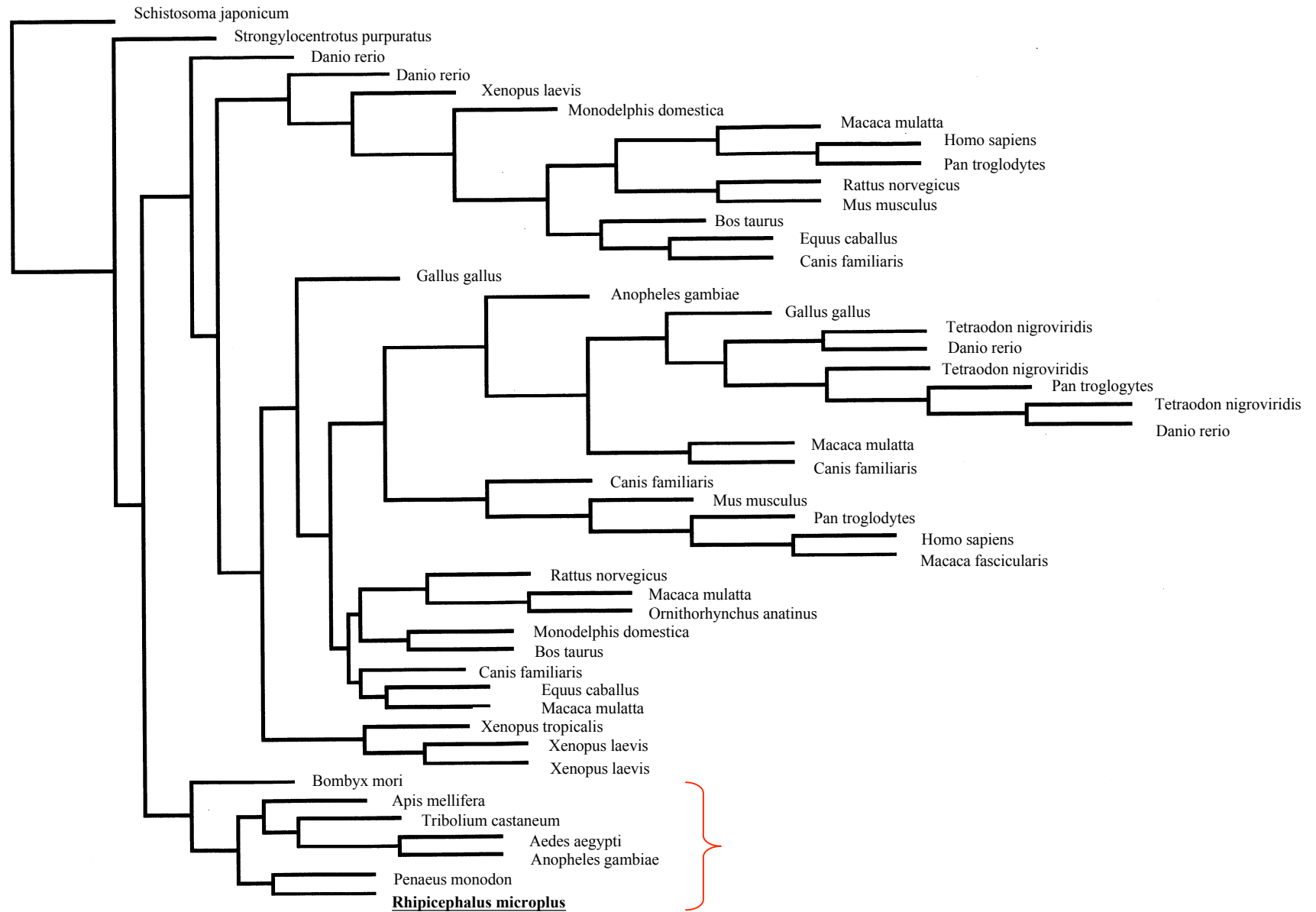

Supplement: Additional file 3 — Phylogenetic tree of TC9268. The data provided shows the entire tree generated from the TC9268 alignment [file 1471-2164-8-368-S3.pdf]

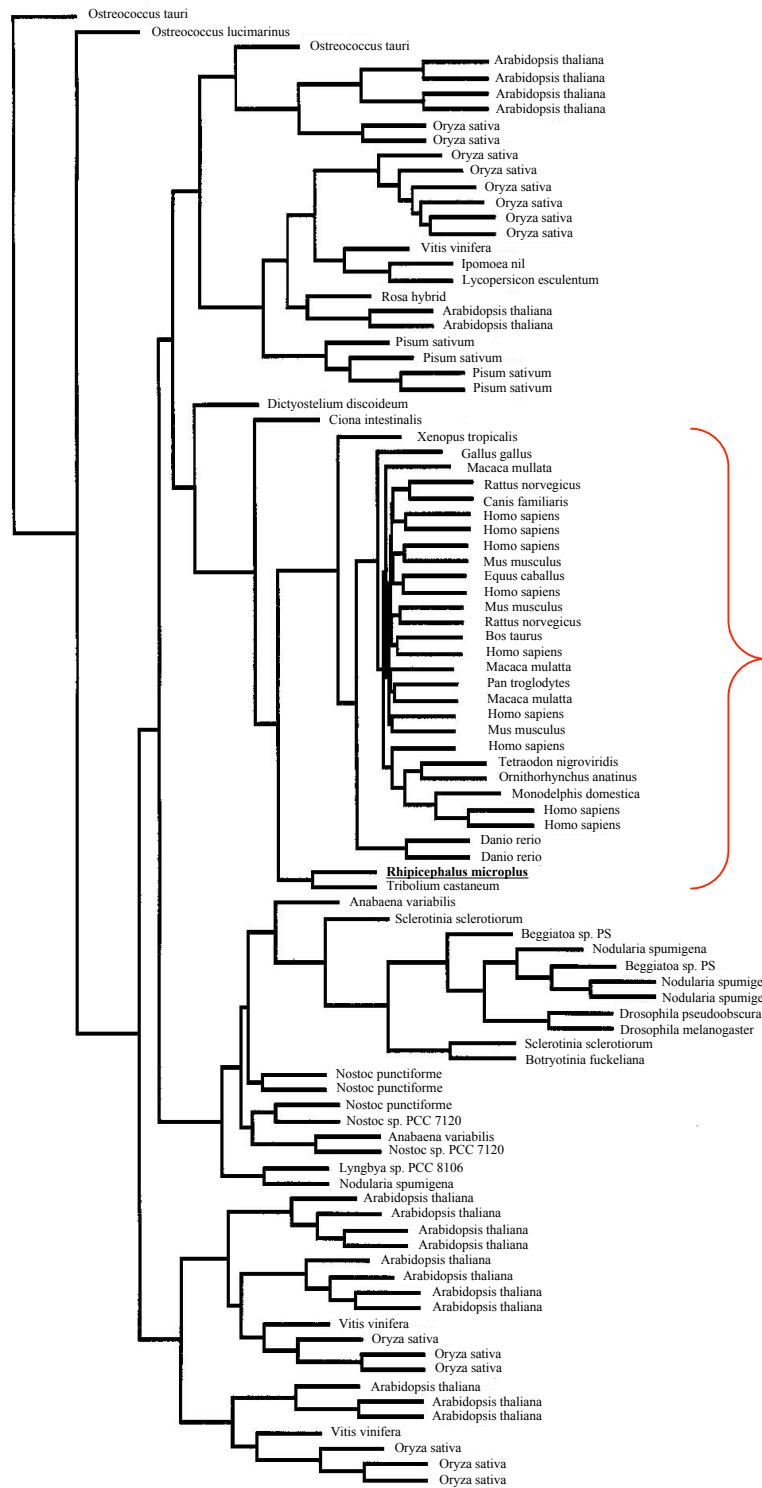

Supplement: Additional file 5 — Phylogenetic tree of TC13445. The data provided shows the entire tree generated from the TC13445 alignment [file 1471-2164-8-368-S5.pdf]

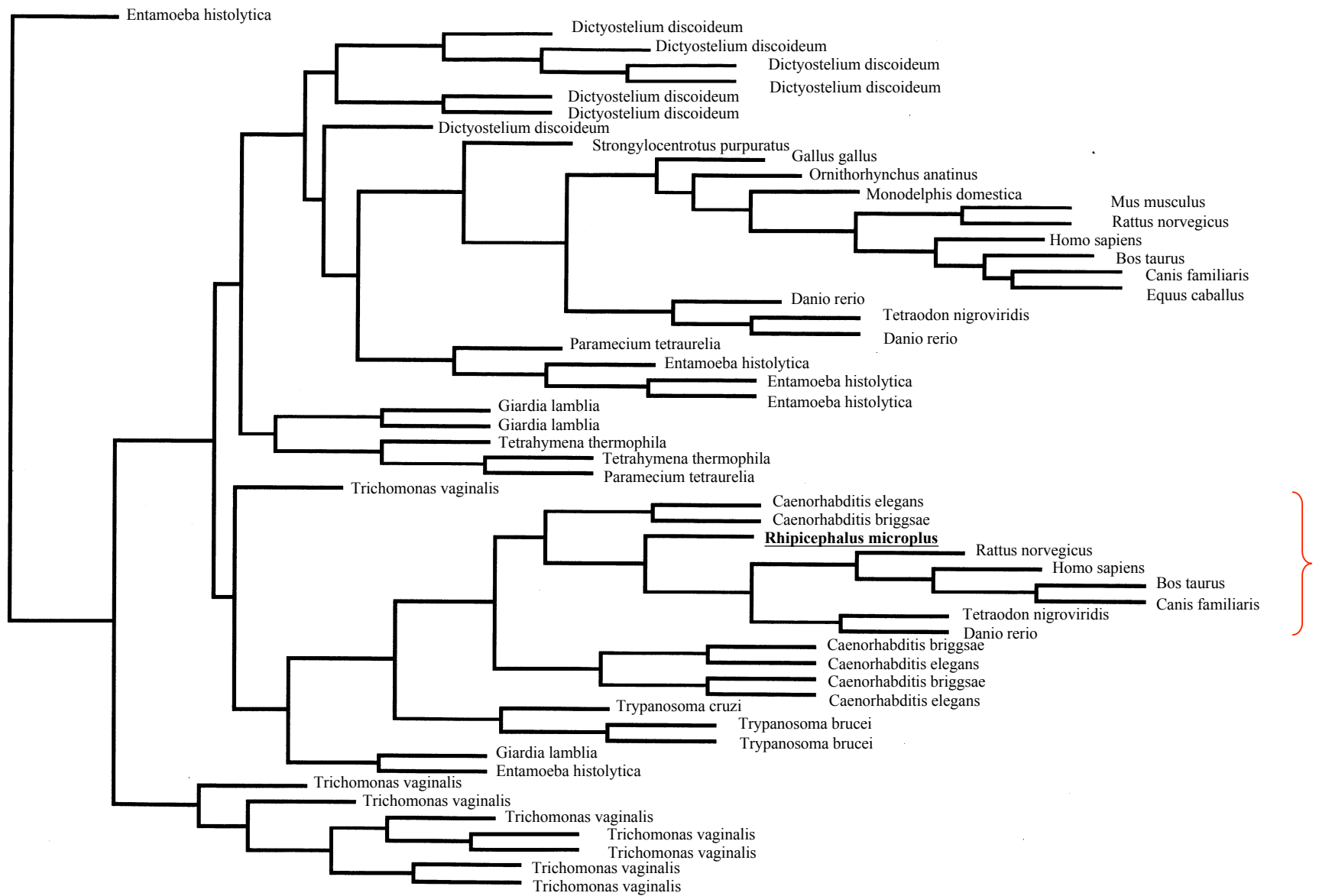

Supplement: Additional file 7 — Phylogenetic tree of TC12600. The data provided shows the entire tree generated from the TC12600 alignment [file 1471-2164-8-368-S7.pdf]
