# Supplementary material for: Global comparative analysis of ESTs from the southern cattle tick, Rhipicephalus (Boophilus) microplus
Source: BMC Genomics. 2007 Oct 12;8:368. doi: 10.1186/1471-2164-8-368 (PMC2100071; doi:10.1186/1471-2164-8-368)
Supplement: Additional file 4 — Alignment from Clustal W for TC9268. This file shows the alignment which was used to generate the phylogenetic tree for TC9268 [file 1471-2164-8-368-S4.pdf]

Alignment view for rid: 8JUPJJDX012, query ID: lcl|1_2702, database: nr

Mouse over the sequence identifer for sequence title

```
1_2702        1    SNMVAPISGSSVGLKRAHVSHGIREVILCKDKKGKVGLRVQAINQGIFVVLVQANSPAAM  60
AAK13497      26   .G.......A.P..Y..Q.TNS...IT....SE....M..K...K.V..C..KK.....L  85
NP_001080412  87   N.....V...D..IR..EIKQ..........QD..I...LKS.DN....Q...V....SL  146
BAE31671      93     ....VT.NDA.IR..EIKQ..........QD..I...LKS.DN....Q........SL  150
XP_972815     73   TS.I..L..Q.L..H..Q.TN.V.QLT.....D.......K...N.V..C..VD.....L  132
NP_001006801  87   N.....V..ND..IR..EIKQ....A.....QD..I...LKS.DN....Q...T....SL  146
NP_001087618  87   N.....V..ND..IR..EIKQ..........QD..I...LKS.DN....Q...G....SL  146
EDL05687      123    ....VT.NDA.IR..EIKQ..........QD..I...LKS.DN....Q........SL  180
BAE30020      93     ....VT.NDA.IR..EIKQ..........QD..I...LKS.DN....Q........SL  150
BAE29785      93     ....VT.NDA.IR..EIKQ..........QD..I...LKS.DN....Q........SL  150
BAE41161      93     ....VT.NDA.IR..EIKQ..........QD..I...LKS.DN....Q........SL  150
BAE31729      93     ....VT.NDA.IR..EIKQ..........QD..I...LKS.DN....Q........SL  150
BAE29647      93     ....VT.NDA.IR..EIKQ..........QD..I...LKS.DN....Q........SL  150
NP_001091697  93     ....VT.NDA.IR..EIKQ..........QD..I...LKS.DN....Q........SL  150
BAE29746      92     ....VT.NDA.IR..EIKQ..........QD..I...LKS.DN....Q........SL  149
NP_058087     92     ....VT.NDA.IR..EIKQ..........QD..I...LKS.DN....Q........SL  149
NP_001068951  90   NH.....T.GDI.IR..EIKQ..........QD..I...LKS.DN....Q.......SSL  149
NP_114192     94     ....VT.NDA.IR..EIKQ..........QD..I...LKSVDN....Q........SL  151
ABQ12975      92     .....T.GDI.IR..EIKQ..........QD..I...LKS.DN....Q.......SSL  149
XP_001088209  86     ....VT.ND..IR..EIKQ..........QD..I...LKS.DN....Q........SL  143
NP_001007069  86     ....VT.ND..IR..EIKQ..........QD..I...LKS.DN....Q........SL  143
XP_624043     91     II..L..Q.L..Q..Q.TN....L......D..I....H.V.N....C..SQ.....L  148
BAE34989      92     ....VT.NDA.IR..EIKQ..........QD..I...LKS.DN....Q........SL  149
XP_001496922  87     ....VT.ND..IR..EIKQ..........QD..I...LKS.DN.V..Q........SL  144
XP_001088096  98     ....VT.ND..IR..EIKQ..........QD..I...LKS.DN....Q........SL  155
XP_001087973  91     ....VT.ND..IR..EIKQ..........QD..I...LKS.DN....Q........SL  148
NP_001007070  91     ....VT.ND..IR..EIKQ..........QD..I...LKS.DN....Q........SL  148
NP_001007068  92     ....VT.ND..IR..EIKQ..........QD..I...LKS.DN....Q........SL  149
AAB51246      92     ....VT.ND..IR..EIKQ..........QD..I...LKS.DN....Q........SL  149
AAB97144      92     ....VT.ND..IR..EIKQ..........QD..I...LKS.DN....Q........SL  149
EAW86812      112    ....VT.ND..IR..EIKQ..........QD..I...LKS.DN....Q........SL  169
AAC27646      92     ....VT.NDA.IR..EIKQ..........QD..I...LKS.DN....Q........SL  149
BAE31548      93     ....VT.NDA.IR..EIKQ..........QD..I...LKS.DN....Q........SL  150
XP_001496903  93     ....VT.ND..IR..EIKQ..........QD..I...LKS.DN.V..Q........SL  150
BAE35165      92     ....VT.NDA.IR..EIKQ.H........QD..I...LKS.DN....Q........SL  149
XP_540090     91     ....VT.ND..IR..EIKQ..........QD..I...LKS.DN....Q........SL  148
CAH92473      92     ....VT.NDA.IR..EIKQ..........QD..I...LKS.DN....Q........SL  149
CAH91518      91     ....VT.NDA.IR..EIKQ..........QD..I...LKS.DN....Q........SL  148
NP_001026195  88     ....VT.NDI.VR..EIKQ....T.....QD..I...LKSVDN....Q........SL  145
EDM11636      36     ....VT.NDA.IR..EIKQ..........QD..I...LKSVDN....Q........SL  93
XP_001379494  91     ....VT..D..IR..EIKQ..........QD..I...LKS.DN....Q........SL  148
EAT43140      123  A.....V..G....Q.GQ.TN....L....GADK.....A...HK.V..C..VK.....L  182
ABF18402      125  A.....V..G....Q.GQ.TN....L....GADK.....A...HK.V..C..VK.....L  184
XP_001157154  36     ....VT.ND..IR..EIKQ..........QD..I...LKS.DN....Q........SL  93
```

```
XP_875149      86     V...V..N.L.AL..EIKP.V..IH....ER..T...L...DK....Q.....T..SL  143
NP_001079704   84     L...VT.NDI..R..EIKN.V........QH......LR.VDK...LQ........SL  141
NP_999963      102      ...T..DL.V...EIRQ.V...V....MD..I...LK..DN.V..Q.....TA..L  157
XP_001167866   163    ....VT.Y.L.VR..EIKP.V..IH....ER..T...LRKVD..L..Q.....T..SL  220
XP_001498897   86     V...V..N.L.AR..EIKP.V..IH....ER..T...FR..D.....Q.....T..SL  143
XP_001112885   86     ....V..Y.L.VR..EIKP.V..IH....ER..T...LRTVD..L..Q.....T..SL  143
EDL05918       108    V...V..NNL.IL..EIKP.V..IH....ER..T...L..VDK.L..Q.....T..SL  165
NP_997856      93     ..R.VT.ADM.IR..EIRP.L........QE......LRD.DN.V..Q.........L  150
XP_318581      124  A.....V..HFT..Q..Q.TN....LVM..GVDK..........K....CV.VK.....L  183
CAC21716       86     ....VT.Y.L.VR..EIKP.V..IH....ER..T...LRKVD..L..Q.....T..SL  143
XP_001367355   86     ....VT.NNS.MR..EIKP.V..IH....E...M...LR..DK.L..Q.....T..SL  143
NP_663510      86     V...V..NNL.IL..EIKP.V..IH....ER..T...L..VDK.L..Q.....T..SL  143
EAX10639       75    S....VT.Y.L.VR..EIKP.V..IH....ER..T...LRKVD..L..Q.....T..SL  133
NP_536737      86     ....VT.Y.L.VR..EIKP.V..IH....ER..T...LRKVD..L..Q.....T..SL  143
XP_542935      81     L...L..N.L.TR..EIKP.V..IY....EH..T...LR..D..L..Q..K.....SL  138
XP_001112910   1      ....V..Y.L.VR..EIKP.V..IH....ER..T...LRTVD..L..Q.....T..SL  58
AAD20049       1      ....VT.Y.L.VR..EIKP.V..IH....ER..T...LRKVD..L..Q.....T..SL  58
NP_056500      1      ....VT.Y.L.VR..EIKP.V..IH....ER..T...LRKVD..L..Q.....T..SL  58
NP_001020863   90     VT..V..NDL.VL..EIKP.V..IH....ER..T...L..VDK.L..Q.....T..SL  147
NP_001040345   80   ..V...L.SQ.LS.PK.T.TQA..Q.V....RN..C...LHSVDS.V..CY.A....G.L  139
XP_857475      92     ....VT.ND..IR..EIKQ..........QD..I...LKS.DN....Q........SL  149
XP_001514333   126                                           .MWVPS....Q........SL  146
XP_001514311   132                                           .MWVPS....Q........SL  152
1OBZ-A         6                             ........QD..I...LKS.DN....Q........SL  42
XP_001087599   81                                                 ....Q........SL  95
XP_001157051   81                                                 ....Q........SL  95
CAG14594       1                                                  .L..Q.......S.L  15
1N99-A         6                             ........QD..I...LKS.DN....Q........SL  42
EAW86816       91     ....VT.ND..IR..EIKQ..........QD..I...LKS.DN....Q........SL  148
CAM27238       92     ....VT.NDA.IR..EIKQ..........QD..I...LKS.DN....Q........SL  149
EAW86813       91     ....VT.ND..IR..EIKQ..........QD..I...LKS.DN....Q........SL  148
XP_001159695   4      ..V.VTENDA.IR..EIKQ..........QD..I...LKS.DND...Q........SL  61
CAM27241       93     ....VT.NDA.IR..EIKQ..........QD..I...LKS.DN....Q........SL  150

1_2702         61   AGLRFGDQLLTINEEVLAGYSVDKVHTLIVKADPDRIVMAVRDRPFERTVTMHKSSTGHV  120
AAK13497       86   ........I.L..S.NV....ME...DMLK.SPTNG.SL.I.....G....L..D....I  145
NP_001080412   147  ...K....I.Q..G.NC..W.S..S.KMLKQVSGE..S.V.........I....D.....  206
BAE31671       151  ........V.Q..G.NC..W.S..A.KVLKQ.FGEK.T.TI.............D.S...  210
XP_972815      133  ........I.Q..GATV..FTM....EMFK.SPVNG.NVV.........L.L..D.A.T.  192
NP_001006801   147  ...K....I.Q..G.NC..W.S..S.KILKQVSGE..S.I.........I....D.....  206
NP_001087618   147  ...K....I.Q..G.NC..W.S..S.KVLKQVSGE..S.V.........I....D.....  206
EDL05687       181  V.......V.Q..G.NC..W.S..A.KVLKQ.FGEK.T.TI.............D.S...  240
BAE30020       151  V.......V.Q..G.NC..W.S..A.KVLKQ.FGEK.T.TI.............D.S...  210
BAE29785       151  V.......V.Q..G.NC..W.S..A.KVLKQ.FGEK.T.TI.............D.S...  210
BAE41161       151  V.......V.Q..G.NC..W.S..A.KVLKQ.FGEK.T.TI.............D.S...  210
BAE31729       151  V.......V.Q..G.SC..W.S..A.KVLKQ.FGEK.T.TI.............D.S...  210
BAE29647       151  V.......V.Q..G.NC..W.S..A.KVLKQ.FGEK.T.TI.............D.C...  210
```

NP_001091697	151	V.......V.Q..G.NC..W.S..A.KVLKQ.FGEK.T.TI............D.S...	210
BAE29746	150	V.......V.Q..G.NC..W.S..A.KVLKQ.FGEK.T.TI............D.S...	209
NP_058087	150	V.......V.Q..G.NC..W.S..A.KVLKQ.FGEK.T.TI............D.S...	209
NP_001068951	150	V.......V.Q..G.NC..W.S.RA.KVLKQ.FGEK.T.TI.......I...D.....	209
NP_114192	152	V.......V.Q..G.NC..W.S..A.KVLKQ.FGEK.T.TI............D.S...	211
ABQ12975	150	V.......V.Q..G.NC..W.S.RA.KVLKQ.FGEK.T.TI.......I...D.....	209
XP_001088209	144	V.......V.Q..G.NC..W.S..A.KVLKQ.FGEK.T.TI.......I...D.....	203
NP_001007069	144	V.......V.Q..G.NC..W.S..A.KVLKQ.FGEK.T.TI.......I...D.....	203
XP_624043	149	........I.S..DVFV...TMEQ..KMLRN..ING.KVV.............D....I	208
BAE34989	150	V.......V.QT.G.NC..W.S..A.KVLKQ.FGEK.T.TI............D.S...	209
XP_001496922	145	V.......V.Q..G.NC..W.S..S.KVLKQ.FGEK.T.TI.......I...D.....	204
XP_001088096	156	V.......V.Q..G.NC..W.S..A.KVLKQ.FGEK.T.TI.......I...D.....	215
XP_001087973	149	V.......V.Q..G.NC..W.S..A.KVLKQ.FGEK.T.TI.......I...D.....	208
NP_001007070	149	V.......V.Q..G.NC..W.S..A.KVLKQ.FGEK.T.TI.......I...D.....	208
NP_001007068	150	V.......V.Q..G.NC..W.S..A.KVLKQ.FGEK.T.TI.......I...D.....	209
AAB51246	150	V.......V.Q..G.NC..W.S..A.KVLKQ.FGEK.T.TI.......I...D.....	209
AAB97144	150	V.......V.Q..G.NC..W.S..A.KVLKQ.FGEK.T.TI.......I...D.....	209
EAW86812	170	V.......V.Q..G.NC..W.S..A.KVLKQ.FGEK.T.TI.......I...D.....	229
AAC27646	150	V.......V.Q..G.NC..W.S..A.KVLKQ.FGEK.T.TI........I..D.S...	209
BAE31548	151	V.......V.Q..G.NC..W.S..A.KVLKQ.FGEK.T.TI............D.S...	210
XP_001496903	151	V.......V.Q..G.NC..W.S..S.KVLKQ.FGEK.T.TI.......I...D.....	210
BAE35165	150	V.......V.Q..G.NC..W.S..A.KVLKQ.FGEK.T.TI............D.S...	209
XP_540090	149	V.......V.Q..G.NC..W.S..A.KVLKQ.FGEK.T.T.............D.....	208
CAH92473	150	V.......V.Q..G.NC..W.S..A.KVLKQ.FGEK.T.TI.......I...D.....	209
CAH91518	149	V.......V.Q..G.NC..W.S..A.KVLKQ.FGEK.T.TI.......I...D.....	208
NP_001026195	146	........V.Q..G.NC..W.S..A.KVLKQ.SGE..S.II......II...D.....	205
EDM11636	94	V.......V.Q..G.NC..W.S..A.KVLKQ.FGEK.T.TI............D.S...	153
XP_001379494	149	V.......V.Q..G.NC..WGS..A.KVLKQ.FGEK.T.TI............D.....	208
EAT43140	183	........I.QV.GTLV..F...D..K.LK.S.KNN.SLV........A..L..D.A.T.	242
ABF18402	185	........I.QV.GTLV..F...D..K.LK.S.KNN.SLV........A..L..D.A.T.	244
XP_001157154	94	V.......V.Q..G.NC..W.S..A.KVLKQ.FGEK.T.TI.......I...D.....	153
XP_875149	144	V.......I.Q.DGRDC..W.T..A.QVVK..SAEK..II......Q..I...D.....	203
NP_001079704	142	V.......V.Q.DGDSC..W.T.RA.KALK..SQ...SLI......Q..I.LQ.D.....	201
NP_999963	158	........I.E..GKSC..WNS.YA.KVLKNSN.E..TL......L.....L..DMN.QL	217
XP_001167866	221	V.......I.Q.DGRDC..W.SH.A.QVVK..SG.K...V......Q.......D.M...	280
XP_001498897	144	V.......I.Q.DGRDC..W.T..A.RVVK..SAEK...V......Q.NI.L..D.M...	203
XP_001112885	144	V.......I.Q.DGRDC..W.SR.ANQVVK..S.EK...V......Q.......D.M...	203
EDL05918	166	V.......I.Q.DGCDC..WNTH.A.KVLK..SAEK...VI.....Q.......D.S.Q.	225
NP_997856	151	........V.Q..GQNV..WNS..A.KALKA.AEQ..ELI......Q..I....D.S...	210
XP_318581	184	........I.Q..GTLV..F...D..K.LK.S.KNN.SVV........AI.L..D.A.SF	243
CAC21716	144	V.........Q.DGRDC..W.SH.A.QVVK..SG.K...V......Q.......D.M...	203
XP_001367355	144	V.......I.Q.DGRDC..W.T..ARKVMK..SHEK...I......Q....L..D.N.C.	203
NP_663510	144	V.......I.Q.DGCDC..WNTH.A.KVLK..SAEK...VI.....Q.......D.S.Q.	203
EAX10639	134	V.........Q.DGRDC..W.SH.A.QVVK..SG.K..VV......Q.......D.M...	193
NP_536737	144	V.........Q.DGRDC..W.SH.A.QVVK..SG.K..VV......Q.......D.M...	203
XP_542935	139	V.......I.Q.DGRDC..W.T.RA.RVLKR.SAEK.A.V......Q.......D.....	198
XP_001112910	59	V.......I.Q.DGRDC..W.SR.ANQVVK..S.EK...V......Q.......D.M...	118

```
AAD20049       59   V.........Q.DGRDC..W.SH.A.QVVK..SG.K...V......Q.......D.M...  118
NP_056500      59   V.........Q.DGRDC..W.SH.A.QVVK..SG.K..VV......Q.......D.M...  118
NP_001020863   148  V.......I.Q.DGCDC..W.TH.AQKALK..SAEK...V......Q.......D.S.Q.  207
NP_001040345   140  ........I.E..NVTV..MTM..C.DILK..PANN.T..........N..L..D.L...  199
XP_857475      150  V.......V.Q..G.NC..W.S..A.KVLKQ.FGEK.T.T..............D.....  209
XP_857475      113                                                 .E.ILC.DQD.KI  125
XP_001514333   147  V.M.....V.Q..G.NC..W.S..S.KVLKQ.FGEK.T.TI........I....D.....  206
XP_001514311   153  V.M.....V.Q..G.NC..W.S..S.KVLKQ.FGEK.T.TI........I....D.....  212
1OBZ-A         43   V.......V.Q..G.NC..W.S..A.KVLKQ.FGEK.T.TI........I....D.....  102
XP_001087599   96   V.......V.Q..G.NC..W.S..A.KVLKQ.FGEK.T.TI........I....D.....  155
XP_001157051   96   V.......V.Q..G.NC..W.S..A.KVLKQ.FGEK.T.TI........I....D.....  155
CAG14594       16   ........V.Q..GQNC..W.T..A.KALKA.GES..ELV......Q.......D.....  75
1N99-A         43   V.......V.Q..G.NC..W.S..A.KVLKQ.FGEK.TXTI........I.X..D.....  102
EAW86816       149  V.......V.Q..G.NC..W.S..A.KVLKQ.FGEK.T.TI........I....D.....  208
EAW86816       112                                                 .E.ILC.DQD.KI  124
CAM27238       150  V.......V.Q..G.NC..W.S..A.KVLKQ.FGEK.T.TI.............D.S...  209
CAM27238       113                                                 .E.ILC.DQD.KI  125
EAW86813       149  V.......V.Q..G.NC..W.S..A.KVLKQ.FGEK.T.TI........I....D...     206
EAW86813       112                                                 .E.ILC.DQD.KI  124
CAG14595       88                                              ...Q.......D.....  104
EDM11637       1                                          .TI.............D.S...  22
XP_001492473   96                                       L.LTIH.......I....D..VR.  119
XP_001159695   62   V.......V.Q.-----S.FWREDY.D-----------.TI........I.K..D.....  105
XP_001477912   17                                              ...........D.S...  33
CAM27241       151  V.......V.Q..G.NC..W.S..A.KVLKQ.FGEK.T.TI...                  194
CAM27241       114                                                 .E.ILC.DQD.KI  126

1_2702         121  GFAFR---DGRIISLVK-DSSATRNGLLVDHHLLEVNGQNVVGIKDSEITKIIEAA-GDI  175
AAK13497       146  ..Q..---..E.TA...-....A.....T..............L..K.VSA..NEC-.QV  200
NP_001080412   207  ..I.K---N.K.T.I..-....A.....T..N.C.I.....I.L...QVAE.LATS-ANV  261
BAE31671       211  ..I.K---S.K.T.I..-....A.....T...IC.I.....I.L..AQ.AD.LST.-.TV  265
XP_972815      193  ..Q.K---N.K.TTII.-....A...V.T..Q....E......L..KQ.S.L..E.-.EV  247
NP_001006801   207  ..I.K---N.K.T.I..-....A.....T..N.C.I.....I.L...QVAELLATS-ANV  261
NP_001087618   207  ..I.K---N.K.T.I..-....A.....TE.N.C.I.....I.L...QVAE.LATS-VNV  261
EDL05687       241  ..I.K---S.K.T.I..-....A.....T...IC.I.....I.L..AQ.AD.LST.-.TV  295
BAE30020       211  ..I.K---S.K.T.I..-....A.....T...IC.I.....I.L..AQ.AN.LST.-.TV  265
BAE29785       211  ..I.K---S.K.T.I..-....A.....T...IC.I.....I.L..AQ.AD.LST.-.TV  265
BAE41161       211  ..I.K---S.K.T.I..-....A.....T...IC.I.....I.L..AQ.AD.LST.-.TV  265
BAE31729       211  ..I.K---S.K.T.I..-....A.....T...IC.I.....I.L..AQ.AD.LST.-.TV  265
BAE29647       211  ..I.K---S.K.T.I..-....A.....T...IC.I.....I.L..AQ.AD.LST.-.TV  265
NP_001091697   211  ..I.K---S.K.T.I..-....A.....T...IC.I.....I.L..AQ.AD.LST.-.TV  265
BAE29746       210  ..I.K---S.K.T.I..-....A.....T...IC.I.....I.L..AQ.AD.LST.-.TV  264
NP_058087      210  ..I.K---S.K.T.I..-....A.....T...IC.I.....I.L..AQ.AD.LST.-.TV  264
NP_001068951   210  ..V.K---N.K.T.I..-....A.....TE.NIC.......I.L...Q.AD.LST.-.N.  264
NP_114192      212  ..I.K---S.K.T.I..-....A.....T...IC.I.....I.L..AQ.AD.LST.-.TV  266
ABQ12975       210  ..V.K---N.K.T.I..-....A.....TE.NIC.......I.L...Q.AD.LST.-.N.  264
XP_001088209   204  ..I.K---N.K.T.I..-....A.....TE.NIC.I.....I.L...Q.AD.LSTS-.TV  258
```

```
NP_001007069   204  ..I.K---N.K.T.I..-....A.....TE.NIC.I.....I.L...Q.AD.LSTS-.TV  258
XP_624043      209  ..Q.K---N.K..A...-....A.....T..QI.....K..I.LM.K...AE..NG-.N.  263
BAE34989       210  ..I.K---S.K.T.I..-....A.....T...IC.I.....I.L..AQ.AD.LST.-.TV  264
XP_001496922   205  ..I.K---N.K.T.I..-....A.....TE.NIC.I.....I.L...Q.AD.LSTS-.TV  259
XP_001088096   216  ..I.K---N.K.T.I..-....A.....TE.NIC.I.....I.L...Q.AD.LSTS-.TV  270
XP_001087973   209  ..I.K---N.K.T.I..-....A.....TE.NIC.I.....I.L...Q.AD.LSTS-.TV  263
NP_001007070   209  ..I.K---N.K.T.I..-....A.....TE.NIC.I.....I.L...Q.AD.LSTS-.TV  263
NP_001007068   210  ..I.K---N.K.T.I..-....A.....TE.NIC.I.....I.L...Q.AD.LSTS-.TV  264
AAB51246       210  ..I.K---N.K.T.I..-....A.....TE.NIC.I.....I.L...Q.AD.LSTS-.TV  264
AAB97144       210  ..I.K---N.K.T.I..-....A.....TE.NIC.I.....I.L...Q.AD.LSTS-.TV  264
EAW86812       230  ..I.K---N.K.T.I..-....A.....TE.NIC.I.....I.L...Q.AD.LSTS-.TV  284
AAC27646       210  ..I.K---S.K.T.I..-....A.....T...IC.I.....I.L..AQ.AD.LST.-.TV  264
BAE31548       211  ..I.K---S.K.T.I..-....A.....T...IC.I.....I.L..AQ.AD.LST.-.TV  265
XP_001496903   211  ..I.K---N.K.T.I..-....A.....TE.NIC.I.....I.L...Q.AD.LSTS-.TV  265
BAE35165       210  ..I.K---S.K.T.I..-....A.....T...IC.I.....I.L..AQ.AD.LST.-.TV  264
XP_540090      209  ..I.K---N.K.T.I..-....A.....TE.NIC.......I.L...Q.AD.LSTS-ETV  263
CAH92473       210  ..I.K---N.K.T.I..-....A.....TE.NIC.I.....I.L...Q.AD.LSTS-.TV  264
CAH91518       209  ..I.K---N.K.T.I..-....A.....TE.NIC.I.....I.L...Q.AD.LSTS-.TV  263
NP_001026195   206  ..I.K---N.K.T.I..-....A.....TE.NIC.I.....I.L..PQ.AD.LAT.-.NV  260
EDM11636       154  ..I.K---S.K.T.I..-....A.....T...IC.I.....I.L..AQ.AD.LST.-.TV  208
XP_001379494   209  ..I..---N.K.T.I.T-....A.....TE.NIC......II.L..CQ.AD.LST.-.T.  263
EAT43140       243  ..Q.N---N.K.TAI..-....A.....IE.QM..I.....I.M..K..SQL.A.G-.Q.  297
ABF18402       245  ..Q.N---N.K.TAI..-....A.....IE.QM..I.....I.M..K..SQL.A.G-.Q.  299
XP_001157154   154  ..I.K---N.K.T.I..-....A.....TE.NIC.I.....I.L...Q.AD.LSTS-.TV  208
XP_875149      204  ..VIK---K.K......-G...A.....TN.YVC.......I.L..K.V.E.LAT.-.NV  258
NP_001079704   202  ..IYK---K.L.T....-.G..A.....TN.Y.C.......I.L..HQVGD.LASC-.RT  256
NP_999963      218  ..I..---K...T.I..-...SA.....T..QIC.I....II.L..TQ.MD.LNSS-.GV  272
XP_001167866   281  ..VIK---K.K.V....-G...A.....TN.YVC..D....I.L..KK.ME.LAT.-.NV  335
XP_001498897   204  ..VIK---K.KV..V..-G...A.....TN.SVC.......I.L..K.V.E.LAM.-.NV  258
XP_001112885   204  ..VIK---K.K.V....-G...A.....TN.YVC..D....I.L..KK.ME.LAT.-.NV  258
EDL05918       226  ..SIK---K.K.V.V..-G...A.....TN.YVC.......I.L..KKV.E.LTT.-..V  280
NP_997856      211  ..I.K---S...T....-.G..A.....TE.YIC.I.....I.L..TQ.KD.LTTS-PTA  265
XP_318581      244  ..Q.N---N.T.TAI..-....A.....I.QQ.........IAM..K..REL.SSS-DQV  298
CAC21716       204  ..VIK---K.K.V....-G...A.....TN.YVC..D....I.L..KK.ME.LAT.-.NV  258
XP_001367355   204  ..VMK---K.KV..I..-G...AQ....TN.YIC.......I.L..K.T.E.LAT.-.NV  258
NP_663510      204  ..SIK---K.K.V.V..-G...A.....TN.YVC.......I.L..KKV.E.LTT.-..V  258
EAX10639       194  ..VIK---K.K.V....-G...A.....TN.YVC..D....I.L..KK.ME.LAT.-.NV  248
NP_536737      204  ..VIK---K.K.V....-G...A.....TN.YVC..D....I.L..KK.ME.LAT.-.NV  258
XP_542935      199  ..VIK---K.AVV.V.R-G...A.....TKQSVC.........L..KAV.E.LAM.-.NV  253
XP_001112910   119  ..VIK---K.K.V....-G...A.....TN.YVC..D....I.L..KK.ME.LAT.-.NV  173
AAD20049       119  ..VIK---K.K.V....-G...A.....TN.YVC..D....I.L..KK.ME.LAT.-.NV  173
NP_056500      119  ..VIK---K.K.V....-G...A.....TN.YVC..D....I.L..KK.ME.LAT.-.NV  173
NP_001020863   208  ..FIK---K.K.V.V..-G...A.....TN.YVC.......I.L..KK.IE.LTT.-.NV  262
NP_001040345   200  ..Q.K---N.K..A..V-....A.....T..QI..I.TI....M..K..S...DES-PSV  254
XP_857475      210  ..I.K---N.K.T.I..-....A.....TE.NIC.......I.L.                250
XP_857475      126  .LRLKSIDN.IFVQ..QAN.P.SLV..RFGDQV.QI..E.CA.WSSDKAH.VLKQ.F.EK  185
XP_001514333   207  ..I.K---N.K.T.I..-....A.....TE.NIC.I.....I.L..PQ.AD.LST.-.NV  261
```

```
XP_001514311   213  ..I.K---N.K.T.I..-....A.....TE.NIC.I.....I.L..PQ.AD.LST.-.NV  267
1OBZ-A         103  ..I.K---N.K.T.I..-....A.....TE.NIC.I.....I.L...Q.AD.LSTS-.TV  157
XP_001087599   156  ..I.K---N.K.T.I..-....A.....TE.NIC.I.....I.L...Q.AD.LSTS-.TV  210
XP_001157051   156  ..I.K---N.K.T.I..-....A.....TE.NIC.I.....I.L...Q.AD.LSTS-.TV  210
CAG14594       76   ..VYK---S.K.C.....-....A.....TE.Y.C.I.....I.L...Q.KD.LSTS-PTA  130
1N99-A         103  ..I.K---N.K.T.I..-....A.....TE.NIC.I.....I.L...Q.AD.LSTS-.TV  157
EAW86816       125  .LRLKSIDN.IFVQ..QAN.P.SLV..RFGDQV.QI..E.CA.WSSDKAH.VLKQ.F.EK  184
CAM27238       126  .LRLKSIDN.IFVQ..QAN.P.SLV..RFGDQV.QI..E.CA.WSSDKAH.VLKQ.F.EK  185
EAW86813       125  .LRLKSIDN.IFVQ..QAN.P.SLV..RFGDQV.QI..E.CA.WSSDKAH.VLKQ.F.EK  184
CAG14595       105  ..VYK---S.K.C.....-....A.....TE.Y.C.I.....I.L...Q.KD.LSTS-PTA  159
EDM11637       23   ..I.K---S.K.T.I..-....A.....T...IC.I.....I.L..AQ.AD.LST.-.TV  77
XP_001492473   120  ..I.K---N.K.T.I..-....A.....TE.NIC.I.....I.L.G.Q.AD.LSTS-.TV  174
XP_001159695   106  ..I.K---N.K.GR.A.HEDKDG.K..                                  129
XP_001477912   34   ..I.K---S.K.T.IL.-....A.....T..-IC.I....II.L..AQ.AD.LST.-.TV  87
CAM27241       127  .LRLKSIDN.IFVQ..QAN.P.SLV..RFGDQV.QI..E.CA.WSSDKAH.VLKQ.F.EK  186

1_2702         176  VTVTVIPSFVYDHMIKHTSGGMLKKLMDHSIPDI  209
AAK13497       201  ....IM.....K..M..MASS-...F.......L  233
NP_001080412   262  ..L..M...IF...V.RMASSV..S.....V.EV  295
BAE31671       266  ..I.IM.T.IFE.I..RMAPSIM.S....T..EV  299
XP_972815      248  ..I..V...I....V.KMA.SL..D.....V.NL  281
NP_001006801   262  ..L..M..YIFE..V.RMASSV..N.....V.EV  295
NP_001087618   262  ..L..M..YIF...V.RMASSV..S.....V.EV  295
EDL05687       296  ..I.IM.T.IFE.I..RMAPSIM.S....T..EV  329
BAE30020       266  ..I.IM.T.IFE.I..RMAPSIM.S....T..EV  299
BAE29785       266  ..I.IM.T.IFE.I..RMAPSIM.S....T..EV  299
BAE41161       266  ..I.IM.T.IFE.I..RMAPSIM.S....T..EV  299
BAE31729       266  ..I.IM.T.IFE.I..RMAPSIM.S....T..EV  299
BAE29647       266  ..I.IM.T.IFE.I..RMAPSIM.S....T..EV  299
NP_001091697   266  ..I.IM.T.IFE.I..RMAPSIM.S....T..EV  299
BAE29746       265  ..I.IM.T.IFE.I..RMATSIM.S....T..EV  298
NP_058087      265  ..I.IM.T.IFE.I..RMAPSIM.S....T..EV  298
NP_001068951   265  ..I.IM.A.IFE.I..RMAPSIM.S....T..EV  298
NP_114192      267  ..I.IM.A.IFE.I..RMAPSIM.S....T..EV  300
ABQ12975       265  ..I.IM.A.IFE.I..RMAPSIM.S....T..EV  298
XP_001088209   259  ..I.IM.A.IFE.I..RMAPSIM.S....T..EV  292
NP_001007069   259  ..I.IM.A.IFE.I..RMAPSIM.S....T..EV  292
XP_624043      264  I.I.I...YI......KMNNSL..T.....V..V  297
BAE34989       265  ..I.IM.T.IFE.I..RMAPSIM.S....T..EV  298
XP_001496922   260  ..I.IM.A.IFE.I..RMAPSIM.S....T..EV  293
XP_001088096   271  ..I.IM.A.IFE.I..RMAPSIM.S....T..EV  304
XP_001087973   264  ..I.IM.A.IFE.I..RMAPSIM.S....T..EV  297
NP_001007070   264  ..I.IM.A.IFE.I..RMAPSIM.S....T..EV  297
NP_001007068   265  ..I.IM.A.IFE.I..RMAPSIM.S....T..EV  298
AAB51246       265  ..I.IM.A.IFE.I..RMAPSIM.S....T..EV  298
AAB97144       265  ..I.IM.A.IFE.I..RMAPSIM.S....T..EV  298
EAW86812       285  ..I.IM.A.IFE.I..RMAPSIM.S....T..EV  318
```

```
AAC27646      265  ..I.IM.T.IFE.I..RMAPSIM.S....T..EV  298
BAE31548      266  ..I.IM.T.IFE.I..RMAPSIM.S..G.T..EV  299
XP_001496903  266  ..I.IM.A.IFE.I..RMAPSIM.S....T..EV  299
BAE35165      265  ..I.IM.T.IFE.I..RMAPSIM.S....T..EV  298
XP_540090     264  ..I.IM.A.IFE.I..RMAPSIM.S....T..EV  297
CAH92473      265  ..I.IM.A.IFE.I..RMAPSIM.S....T..EV  298
CAH91518      264  ..I.IM.A.IFE.I..RMAPSIM.S....T..EV  297
NP_001026195  261  ..I.IM..SI.EYI..RMATSIM.S.....V.EV  294
EDM11636      209  ..I.IM.A.IFE.I..RMAPSIM.S....T..EV  242
XP_001379494  264  ..I.IM.A..FE.IM.RMASNTV.N....AV.EV  297
EAT43140      298  I......TII..V.M.KL.TSFIRGK....V..   330
ABF18402      300  I......TII..V.M.KL.TSFIRGK....V..   332
XP_001157154  209  ..I.IM.A.IFE.I..RMAPSIM.S....T..EV  242
XP_875149     259  I.L.I..TVI.E..V.KL.PTL.HHT.......V  292
NP_001079704  257  .......NKI.E..V.RL.S.L..NS....V.EV  290
NP_999963     273  ..I..M.VVIFE..M.RM.SSIV.S.......EV  306
XP_001167866  336  ..L.I...VI.E..V.KL.PVL.HRT.......   368
XP_001498897  259  ..L.I..TVI.E..V.RL.LTL.HHT........  292
XP_001112885  259  ..L.I...VI.E..V.KL.PVL.HHT.......   291
EDL05918      281  I.L.I..TVI.E....RL.PLL.HHT.......   313
NP_997856     266  M.I.IM.K.IFE....KM...L..SS....V.EV  299
XP_318581     299  M.L.IV.KMI..V...KL.TSW.RGK....V..   331
CAC21716      259  ..L.I...VI.E..V.KLPPVL.HHT.......   291
XP_001367355  259  ..L.I..TVI.E....KL.ATL.HTS.......   291
NP_663510     259  I.L.I..TVI.E....RL.PLL.HHT.......   291
EAX10639      249  ..L.I...VI.E..V.KLPPVL.HHT.......   281
NP_536737     259  ..L.I...VI.E..V.KLPPVL.HHT.......   291
XP_542935     254  ..L.I..TVI.E..V.KL.PTL.HHA.......V  287
XP_001112910  174  ..L.I...VI.E..V.KL.PVL.HHT.......   206
AAD20049      174  ..L.I...VI.E..V.KLPPVL.HHT.......   206
NP_056500     174  ..L.I...VI.E..V.KLPPVL.HHT.......   206
NP_001020863  263  I.L.I..TVI.E....KL.PLL.HHT.......   295
NP_001040345  255  .NI.I..YGI.ER..NKM.SSLF.E.          280
XP_857475     186  I.M..                               190
XP_001514333  262  I.I.IM.A.IFE.I..RMASSIM.T....T..EV  295
XP_001514311  268  I.I.IM.A.IFE.I..RMASSIM.T....T..EV  301
1OBZ-A        158  ..I.IM.A.                           166
XP_001087599  211  ..I.IM.A.IFE.I..RMAPSIM.S....T..EV  244
XP_001157051  211  ..I.IM.A.IFE.I..RMAPSIM.S....T..EV  244
CAG14594      131  M....M.K.I.E....RM.T.LMRSV......EV  164
1N99-A        158  ..I.IX.A.                           166
EAW86816      185  I.M.I                               189
CAM27238      186  I.M.I                               190
EAW86813      185  I.M.I                               189
CAG14595      160  M....M.K.I.E....RM.T.LMRSV......EV  193
EDM11637      78   ..I.IM.A.IFE.I..RMAPSIM.S....T..EV  111
XP_001492473  175  ..I.IM.A.IFE.I..QMAPSIM.S....T..EV  208
```

```
XP_001477912  88   A.I.IV.A.IFKYI..WMAPSIM.S....T..EV  121
CAM27241      187  I.M.I                               191
```
