# Supplementary material for: Global comparative analysis of ESTs from the southern cattle tick, Rhipicephalus (Boophilus) microplus
Source: BMC Genomics. 2007 Oct 12;8:368. doi: 10.1186/1471-2164-8-368 (PMC2100071; doi:10.1186/1471-2164-8-368)
Supplement: Additional file 6 — Alignment from Clustal W for TC13445. This file shows the alignment which was used to generate the phylogenetic tree for TC13445 [file 1471-2164-8-368-S6.pdf]

Alignment view for rid: 8JRAV13X012, query ID: lcl|1_3823, database: nr

Mouse over the sequence identifer for sequence title

```
1_3823          1     KADTKIIASGSDDAK-VKLW--------S-------L-----------------------  21
XP_697907       488     .P.LL........-....--------.-------T-----------------------  506
NP_001016199    478     .P.LL........-....--------.-------T-----------------------  496
XP_001515470    410     .P.LL........-....--------.-------T-----------------------  428
XP_426628       471     .P.LL........-....--------.-------T-----------------------  489
NP_001083011    488     .P.LL........-....--------.-------T-----------------------  506
EAW90999        564     .P.LL........-....--------.-------T-----------------------  582
XP_001104796    525     .P.LL........-....--------.-------T-----------------------  543
XP_001104719    505     .P.LL........-....--------.-------T-----------------------  523
NP_071902       525     .P.LL........-....--------.-------T-----------------------  543
EAW91002        324     .P.LL........-....--------.-------T-----------------------  342
AAI33613        529     .P.LL........-....--------.-------T-----------------------  547
BAB15239        300     .P.LL........-....--------.-------T-----------------------  318
NP_001001740    501     .P.LL........-....--------.-------T-----------------------  519
XP_001153038    505     .P.LL........-....--------.-------T-----------------------  523
XP_537181       527     .P.LL........-....--------.-------T-----------------------  545
XP_001493699    406     .P.LL........-....--------.-------T-----------------------  424
EDM09451        527     .P.LL........-....--------.-------T-----------------------  545
NP_036061       527     .P.LL........-....--------.-------T-----------------------  545
XP_993328       467     .P.LL........-....--------.-------T-----------------------  485
XP_001479473    507     .P.LL........-....--------.-------T-----------------------  525
CAH72422        285     .P.LL........-....--------.-------T-----------------------  303
CAH72423        358     .P.LL........-....--------.-------T-----------------------  376
NP_001020297    227     .P.LL........-....--------.-------T-----------------------  245
XP_974586       458   EV..RL........R-...Y--------.-------.-----------------------  478
XP_001373596    534     .P.LL........-....--------.-------T-----------------------  552
CAG10910        547     .P.LL........-....--------.-------T-----------------------  565
EAW91001        265     .P.LL........-....--------.-------T-----------------------  283
NP_001071934    443     .PRLL.......R-....--------.-------T-----------------------  461
XP_001183774    102   RM.P.LL........-....--------.-------T-----------------------  122
XP_001192151    13    RM.P.LL........-....--------.-------T-----------------------  33
XP_001105376    248                SR-....--------Y-------T-----------------------  255
XP_636746       768   RT.PTQL......TR-....--------.-------T-----------------------  788
AAA32772        473   RTEPSMLV.....C.-..V.--------C-------T-----------------------  493
NP_180854       473   RTEPSMLV.....C.-..V.--------C-------T-----------------------  493
CAB89693        473   RT.PSMLV.....C.-..V.--------C-------T-----------------------  493
CAB94800        768   RT.PSMLV.....C.-..V.--------C-------T-----------------------  788
P93471          470   RT.PSMLV.....C.-..V.--------C-------T-----------------------  490
AAG31173        475   RT.PSMLV.....C.-..V.--------C-------T-----------------------  495
AAK81856        462     EPSMLV.....C.-..V.--------C-------T-----------------------  480
AAC98912        475   RTEPSMLV.....C.-..V.--------C-------T-----------------------  495
EAY87686        434   RTEPSMLV.....C.-..V.--------C-------T-----------------------  454
CAN71084        480         LV.....C.-..V.--------C-------T-----------------------  494
EAZ24764        483   RTEPSMLV.....C.-..V.--------C-------T-----------------------  503
NP_001048254    483   RTEPSMLV.....C.-..V.--------C-------T-----------------------  503
```

```
BAA94422       483   RTEPSMLV.....C.-..V.--------C-------T-----------------------  503
BAD16847       402   RTEPSMLV.....C.-..V.--------C-------T-----------------------  422
AAK49415       317   RTEPSMLV.....C.-..V.--------C-------T-----------------------  337
CAB89694       768   RT.PSMLV.....C.Q..V.--------C-------T-----------------------  789
BAB02165       637    ..PTLL......GT-....--------.-------I-----------------------  656
NP_175717      587    ..PTLL......GS-....--------.-------I-----------------------  606
BAF01052       895         L......CS-....--------N-------I-----------------------  909
BAF01052       798     .EDYF.TAGVSK.-I.IYEFNSLFNE.-------V-----------------------  824
NP_192849      831         L......CS-....--------N-------I-----------------------  845
NP_192849      734     .EDYF.TAGVSK.-I.IYEFNSLFNE.-------V-----------------------  760
NP_182157      819    S.PTKFV.....CS-....--------.-------I-----------------------  838
BAE99225       819    S.PTKFV.....CS-....--------.-------I-----------------------  838
AAD23037       322    S.PTKFV.....CS-....--------.-------I-----------------------  341
BAD94577       31     S.PTKFV.....CS-....--------.-------I-----------------------  50
NP_001044115   595    ..PTKLV.....GS-....--------NMNQAILF.-----------------------  621
NP_001056370   933   EV.PTKL......CC-..V.--------.-------I-----------------------  953
CAB43046       799         L......CS-....--------N-------INEACSSILDFVVLIATPTEISGI  836
NP_683567      637    ..PTLL......GT---------------------------------------------  650
AAC35546       238         L......CS-....--------N-------INEACSSILDFVVLIATPTEISGI  275
EAZ13397       604    ..PTKLV.....GS-....--------NMNQAILF.-----------------------  630
EAY75677       600    ..PTKLV.....GS-....--------NMNQAILF.-----------------------  626
XP_001420221   105     .PTKL..A...GT-.RIF--------.-------T-----------------------  123
CAN60249       30      .PTKF......CS---------------------------------------------  42
CAL56309       671     .PTKL......GT-.RVF--------.-------T-----------------------  689
CAL53135       439   RSNPSLLV.....YL-..I.--------N-------T-----------------------  459
CAN64346       360    ..PTKL...G..GA-....--------N-------I-----------------------  379
EAY99095       862         L..TDY.GT-.Q..--------D-------A-----------------------  876
EAZ35341       862         L..TDY.GT-.Q..--------D-------A-----------------------  876
NP_568435      163          ......GT-MQV.--------D-------P-----------------------  176
AAK64045       38           ......GT-MQV.--------D-------P-----------------------  51
XP_991149      60      .P.LL........-....--------.-------T-----------------------  78
AAM64761       184          ......GT-.QM.--------D-------------------------------  196
NP_200038      182        .G......GT-.QM.--------D-------------------------------  196
NP_487506      1169    .GATL....G.QT-.R..--------D-------I-----------------------
1187
NP_487506      1211    .GSTL....S.QT-.R..--------E-------I-----------------------
1229
NP_487506      917     .G.ML......QT-.R..--------D-------I-----------------------  935
NP_487506      1253    .GSML....S.KT-.R..--------D-------I-----------------------
1271
NP_487506      1337    .GTML......QT-.R..--------.-------I-----------------------
1355
NP_487506      1042   S.GAML......QT-.R..--------D-------I-----------------------
1061
NP_487506      875     .G.LF.T.DSGGI-.RF.--------E-------A-----------------------  893
NP_487506      1379    .GA.L....G.QT-.R..--------.-------I-----------------------
1397
BAD07933       178        MV......RT-AHV.--------DPRAP---A-----------------------  197
```

```
EAY84193      178        MV......RT-AHV.--------DPRAP---A-----------------------  197

1_3823        22    --------SC---------DHSV-----ASLE------A-KAN-VCCVKFNPDSRYHLAL  51
XP_697907     507   --------NL---------.N..-----..I.------.-...-......S.T......F  536
NP_001016199  497   --------NL---------.N..-----..I.------.-...-......S.S......F  526
XP_001515470  429   --------NL---------.N..-----..I.------.-...-......S.S......F  458
XP_426628     490   --------NL---------.N..-----..I.------.-...-......S.S......F  519
NP_001083011  507   --------NL---------.N..-----..I.------.-...-......S.T......F  536
EAW90999      583   --------NL---------.N..-----..I.------.-...-......S.S......F  612
XP_001104796  544   --------NL---------.N..-----..I.------.-...-......S.S......F  573
XP_001104719  524   --------NL---------.N..-----..I.------.-...-......S.S......F  553
NP_071902     544   --------NL---------.N..-----..I.------.-...-......S.S......F  573
EAW91002      343   --------NL---------.N..-----..I.------.-...-......S.S......F  372
AAI33613      548   --------NL---------.N..-----..I.------.-...-......S.S......F  577
BAB15239      319   --------NL---------.N..-----..I.------.-...-......S.S......F  348
NP_001001740  520   --------NL---------.N..-----..I.------.-...-......S.S......F  549
XP_001153038  524   --------NL---------.N..-----..I.------.-...-......S.S......F  553
XP_537181     546   --------NL---------.N..-----..I.------.-...-......S.S......F  575
XP_001493699  425   --------NL---------.N..-----..I.------.-...-......S.S......F  454
EDM09451      546   --------NL---------.N..-----..I.------.-...-......S.S......F  575
NP_036061     546   --------NL---------.N..-----..I.------.-...-......S.S......F  575
XP_993328     486   --------NL---------.N..-----..I.------.-...-......S.S......F  515
XP_001479473  526   --------NL---------.N..-----..I.------.-...-......S.S......F  555
CAH72422      304   --------NL---------.N..-----..I.------.-...-......S.S......F  333
CAH72423      377   --------NL---------.N..-----..I.------.-...-......S.S......F  406
NP_001020297  246   --------NL---------.N..-----..I.------.-...-......S.S......F  275
XP_974586     479   --------NG---------E..I-----.T..------.-...-........R.SC...F  508
XP_001373596  553   --------NL---------.N..-----..I.------.-...-......S.S......F  582
CAG10910      566   --------NL---------.N..-----..I.------.-...-......S.T......F  595
EAW91001      284   --------NL---------.N..-----..I.------.-...-......S.S......F  313
NP_001071934  462   --------GV---------QR..-----.CI.------.-...-....Q...H.AF...F  491
XP_001183774  123   --------NQ---------EQ.I-----T...------.-...-........TKM.G..F  152
XP_001192151  34    --------NQ---------EQ.I-----T...------.-...-........TKM.G..F  63
XP_001105376  256   --------NL---------.S..-----..T.------.-...-......S.SF.....F  285
XP_636746     789   --------TT---------ERAI-----TTI.------S-...-I.......C.SNLI.F  818
EAX01619      67                       NN..-----..I.------.-..D-......SHS..C...F  94
AAA32772      494   --------RQ---------EA..-----INID------M-...-I....Y..G.SNYI.V  523
NP_180854     494   --------RQ---------EA..-----INID------M-...-I....Y..G.SNYI.V  523
CAB89693      494   --------NQ---------EA..-----LNID------M-...-I....Y..G.GNYI.V  523
CAB94800      789   --------NQ---------EA..-----LNID------M-...-I....Y..G.GNYI.V  818
P93471        491   --------NQ---------EA..-----LNID------M-...-I....Y..G.GNYI.V  520
AAG31173      496   --------KQ---------EA.A-----LNID------M-...-I....Y..G.SF.V.V  525
AAG31173      416                         .-----VEMS------T-RSK-LS.LSW.KYTKN.I.S  440
AAK81856      481   --------RQ---------EA..-----LNID------M-...-I....Y..G.SNCI.V  510
AAC98912      496   --------KQ---------EA..-----LNID------M-...-I....Y..G.SV.I.V  525
AAC98912      416                         .-----VEMS------T-RSK-LS.LSW.KYTKN.I.S  440
EAY87686      455   --------KQ---------EA.A-----INID------M-...-I.S..Y..G.SHYV.V  484
CAN71084      495   --------NQ---------EA..-----LNID------M-...-I....Y..G.SIYI.V  524
```

```
CAN71084       415                        .-----VEMS------T-RSK-LS.LSW.KCTKN.I.S  439
EAZ24764       504    --------KQ---------EA.A-----INID------M-...-I.S..Y..G.SHYV.V  533
NP_001048254   504    --------KQ---------EA.A-----INID------M-...-I.S..Y..G.SHYV.V  533
BAA94422       504    --------KQ---------EA.A-----INID------M-...-I.S..Y..G.SHYV.V  533
BAD16847       423    --------KQ---------EA.A-----INID------M-...-I.S..Y..G.SHYV.V  452
AAK49415       338    --------KQ---------EA.A-----INID------M-...-I.S..Y..G.SHYV.V  367
CAB89694       790    --------NQ---------EA..-----LNID------M-...-I....Y..G.GNYI.V  819
BAB02165       657    --------NQ---------GV.I-----GTIK------T-...-....Q.PS..GRS..F  686
NP_175717      607    --------NQ---------GV.I-----GTIK------T-...-I...Q.PSETGRS..F  636
BAF01052       910    --------NE---------RNCL-----GTIR------N-I..-....Q.S.Q.SHL..F  939
BAF01052       825    --------DI---------HYPA-----IEMP------N-RSK-LSG.CW.NYI.NY..S  854
NP_192849      846    --------NE---------RNCL-----GTIR------N-I..-....Q.S.Q.SHL..F  875
NP_192849      761    --------DI---------HYPA-----IEMP------N-RSK-LSG.CW.NYI.NY..S  790
NP_182157      839    --------NE---------KR.L-----GTIW------S-P..-....Q.SSY.NHL..F  868
BAE99225       839    --------NE---------KR.L-----GTIW------S-P..-....Q.SSY.NHL..F  868
AAD23037       342    --------NE---------KR.L-----GTIW------S-P..-....Q.SSY.NHL..F  371
BAD94577       51     --------NE---------KR.L-----GTIW------S-P..-....Q.SSY.NHL..F  80
NP_001044115   622    --------HL---------AG..-----GTIR------T-R..-..S.Q.Q...ARSI.I  651
NP_001056370   954    --------NQ---------KNCT-----DTIR------N-V..-....Q.S.Y.SRM..F  983
CAB43046       837    YTYEYINSTL---------.Y..NRNCLGTIR------N-I..-....Q.S.Q.SHL..F  879
NP_683567      651    -------------------GV.I-----GTIK------T-...-....Q.PS..GRS..F  678
AAC35546       276    YTYEYINSTL---------.Y..NRNCLGTIR------N-I..-....Q.S.Q.SHL..F  318
EAZ13397       631    --------HL---------AG..-----GTIR------T-R..-..S.Q.Q...ARSI.I  660
EAY75677       627    --------HL---------AG..-----GTIR------T-R..-..S.Q.Q...ARSI.I  656
XP_001420221   124    --------TT---------KEG.-----CT.Q------N-R..-..S...H.T.AHM..I  153
CAN60249       43     -------------------RN.T-----STIW------N-P..-....Q.SAY.THL.VF  70
CAL56309       690    --------TT---------KEAT-----CTIQ------N-H..-..S.R.H.TAPHL..I  719
CAL53135       460    --------DQ---------RN..-----HEID------M-E..-....Q.S.NDDHGI.I  489
CAN64346       380    --------NQAILFLHLVDGG.I-----GTIK------T-...-....Q.P...ARS..I  418
EAY99095       877    --------.S---------GQGF-----TQFT------E-HRK-RAW-S.S.Y.SRM..F  905
EAZ35341       877    --------.S---------GQGF-----TQFT------E-HRK-RAW-S.S.Y.SRM..F  905
NP_568435      177    --------R.PP-------EE..-----GVVRPAGI--C-RSA-....E.D.SGGPAV.V  212
AAK64045       52     --------R.PP-------EE..-----GVVRPAGI--C-RSA-....E.D.SGGPAV.V  87
XP_991149      79     --------NL---------.N..------..I.------.-N..-......S.S.....TF  108
CAA04169       1                                         ..-I....Y.RG.SNYI.V  18
AAM64761       197    -------------------PRNG-----GT..ETVRPGG-G.A-I.S.E.D.FGGSSI.V  230
NP_200038      197    -------------------PRNG-----GT..ETVRPGG-G.A-I.S.E.D.FGGSSI.V  230
NP_487506      1188   --------.S---------SKCL-----YI.Q------G-HTSW.NS.V....GST-..S
1217
NP_487506      1230   --------NS---------SKCL-----CTFQ------G-HTSW.NS.V....GSM-..S
1259
NP_487506      936    --------.S---------GQCL-----KTFK------GHTSR-.RS.V.S.N.-LM..S  965
NP_487506      1272   --------.S---------SKCL-----HTFQ------G-HT.W.NS.A....GSM-..S
1301
NP_487506      1356   --------.S---------GECL-----YTFL------G-HT.W.GS.I.S..GAI-..S
1385
NP_487506      1062   --------.S---------GNCL-----YT.Q------G-HTSC.RS.V.S..GAM-..S
1091
```

```
NP_487506       894    --------AT---------GKEL-----LTCK------G-HNSW.NS.G.SQ.GKM-..S  923
NP_487506       1398   --------.S---------GKCL-----YT.Q------G-HN.W.GSIV.S..GTL-..S
1427
BAD07933        198    --------GA---------AG.W-----.TAR------.-GGA-.L..E.D.AGGPQ..V  227
EAY84193        198    --------GA---------AG.W-----.TAR------.-GGA-.L..E.D.AGGPQ..V  227

1_3823          52     GSADHCVHYYDLRSL-KQPLGVFKGHKKAVSYVKF-L----NT--T-ELVSA--------  94
XP_697907       537    .C...........NT-...IM.....R.....A..-V----.G--E-.I...--------  579
NP_001016199    527    .C...........NT-...IM.....R.....A..-V----.G--D-.I...--------  569
XP_001515470    459    .C...........NT-...IM.....R.....A..-V----SG--E-.I...--------  501
XP_426628       520    .C...........NT-...IM.....R.....A..-V----SG--E-.I...--------  562
NP_001083011    537    .C...........NA-...IM.....R.....A..-V----.G--G-.I...--------  579
EAW90999        613    .C...........NT-...IM.....R.....A..-V----SG--E-.I...--------  655
XP_001104796    574    .C...........NT-...IM.....R.....A..-V----SG--E-.I...--------  616
XP_001104719    554    .C...........NT-...IM.....R.....A..-V----SG--E-.I...--------  596
NP_071902       574    .C...........NT-...IM.....R.....A..-V----SG--E-.I...--------  616
EAW91002        373    .C...........NT-...IM.....R.....A..-V----SG--E-.I...--------  415
AAI33613        578    .C...........NT-...IM.....R.....A..-V----SG--E-.I...--------  620
BAB15239        349    .C...........NT-...IM.....R.....A..-V----SG--E-.I...--------  391
NP_001001740    550    .C...........NT-...IM.....R.....A..-V----SG--E-.I...--------  592
XP_001153038    554    .C...........NT-...IM.....R.....A..-V----SG--E-.I...--------  596
XP_537181       576    .C...........NT-...IM.....R.....A..-V----SG--E-.I...--------  618
XP_001493699    455    .C...........NT-...IM.....R.....A..-V----SG--E-.I...--------  497
EDM09451        576    .C...........NT-...IM.....R.....A..-V----SG--E-.I...--------  618
NP_036061       576    .C...........NT-...IM.....R.....A..-V----SG--E-.I...--------  618
XP_993328       516    .C...........NT-...IM.....R.....A..-V----SG--E-.I...--------  558
XP_001479473    556    .C...........NT-...IM.....R.....A..-V----SG--E-.I...--------  598
CAH72422        334    .C...........NT-...IM.....R.....A..-V----SG--E-.I...--------  376
CAH72423        407    .C...........NT-...IM.....R.....A..-V----SG--E-.I...--------  449
NP_001020297    276    .C...........NT-...IM.....R.....A..-V----SG--E-.I...--------  318
XP_974586       509    .............NM-.EAVA..............-.----.S--E-DI...--------  551
XP_001373596    583    .C...........NT-...IM.....R.....A..-V----SG--E-.I...--------  625
CAG10910        596    .C...........NT-...IM.....R.....A..-V----SG--E-.I...QSLSEHNW  646
EAW91001        314    .C...........NT-...IM.....R.....A..-V----SG--E-.I...--------  356
NP_001071934    492    .C...F.....I.NT-..SVS..R........A..-V----DK--D-.I...--------  534
XP_001183774    153    .............HP-....N.....R.....T..-V----.S--E-.I...--------  195
XP_001192151    64     .............HP-....N.....R.....T..-V----.S--E-.I...--------  106
XP_001105376    286    .C..........HNT-E..IM.....Q.....A..-V----SG--E-.M.F.--------  328
XP_636746       819    .....HI......QY-.D..LI....R........-M----.K--D-.II..--------  861
EAX01619        95     .C............T-...IM....RQ.....A..-A----SG--E-.I.F.--------  137
AAA32772        524    .....HI......NI-S...H..S...........-.----SN--N-..A..--------  566
NP_180854       524    .....HI......NI-S...H..S...........-.----SN--N-..A..--------  566
CAB89693        524    .....HI......NI-SR.VH..T...........-.----SN--D-..A..--------  566
CAB94800        819    .....HI......NI-SR.VH..T...........-.----SN--D-..A..--------  861
P93471          521    .....HI......NI-SR.VH..T...........-.----SN--D-..A..--------  563
AAG31173        526    .....HI......NT-SA..HI.S...........-.----SS--H-..A..--------  568
AAG31173        441    SDY.GI.TVW.VTT--R.SVMEYEE.E.RAWS.D.-S----R.DPS-M...G--------  484
AAK81856        511    .....HI......NV-S...H..T..R........-.----SN--Y-..A..--------  553
```

```
AAC98912      526   .....HI.....NT-S..VHI.S..R.........-.----SN--N-..A.--------  568
AAC98912      441   SDY.GI.TVW.VTT--R.SVMEYEE.E.RAWS.D.-S----R.EPS-M...G--------  484
EAY87686      485   .....HI..F...NP-SA.VH..G...........-.----S.--N-..A.--------  527
CAN71084      525   .....HI.....NT-SH..HI.S............-.----SN--N-..A.--------  567
CAN71084      440   SDYEGI.TVW.VNT--R.SVMEYEE.E.RAWS.D.-S----R.EPS-R...G--------  483
EAZ24764      534   .....HI..F...NP-SA.VH..G...........-.----S.--N-..A.--------  576
NP_001048254  534   .....HI..F...NP-SA.VH..G...........-.----S.--N-..A.--------  576
BAA94422      534   .....HI..F...NP-SA.VH..G...........-.----S.--N-..A.--------  576
BAD16847      453   .....HI..F...NP-SA.VH..G...........-.----S.--N-..A.--------  495
AAK49415      368   .....HI..F...NP-SA.VH..G...........-.----S.--N-..A.--------  410
CAB89694      820   .....HI.....NI-SR.VH..T............-.----SN--D-..A.--------  862
BAB02165      687   .....K.Y.....NP-.I..CTMI..S.T......-V----DS--S-T...S--------  729
NP_175717     637   .....K.Y.....NP-.L..CTMI..H.T....R.-V----DS--S-T...S--------  679
BAF01052      940   ..S.FRTYC....N.-RT.WCILS..N.....A..-.----DN--E-T..T.--------  982
BAF01052      855   SDY.GI.KLW.VTT--G.AISH.IE.E.RAWS.D.-S----EACP.-K.A.G--------  898
NP_192849     876   ..S.FRTYC....N.-RT.WCILS..N.....A..-.----DN--E-T..T.--------  918
NP_192849     791   SDY.GI.KLW.VTT--G.AISH.IE.E.RAWS.D.-S----EACP.-K.A.G--------  834
NP_182157     869   ....YK.YC....YV-.T.WCTLA..E........-M----DS--E-TI..---------  911
BAE99225      869   ....YK.YC....YV-.T.WCTLA..E........-M----DS--E-TI..---------  911
AAD23037      372   ....YK.YC....YV-.T.WCTLA..E........-M----DS--E-TI..---------  414
BAD94577      81    ....YK.YC....YV-.T.WCTLA..E........-M----DS--E-TI..---------  123
NP_001044115  652   .....KIYC....NI-RA.YCTLV..T.T.....Y-V----DA--S-TI..---------  694
NP_001056370  984   ....YKIYC....NT-RI.WCTIS..G......R.-.----DP--E-T.I..--------
1026
XP_001140892  2                   .....Q.V...A..-V----GG--E-.I..---------  24
CAB43046      880   ..S.FRTYC....N.-RT.WCILS..N.....A..-.----DN--E-T..T.--------  922
NP_683567     679   .....K.Y.....NP-.I..CTMI..S.T......-V----DS--S-T...S--------  721
AAC35546      319   ..S.FRTYC....N.-RT.WCILS..N.....A..-.----DN--E-T..T.--------  361
XP_001130134  2                   ....RQ.....A..-A----SG--E-.I.F.--------  24
EAZ13397      661   .....KIYC....NI-RA.YCTLV..T.T.....Y-V----DA--S-TI..---------  703
EAY75677      657   .....KIYC....NI-RA.YCTLV..T.T.....Y-V----DA--S-TI..---------  699
XP_001420221  154   .....RI.V....QP-ST..MTLQ..R......HW-V----G---D-..............  195
CAN60249      71    ....YKIYG....HT-RI.WC.LA..Q........-.----DS--E-T...---------  113
CAL56309      720   ...N.KI.C....Q.-NN..LTLQ..R......YW-V----G---D-..L.--------  761
CAL53135      490   SCVNQKAYIF...R.-DE..H.LEA.R.....I.Y-.----.A--K-.V.T.--------  532
CAN64346      419   .....K.YC....NT-RI..ATLN..S.T......-I----.S--.-T...---------  461
EAY99095      906   ....YKIYC....NT-RI.WCTIS..G......R.-.----DP--E-T.I..--------  948
EAZ35341      906   ....YKIYC....NT-RI.WCTIS..G......R.-.----DP--E-T.I..--------  948
NP_568435     213   .C..RKGYV..I.K.-VD.ALTLQ..T.T....R.-.----DG--G-TV.T.--------  255
AAK64045      88    .C..RKGYV..I.K.-VD.ALTLQ..T.T....R.-.----DG--G-TV.T.--------  130
XP_991149     109   .C........N.HNT-.K.IM.....Q.....A..-V----S                   144
CAA04169      19    .....HI.....NI-S...H..S............-.----SN--N-..A.--------  61
AAM64761      231   .C..QNAYV..I.R.-VD..I.LD..T.T.T.AR.-M----DS--H-TI.TG--------  273
NP_200038     231   .C..RNAYV..I.R.-VD..I.LD..T.T.T.AR.-M----DS--H-TI.TG--------  273
NP_487506     1218  ..S.QT.RLWEIN.S-.-C.CT.Q..TSW.NS.V.NP----DG--S-M.A.G--------
1260
NP_487506     1260  ..S.KT.RLW.IS.S-.-C.HT.Q..TNW.NS.A.NP----DG--S-M.A.G--------
1302
```

```
NP_487506      966   ..S.QT.RLW.IS.--GEC.YI.Q..TGW.YS.A.N.----DG--S-M.ATG--------
1008
NP_487506      1302  ..G.QT.RLWEIS.S-.-C.HT.Q..TSW..S.T.-SP---DG--.-M.A.G--------
1344
NP_487506      1386  ..G.QT.RLWSIS.G-.-C.YTLQ..NNW.GSIV.-SP---DG--.-L.A.G--------
1428
NP_487506      1092  .GD.QI.RLW.IS.--GNC.YTLQ.Y---T.W.R.-.VFSP.G--V-T.ANG--------
1134
NP_487506      924   ..D.QT.RLW.IS.--G.C.KT....TSR.RS.V.-SP---.S--L-M.A.G--------  966
NP_487506      1428  ..D.QT.RLWNIS.--GEC.YTLH..INS.RS.A.-SS---DG--L-I.A.G--------
1470
BAD07933       228   ....RRAAVH.V.A.GRGAVASMD..GR..T..RW-A----A.--ARRV.TS--------  272
EAY84193       228   ....RRAAVH.V.A.GRGAVASMD..GR..T..RW-A----AA--ARRV.TS--------  272

1_3823         95    ----------------------------------------STDSQLKLWNIS------K-  107
XP_697907      580   ----------------------------------------..........VN------.-  592
NP_001016199   570   ----------------------------------------..........V.------R-  582
XP_001515470   502   ----------------------------------------..........VG------.-  514
XP_426628      563   ----------------------------------------..........VG------.-  575
NP_001083011   580   ----------------------------------------..........VN------.-  592
EAW90999       656   ----------------------------------------..........VG------.-  668
XP_001104796   617   ----------------------------------------..........VG------.-  629
XP_001104719   597   ----------------------------------------..........VG------.-  609
NP_071902      617   ----------------------------------------..........VG------.-  629
EAW91002       416   ----------------------------------------..........VG------.-  428
AAI33613       621   ----------------------------------------..........VG------.-  633
BAB15239       392   ----------------------------------------..........VG------.-  404
NP_001001740   593   ----------------------------------------..........VG------.-  605
XP_001153038   597   ----------------------------------------..........VG------.-  609
XP_537181      619   ----------------------------------------..........VG------.-  631
XP_001493699   498   ----------------------------------------..........VG------.-  510
EDM09451       619   ----------------------------------------..........VG------.-  631
NP_036061      619   ----------------------------------------..........VG------.-  631
XP_993328      559   ----------------------------------------..........VG------.-  571
XP_001479473   599   ----------------------------------------..........VG------.-  611
CAH72422       377   ----------------------------------------..........VG------.-  389
CAH72423       450   ----------------------------------------..........VG------.-  462
NP_001020297   319   ----------------------------------------..........VG------.-  331
XP_974586      552   ----------------------------------------..........VN------T-  564
XP_001373596   626   ----------------------------------------..........VG------.-  638
CAG10910       647   PLLGPSRVPAHRGKTRGHFYFCLDQTEMLAWLSSEDDVYR..........V.------.-  699
EAW91001       357   ----------------------------------------..........VG------.-  369
NP_001071934   535   ----------------------------------------....E.R..KT.------T-  547
XP_001183774   196   ----------------------------------------..........VD------.-  208
XP_001192151   107   ----------------------------------------..........VD------.-  119
XP_001105376   329   ----------------------------------------..........VG------.-  341
XP_636746      862   ----------------------------------------....T.....VN------Q-  874
EAX01619       138   ----------------------------------------......T...VG------.-  150
```

```
AAA32772      567   ----------------------------------------....T.R..DVK------D-  579
NP_180854     567   ----------------------------------------....T.R..DVK------D-  579
CAB89693      567   ----------------------------------------....T.R..DVK------Q-  579
CAB94800      862   ----------------------------------------....T.R..DVK------Q-  874
P93471        564   ----------------------------------------....T.R..DVK------Q-  576
AAG31173      569   ----------------------------------------....T.R..DVK------D-  581
AAG31173      485   ----------------------------------------.D.CKV.V.             493
AAK81856      554   ----------------------------------------....T.R...VK------D-  566
AAC98912      569   ----------------------------------------....T.R..DVK------D-  581
AAC98912      485   ----------------------------------------.D.CKV.V.             493
EAY87686      528   ----------------------------------------....T.R..DVK------E-  540
CAN71084      568   ----------------------------------------....T.R..DVK------E-  580
CAN71084      484   ----------------------------------------.D.CKV.V.             492
EAZ24764      577   ----------------------------------------....T.R..DVK------E-  589
NP_001048254  577   ----------------------------------------....T.R..DVK------E-  589
BAA94422      577   ----------------------------------------....T.R..DVK------E-  589
BAD16847      496   ----------------------------------------....T.R..DVK------E-  508
AAK49415      411   ----------------------------------------....T.R..DVK------E-  423
CAB89694      863   ----------------------------------------....T.R..DVK------Q-  875
BAB02165      730   ----------------------------------------...NT....DL.------M-  742
NP_175717     680   ----------------------------------------...NT....DL.MSISGIN-  698
BAF01052      983   ----------------------------------------...NT....DLK------.-  995
BAF01052      899   ----------------------------------------.D.CSV.....N------E-  911
NP_192849     919   ----------------------------------------...NT....DLK------.-  931
NP_192849     835   ----------------------------------------.D.CSV.....N------E-  847
NP_182157     912   ----------------------------------------...NS.....LN------.-  924
BAE99225      912   ----------------------------------------...NS.....LN------.-  924
AAD23037      415   ----------------------------------------...NS.....LN------.-  427
BAD94577      124   ----------------------------------------...NS.....LN------.-  136
NP_001044115  695   ----------------------------------------...NS....DL.------M-  707
NP_001056370  1027  ----------------------------------------...NT..I.DLN------Q-
1039
XP_001140892  25    ----------------------------------------......T...VG------.-  37
CAB43046      923   ----------------------------------------...NT....DLK------.-  935
NP_683567     722   ----------------------------------------...NT....DL.------M-  734
AAC35546      362   ----------------------------------------...NT....DLK------.-  374
XP_001130134  25    ----------------------------------------......T...VG------.-  37
EAZ13397      704   ----------------------------------------...NS....DL.------M-  716
EAY75677      700   ----------------------------------------...NS....DL.------M-  712
XP_001420221  196   ----------------------------------------...NT....D.KRNDP--R-  212
CAN60249      114   ----------------------------------------...NT....DLN------.-  126
CAL56309      762   ----------------------------------------...NT....DVK------R-  774
CAL53135      533   ----------------------------------------...NTINV..TN------S-  545
CAN64346      462   ----------------------------------------....S....DL.------TC  475
EAY99095      949   ----------------------------------------...NT..I.DLN------R-  961
EAZ35341      949   ----------------------------------------...NT..I.DLN------Q-  961
NP_568435     256   ----------------------------------------G..GC....SVE------D-  268
AAK64045      131   ----------------------------------------G..GC....SVE------D-  143
```

```
CAA04169       62    ----------------------------------------....T.R..DVK------D-  74
AAM64761       274   ----------------------------------------...GS..Q.D.D------NG  287
NP_200038      274   ----------------------------------------...GS..Q.D.D------NG  287
NP_487506      1261  ----------------------------------------.S.KTVR..D..------S-
1273
NP_487506      1303  ----------------------------------------.G.QTVR..E..------S-
1315
NP_487506      1009  ----------------------------------------.G.QTVR..D..------S-
1021
NP_487506      1345  ----------------------------------------.D.QTVR..S..------S-
1357
NP_487506      1429  ----------------------------------------.D.QTVR.....------S-
1441
NP_487506      1135  ----------------------------------------.S.QIVR..D..------S-
1147
NP_487506      967   ----------------------------------------.S.QTVR..D..------S-  979
NP_487506      1471  ----------------------------------------.D.ETI...DVK------T-
1483
BAD07933       273   ----------------------------------------AA.GTHR..ALP------A-  285
EAY84193       273   ----------------------------------------AA.GTHR..ALP------A-  285

1_3823         108   -----P--------H--------C-------LRSFKGHLNEKNFVGLAT--DGD-YVACG  136
XP_697907      593   -----.--------.--------.-------.......I.........S--N..-.....  621
NP_001016199   583   -----.--------.--------.-------.......I.........S--N..-.I...  611
XP_001515470   515   -----.--------.--------.-------.......I.........S--N..-.I...  543
XP_426628      576   -----.--------.--------.-------.......I.........S--N..-.I...  604
NP_001083011   593   -----.--------.--------.-------.......I.........S--N..-.....  621
EAW90999       669   -----.--------Y--------.-------.......I.........S--N..-.I...  697
XP_001104796   630   -----.--------Y--------.-------.......I.........S--N..-.I...  658
XP_001104719   610   -----.--------Y--------.-------.......I.........S--N..-.I...  638
NP_071902      630   -----.--------Y--------.-------.......I.........S--N..-.I...  658
EAW91002       429   -----.--------Y--------.-------.......I.........S--N..-.I...  457
AAI33613       634   -----.--------Y--------.-------.......I.........S--N..-.I...  662
BAB15239       405   -----.--------Y--------.-------.......I.........S--N..-.I...  433
NP_001001740   606   -----.--------Y--------.-------.......I.........S--N..-.I...  634
XP_001153038   610   -----.--------Y--------.-------.......I.........S--N..-.I...  638
XP_537181      632   -----.--------Y--------.-------.......I.........S--N..-.I...  660
XP_001493699   511   -----.--------Y--------.-------.......I.........S--N..-.I...  539
EDM09451       632   -----.--------Y--------.-------.......I.........S--N..-.I...  660
NP_036061      632   -----.--------Y--------.-------.......I.........S--N..-.I...  660
XP_993328      572   -----.--------Y--------.-------.......I.........S--N..-.I...  600
XP_001479473   612   -----.--------Y--------.-------.......I.........S--N..-.I...  640
CAH72422       390   -----.--------Y--------.-------.......I.........S--N..-.I...  418
CAH72423       463   -----.--------Y--------.-------.......I.........S--N..-.I...  491
NP_001020297   332   -----.--------Y--------.-------.......I.........S--N..-.I...  360
XP_974586      565   -----.--------Y--------.-------....V..I.....I....--...-.....  593
XP_001373596   639   -----.--------.--------.-------.......I.........S--N..-.I...  667
CAG10910       700   -----.--------.--------.-------.......I.........S--N..-.I...  728
EAW91001       370   -----.--------Y--------.-------.......I.........S--N..-.I...  398
```

```
NP_001071934  548   -----S--------P--------.-------V...R..T.D.........--N..-.I...  576
XP_001183774  209   -----.--------.--------.--------..T.R..I........TS--ND.-.I...  237
XP_001192151  120   -----.--------.--------.--------..T.R..I........TS--ND.-.I...  148
XP_001105376  342   -----S--------Y--------S-------PC.....I......D..S--N..-.K..R  370
XP_636746     875   -----N--------D--------.-------V.T.T..S........TV--N..-.IC..  903
EAX01619      151   -----L--------Y--------.--------.H.....I.........S--R.E-.I...  179
AAA32772      580   -----N--------L--------P-------V.T.R..T........TV--NSE-.L...  608
NP_180854     580   -----N--------L--------P-------V.T.R..T........TV--NSE-.L...  608
CAB89693      580   -----N--------L--------P-------V.T.R..A........TV--SSE-.I...  608
CAB94800      875   -----N--------L--------P-------V.T.R..A........TV--RSE-.I...  903
P93471        577   -----N--------L--------P-------V.T.R..A........TV--RSE-.I...  605
AAG31173      582   -----N--------S--------P-------V.V.R..T........SV--SNE-FIS..  610
AAK81856      567   -----N--------I--------P-------V.T....T........TV--NSE-.I...  595
AAC98912      582   -----N--------L--------P-------V.TLR..T........SV--NNE-FLS..  610
EAY87686      541   -----N--------C--------P-------V.T.R..K........SV--NNE-.I...  569
CAN71084      581   -----N--------L--------P-------V.T.R..T........SV--NSE-.IS..  609
EAZ24764      590   -----N--------C--------P-------V.T.R..K........SV--NNE-.I...  618
NP_001048254  590   -----N--------C--------P-------V.T.R..K........SV--NNE-.I...  618
BAA94422      590   -----N--------C--------P-------V.T.R..K........SV--NNE-.I...  618
BAD16847      509   -----N--------C--------P-------V.T.R..K........SV--NNE-.I...  537
AAK49415      424   -----N--------C--------P-------V.T.R..K........SV--NNE-.I...  452
CAB89694      876   -----N--------L--------P-------V.T.R..A........TV--SSE-.I...  904
BAB02165      743   -----S--------A--------SGINESP-.H..T..T.L......SV--SDG-.I.T.  777
NP_175717     699   -----E--------T--------P--------.H..M..T.V......SV--SDG-.I.T.  727
BAF01052      996   -----TTHGGLSTNA--------.-------SLT.G..T........S.--SDG-.I...
1032
BAF01052      912   -----R--------N--------.--------.GTIRNIA.                        923
NP_192849     932   -----TTHGGLSTNA--------.-------SLT.G..T........S.--SDG-.I...  968
NP_192849     848   -----R--------N--------.--------.GTIRNIA.                        859
NP_182157     925   -----T--------NSSGLSPGA.-------SLTY...T.Q......SV--LDG-.I...  961
BAE99225      925   -----T--------NSSGLSPGA.-------SLTY...T.Q......SV--LDG-.I...  961
AAD23037      428   -----T--------NSSGLSPGA.-------SLTY...T.Q......SV--LDG-.I...  464
BAD94577      137   -----T--------NSSGLSPGA.-------SLTY...T.Q......SV--LDG-.I...  173
NP_001044115  708   -----S--------Q--------ARIIDSP-..T.T..T.T......SI--SDG-.I.T.  742
NP_001056370  1040  -----T--------NSSGLSTDA.-------SMTLS..T........SV--HDG-.IT..
1076
XP_001140892  38    -----L--------Y--------.--------.H.....I.........S--R.E-HI...  66
CAB43046      936   -----TTHGGLSTNA--------.-------SLT.G..T........S.--SDG-.I...  972
NP_683567     735   -----S--------A--------SGINESP-.H..T..T.L......SV--SDG-.I.T.  769
AAC35546      375   -----TTHGGLSTNA--------.-------SLT.G..T........S.--SDG-.I...  411
XP_001130134  38    -----L--------Y--------.--------.H.....I.........S--R.E-.I...  66
EAZ13397      717   -----S--------Q--------ARIIDSP-..T.T..T.T......SI--SDG-.I.T.  751
EAY75677      713   -----S--------Q--------ARIIDSP-.QT.T..T.T......SI--SDG-.I.T.  747
XP_001420221  213   -----T--------A--------.-------V.TYV..T........S.--NA.G.I...  242
CAN60249      127   -----T--------NLDGLSSNA.-------TLT.T..T........SV--LDG-.I...  163
CAL56309      775   -----N--------NPQTA----.-------V.TYT..T........SA--NA.G.I...  808
CAL53135      546   -----G--------D--------L-------TCTL......R.....T.--A.SQHI...  575
CAN64346      476   TSRVLD--------S--------P--------.QT.T..M.V......SI--SDG-.I.T.  509
```

```
EAY99095       962   -----T--------NSSGLSTDA.-------SMTLS..T........SV--HDG-.IT..  998
EAZ35341       962   -----T--------NSSGLSTDA.-------SMTLS..T........SV--HDG-.IT..  998
NP_568435      269   -----G--------R--------V-------I.TYE..V.NR.....SVWRN.A-LFG..  299
AAK64045       144   -----G--------R--------V-------I.TYE..V.NR.....SVWRN.A-LFG..  174
CAA04169       75    -----N--------L--------P-------V.T.R..T....                   89
AAM64761       288   -----R--------R--------V-------V.TYR..V.SR.....SVWRH.G-L.VS.  318
NP_200038      288   -----R--------R--------V-------V.TYR..V.SR.....SVWRH.G-L.VS.  318
NP_487506      1274  -----S--------K--------.-------.HT.Q..T.WV.S.AFNP--..S-ML.S.
1302
NP_487506      1316  -----S--------K--------.-------.HT.Q..TSWVSS.TFSP--..T-ML.S.
1344
NP_487506      1022  -----S--------Q--------.-------FYI.Q..TSCVRS.VFSS--..A-ML.S.
1050
NP_487506      1358  -----G--------E--------.-------.YT.L..T.WVGS.IFSP--..A-IL.S.
1386
NP_487506      1442  -----G--------E--------.-------.YTLH..I.SVRS.AFSS--..L-IL.S.
1470
NP_487506      1148  -----K--------K--------.-------.YTLQ..T.WV.A.AFSP--..A-TL.S.
1176
NP_487506      980   -----G--------E--------.-------.YI.Q..TGWVYS.AFNL--..S-ML.T.
1008
NP_487506      1484  -----G--------E--------.-------IKTL.---S..IYE.M
1499
BAD07933       286   -----.--------A--------AAETAAREV..YS..VSGRS...MGVWRGAG-LI.S.  323
EAY84193       286   -----.--------A--------APETAAREV..YS..VSGRS...MGVWRGAG-LI.S.  323

1_3823         137   SENN-----------ALYIYYKGLSKQVLTF-RFDVVRNI--LEKDKKEED-SNE-FVSA  180
XP_697907      622   ....-----------S..L.......TL...-K..T.KSV--.D.....D.-T..-....  665
NP_001016199   612   ....-----------S..L.......TL...-K..T.KSV--.D..R..D.-T..-....  655
XP_001515470   544   ....-----------S..L.......TL...-K..T.KSV--.D..R..D.-T..-....  587
XP_426628      605   ....-----------S..L.......TL...-K..T.KSV--.D..R..D.-T..-....  648
NP_001083011   622   ....-----------S..L.C.....TL...-K..T.KSV--.D.....D.-T..-....  665
EAW90999       698   ....-----------S..L.......TL...-K..T.KSV--.D..R..D.-T..-....  741
XP_001104796   659   ....-----------S..L.......TL...-K..T.KSV--.D..R..D.-T..-....  702
XP_001104719   639   ....-----------S..L.......TL...-K..T.KSV--.D..R..D.-T..-....  682
NP_071902      659   ....-----------S..L.......TL...-K..T.KSV--.D..R..D.-T..-....  702
EAW91002       458   ....-----------S..L.......TL...-K..T.KSV--.D..R..D.-T..-....  501
AAI33613       663   ....-----------S..L.......TL...-K..T.KSV--.D..R..D.-T..-....  706
BAB15239       434   ....-----------S..L.......TL...-K..T.KSV--.D..R..D.-T..-....  477
NP_001001740   635   ....-----------S..L.......TL...-K..T.KSV--.D..R..D.-T..-....  678
XP_001153038   639   ....-----------S..L.......TL...-K..T.KSV--.D..R..D.-T..-....  682
XP_537181      661   ....-----------S..L.......TL...-K..T.KSV--.D..R..D.-T..-....  704
XP_001493699   540   ....-----------S..L.......TL...-K..T.KSV--.D..R..D.-T..-....  583
EDM09451       661   ....-----------S..L.......TL...-K..T.KSV--.D..R..D.-T..-....  704
NP_036061      661   ....-----------S..L.......TL...-K..T.KSV--.D..R..D.-T..-....  704
XP_993328      601   ....-----------S..L.......TL...-K..T.KSV--.D..R..D.-T..-....  644
XP_001479473   641   ....-----------S..L.......TL...-K..T.KSV--.D..R..D.-T..-....  684
CAH72422       419   ....-----------S..L.......TL...-K..T.KSV--.D..R..D.-T..-....  462
CAH72423       492   ....-----------S..L.......TL...-K..T.KSV--.D..R..D.-T..-....  535
```

```
NP_001020297   361   ...-----------S..L.......TL...-K..T.KSV--.D..R..D.-T..-....  404
XP_974586      594   ...------------.M.........KLFSY-K.EAIQGV--.DQERR.D.-M..-....  637
XP_001373596   668   ...-----------S..L.......TL...-K..T.KSV--.D..R..D.-T..-....  711
CAG10910       729   ...-----------S..L.......TL...-K..T.KSV--.D.....D.-T..-....  772
EAW91001       399   N..-----------S..L.......TL...-K..T.KSV--.D..R..D.-T..-....  442
NP_001071934   577   ...-----------S..........SL..Y-K.N..KSV--.DREQTDD.-.......  620
XP_001183774   238   ...-----------S.FVF.......I...-K..T..SL--M                 266
XP_001192151   149   ...-----------S.FVF.......I...-K..T..SL--M                 177
XP_001105376   371   ...S----------S..L.......TL...-K..A.KSV--.D..Q.GD.-TK.-...T  414
XP_636746      904   ...-----------GV.T...T...PIV.H-..GA--.S--GTGEETDD.-GSQ-...S  945
EAX01619       180   ...-----------S.NLC..A...TL...-K..T.KSV--.D.EG..D.-T..-..G.  223
AAA32772       609   ..T.----------EV.V.H.EITRP.TSH-..GSPD-----MD.AE..A-GSY-.I..  649
NP_180854      609   ..T.----------EV.V.H.EITRP.TSH-..GSPD-----MD.AE..A-GSY-.I..  649
CAB89693       609   ..T.----------EVFV.H.EI..PLTWH-..GTLD-----ME.AED.A-GSY-.I..  649
CAB94800       904   ..T.----------EVFV.H.EI..PLTWH-..GTLD-----ME.AED.A-GSY-.I..  944
P93471         606   ..T.----------EVFV.H.EI..PLTWH-..GTLD-----ME.AED.A-GSY-.I..  646
AAG31173       611   ..T.----------EVFV.H.AI..P.TWH-..GSP------DV.EAD..VTSF-.I..  651
AAK81856       596   ..T.----------EVFV.H.EI..P.TWH-..GSPD-----ME.TEDDA-GSY-.I..  636
AAC98912       611   ..T.----------EVFV.H.AI..P.TWH-..GSP------DI.EAD..-AGSY.I..  651
EAY87686       570   ..T.----------EVFV.H.AI..PAANH-..---VSS--DLD.ADDDP-GSY-.I..  610
CAN71084       610   ..T.----------DVFV.H.EI..P.TWH-K.G---SP--DVD.LDDDV-GPY-.I..  650
EAZ24764       619   ..T.----------EVFV.H.AI..PAANH-..---VSS--DLD.ADDDP-GSY-.I..  659
NP_001048254   619   ..T.----------EVFV.H.AI..PAANH-..---VSS--DLD.ADDDP-GSY-.I..  659
BAA94422       619   ..T.----------EVFV.H.AI..PAANH-..---VSS--DLD.ADDDP-GSY-.I..  659
BAD16847       538   ..T.----------EVFV.H.AI..PAANH-..---VSS--DLD.ADDDP-GSY-.I..  578
AAK49415       453   ..T.----------EVFV.H.AI..PAANH-..---VSS--DLD.ADDDP-GSY-.I..  493
CAB89694       905   ..T.----------EVFV.H----.EP..WH..GTLD-----ME.AED.A-GSY-.I..  942
BAB02165       778   ..T.----------EVFV.H.AFPMP.MSY-M.NNTDSMSG..V.-----.-ASQ-.I.S  819
NP_175717      728   ..T.----------EVFV.H.AFPMP..SY-K.KTIDPV--S.LEV--D.-ASQ-.I.S  769
BAF01052       1033  ..T.----------EV.A.HRS.PMPITSY-K.GSIDP.--SG.E-I...-N.L-...S
1075
NP_192849      969   ..T.----------EV.A.HRS.PMPITSY-K.GSIDP.--SG.E-I...-N.L-...S
1011
NP_182157      962   ..T.----------EV.S...S.PMPMTSY-K.GS.DP.---SGNEYFD.-NGQ-...S
1004
BAE99225       962   ..T.----------EV.S...S.PMPMTSY-K.GS.DP.---SGNEYFD.-NGQ-...S
1004
AAD23037       465   ..T.----------EV.S...S.PMPMTSY-K.GS.DP.---SGNEYFD.-NGQ-...S  507
BAD94577       174   ..T.----------EV.S...S.PMPMTSY-K.GS.DP.---SGNEYFD.-NGQ-...S  216
NP_001044115   743   ..T.----------EVFV.H.AFPMP..AY-K.S.TDP.----SGQEID.-PSQ-.I.C  784
NP_001056370   1077  ...-----------EVFS...TFPMPITSH-K.GSIDP.--TGQETN-D.-NQQ-...S
1119
XP_001140892   67    ...-----------S.NLC..A...TL...-K..T.KSV--.D.EG..D.-T..-..G.  110
CAB43046       973   ..T.----------EV.A.HRS.PMPITSY-K.GSIDP.--SG.E-I...-N.L-...S
1015
NP_683567      770   ..T.----------EVFV.H.AFPMP.MSY-M.NNTDSMSG..V.-----.-ASQ-.I.S  811
AAC35546       412   ..T.----------EV.A.HRS.PMPITSY-K.GSIDP.--SG.E-I...-N.L-...S  454
XP_001130134   67    ...-----------S.NLC..A...TL...-K..T.KSV--.D.EG..D.-T..-..G.  110
```

```
EAZ13397       752   ..T.EEYFGSLTLVRKVFV.H.AFPMP..AY-K.S.TDP.----SGQEID.-PSQ-.I.C  804
EAY75677       748   ..T.EEYFGSLTLVRKVFV.H.AFPMP..AY-K.S.TDP.----SGQEID.-PSQ-.I.C  800
XP_001420221   243   ..D.-----------IVHV.A.HA.SP.AHY-G.ADKPTP--MSHNRR--.-KGG-.I.S  284
CAN60249       164   ..T.-----------EV.T.HRS.PMP.TSH-K.GSIDP.--T.HEIV-D.-NGQ-...S  206
CAL56309       809   ..D.-----------VVHL.A.HS.VP.SSY-S.SDKPAP--VTQHRR--.-KAG-.I.S  850
CAL53135       576   ..T.-----------EVFL.R.D.PMPITSI-S.----------AQERAPE-ERG-.I..  611
CAN64346       510   ..T.----------------------EIVPI-...LIDYV--.TREE.              532
EAY99095       999   ....-----------EVFS...TFPMPITSH-K.GSIDP.--TGQETN-D.-NQQ-...S
1041
EAZ35341       999   ....-----------EVFS...TFPMPITSH-K.GSIDP.--TGQETN-D.-NQQ-...S
1041
NP_568435      300   ....-----------RVFV.DRRWG.P.WVD-G.EP.GMN--SGS..R---------...S  337
AAK64045       175   ....-----------RVFV.DRRWG.P.WVD-G.EP.GMN--SGS..R---------...S  212
AAM64761       319   ....-----------QVFV.D.RWEEP.WVC-GLGHT-.R--FGS.RR---------...S  355
NP_200038      319   ....-----------QVFV.D.RWEEP.WVC-GLGHT-.R--FGS.RR---------...S  355
NP_487506      1303  .GDQ-----------TVRLWEISS..CLH..-Q
1323
NP_487506      1345  .DDQ-----------TVRLWSISSGECLY..
1364
NP_487506      1051  .DDQ-----------TVRLW
1059
NP_487506      1387  .GDQ-----------TVRLW
1395
NP_487506      1471  .DDE-----------TIKLW
1479
NP_487506      1177  .GDQ-----------TVRLW
1185
NP_487506      1009  .GDQ-----------TVRLW
1017
BAD07933       324   ..SG-----------HVFV.DLRW..PIWVH-P.---------------SH-ADA-....  354
EAY84193       324   ..SG-----------HVFV.DLRW..PIWVH-P.---------------SH-ADA-....  354

1_3823         181   VCWR---M-----GS----S---VVVAANSQGTIKXT  202
XP_697907      666   .......---ALPDG-E.----N---.LI.........    689
NP_001016199   656   .......---ALPDG-E.----N---.LI.........    679
XP_001515470   588   .......---ALPDG-E.----N---.LI.........    611
XP_426628      649   .......---ALPDG-E.----N---.LI.........    672
NP_001083011   666   .......---ALPDG-E.----N---.LI.........    689
EAW90999       742   .......---ALPDG-E.----N---.LI.........VS  767
XP_001104796   703   .......---ALPDG-E.----N---.LI.........    726
XP_001104719   683   .......---ALPDG-E.----N---.LI.........    706
NP_071902      703   .......---ALPDG-E.----N---.LI.........    726
EAW91002       502   .......---ALPDG-E.----N---.LI.........    525
AAI33613       707   .......---ALPDG-E.----N---.LI.........    730
BAB15239       478   .......---ALPDG-E.----N---.LI.........    501
NP_001001740   679   .......---ALPDG-E.----N---.LI.........    702
XP_001153038   683   .......---ALPDG-E.----N---.LI.........    706
XP_537181      705   .......---ALPDG-E.----N---.LI.........    728
XP_001493699   584   .......---ALPDG-E.----N---.LI.........    607
```

EDM09451	705	....---ALSDG-E.----N---.LI.........	728
NP_036061	705	....---ALSDG-E.----N---.LI.........	728
XP_993328	645	....---ALSDG-E.----N---.LI.........	668
XP_001479473	685	....---ALSDG-E.----N---.LI.........	708
CAH72422	463	....---ALPDG-E.----N---.LI.........	486
CAH72423	536	....---ALPDG-E.----N---.LI.........	559
NP_001020297	405	....---ALSDG-E.----N---.LI.........	428
XP_974586	638	...K---Q-----N.----N---.........I.	656
XP_001373596	712	....	715
CAG10910	773	....---ALPDG-E.----N---.LI.........	796
EAW91001	443	....	446
NP_001071934	621	.A..---A-----N.----D---IIA.........	640
XP_001105376	415	....---ALPDG-D.----N---.LI.........	438
XP_636746	946	...K---K-----D.----N---ILL......N..	965
EAX01619	224	A...	227
AAA32772	650	...K---S-----D.----P---TMLT........	669
NP_180854	650	...K---S-----D.----P---TMLT........	669
CAB89693	650	...K---S-----DR----P---TILT........	669
CAB94800	945	...K---S-----DR----P---TILT........	964
P93471	647	...K---S-----DR----P---TILT........	666
AAG31173	652	...K---S-----D.----P---TML.........	671
AAK81856	637	...K---S-----D.----P---TMLT........	656
AAC98912	652	...K---S-----D.----P---TML.........	671
EAY87686	611	...K---S-----D.----P---TMLT........	630
CAN71084	651	...K---S-----D.----P---TMLT........	670
EAZ24764	660	...K---S-----D.----P---TMLT........	679
NP_001048254	660	...K---S-----D.----P---TMLT........	679
BAA94422	660	...K---S-----D.----P---TMLT........	679
BAD16847	579	...K---S-----D.----P---TMLT........	598
AAK49415	494	...K---S-----D.----P---TMLT........	513
CAB89694	943	...K---S-----DR----P---TILT........	962
BAB02165	820	I...---G-----Q.----.---TL.....N.N..	839
NP_175717	770	....---G-----Q.----.---TL.....T.N..	789
BAF01052	1076	....---K-----R.----N---M..S.S.N.S..	1095
NP_192849	1012	....---K-----R.----N---M..S.S.N.S..	1031
NP_182157	1005	....---K-----K.----N---ML.....T.NM.	1024
BAE99225	1005	....---K-----K.----N---ML.....T.NM.	1024
AAD23037	508	....---K-----K.----N---ML.....T.NM.	527
BAD94577	217	....---K-----K.----N---ML.....T.NM.	236
NP_001044115	785	....---G-----Q.----.---TLLS...S.N..	804
NP_001056370	1120	....---G-----R.----N---M......T.S..	1139
XP_001140892	111	A...---A-----QPDGEFN---.LI....E....	134
CAB43046	1016	....---K-----R.----N---M..S.S.N.S..	1035
NP_683567	812	I...---G-----Q.----.---TL.....N.N..	831
AAC35546	455	....---K-----R.----N---M..S.S.N.S..	474
XP_001130134	111	A...---A-----QPDGEFN---MLI....E....	134
EAZ13397	805	....---G-----Q.----.---TLLS...S.N..	824
EAY75677	801	....---G-----Q.----.---TLLS...S.N..	820

```
XP_001420221  285   .V.S---P-----N.----K---HLL....R.HL.    304
CAN60249      207   ....---Q-----N.----N---M......S.R..    226
CAL56309      851   .V.S---P-----D.----K---HLL....K.HL.    870
CAL53135      612   CT.K---S-----DD---.---.LIG...N.VVR    631
EAY99095      1042  ....---G-----R.----N---M......T.S..    1061
EAZ35341      1042  ....---G-----R.----N---M......T.S..    1061
NP_568435     338   ....QSGV-----DQ----C---TL..GG.D.VLQ    360
AAK64045      213   ....QSGV-----DQ----C---TL..GG.D.VLQ    235
AAM64761      356   ..L.---Q-----VD----EDWCTL..GG.D.ALE    378
NP_200038     356   ..L.---Q-----VD----EDWCTL..GG.D.ALE    378
BAD07933      355   .A..---QLAGDDSD----G---QL..GG.D.VL.    379
EAY84193      355   .A..---QLAGDDSD----G---QL..GG.D.VL.    379
```
