# Supplementary material for: Global comparative analysis of ESTs from the southern cattle tick, Rhipicephalus (Boophilus) microplus
Source: BMC Genomics. 2007 Oct 12;8:368. doi: 10.1186/1471-2164-8-368 (PMC2100071; doi:10.1186/1471-2164-8-368)
Supplement: Additional file 8 — Alignment from Clustal W for TC12600. This file shows the alignment which was used to generate the phylogenetic tree for TC12600 [file 1471-2164-8-368-S8.pdf]

Alignment view for rid: 8JR0GTHC012, query ID: lcl|1_15640, database: nr

Mouse over the sequence identifer for sequence title

```
1_15640         1     NENVRHM--------NEQVEQYR------S------TSP-YWHQVDLILNQMAG-----L  34
NP_001076460    123     .LQW.--------WQ.M.TH-------T------H..-.....R.S.L.LK------.  153
NP_775813       162     .LEW.--------Q.EM.S-N------P------D..-.....R.T.L.LK------.  192
EAW98053        162     .LEW.--------Q.EM.S-N------P------D..-.....R.T.L.LK------.  192
CAG08547        101     .LQWV--------Q..ITK-Q------P------K..-.....R.A.L.LR------.  131
BAC28089        167     .LEW.--------QREM.-LN------P------D..-.....R.T.L.LK------.  197
BAE37687        167     .LEW.--------QREM.-LN------P------D..-.....R.T.L.LK------.  197
BAE41761        167     .LEW.--------QREM.-LN------P------D..-.....R.T.L.LK------.  197
NP_076114       167     .LEW.--------QREM.-LN------P------D..-.....R.T.L.LK------.  197
BAC26858        161     .LEW.--------QREM.-LN------P------D..-.....R.T.L.LK------.  191
AAH26395        170     .LEW.--------QREM.-LN------P------D..-.....R.T.L.LK------.  200
EDM13780        31      .LEW.--------QREM.-LS------P------D..-.....R.T.L.LK------.  61
BAE41924        167     .LEW.--------QREM.-LN------P------D..-.....R.T.L.LK------.  197
AAG44101        167     .LEW.--------QREM.-LN------P------D..-.....R.T.L.LK------.  197
Q4QQW8          158     .LEW.--------QREM.-LS------P------D..-.....R.T.L.LK------.  188
XP_001104551    162     .LEW.--------Q.EM.S-N------P------D.A-.....R.T.L.LK------.  192
XP_509397       162     .LEW.--------Q.EM.S-N------P------Y.A-.....R.T.L.LK------.  192
XP_534691       358                                         .-H.GE.R.T.L.LK------.  373
BAB23709        181                                        ..-.....R.T.L.LK------.  197
NP_001039635    162     .LEW.--------QKEM.-LN------N------G.A-.....R.T.L.LK------.  192
XP_001490227    120   DT.LEW.--------Q.EM.SNK------D-------.A-.....R.T.L.LK------.  152
CAE69535        128    D.LKW.--------EMEIK.-N------P------EDE-..Q..N.T...LF------.  159
NP_499668       127    D.LKW.--------EQEIRE-N------P------EDE-..Q..N.TV..LF------.  158
NP_510509       155    ..QKWI--------QSSL.TVA------P------DDL-..GA.NRTYH.VS------.  187
EAW98052        162     .LEW.--------Q.EM.S-N------P------D..-.....R.T.L.LK------.  192
CAE63260        123    ..QKWI--------QTTL.TVA------P------DDL-..GA.NRTYH.LS-----M  155
XP_001378519    311     .LAW.--------QK.MASGK------D-------AE-.....E.A.L.LK------.  341
CAE65016        146    Q.LDW.--------RS..QSHP------P-------.D.-F.R..N.TFA.LT-----V  178
NP_497570       146    Q.LDW.--------RS..QANP------P-------.D.-F.R..N.TFA.LT-----I  178
XP_645818       150   ...IAY.--------KQ..AT-N------E------ND.-..INIG.V.T.LS-----M  182
XP_001520206    155                                         .-F.RH.GYV.A.QD------.  170
EDL19769        174     .LEW.--------QREM.-LN------P------D..-.....R.T.L.LK------.  204
BAC32923        167     .LEW.--------QREM.-LN------P------D..-.....R.T.L.LK------.  197
EDL10564        130                                         .-F.RHTGYVVT.LD------.  145
Q8VCI0          158                                         .-F.RHTGYVVT.LD------.  173
BAE42780        59                                          .-F.RHTGYVVT.LD------.  74
XP_979751       157                                         .-F.RHTGYVVT.LD------.  172
NP_080082       158                                         .-F.RHTGYVVT.LD------.  173
XP_416206       145                                         .-F.RHAGY.IA.LD------.  160
XP_543800       213                   KNMKE.K------D-------D.-F.RH.GYVMT..D------.  237
XP_642833       122    ..LQYL--------QTMIDSAP------.------NDL-..QN.ETV.T.ITY-----M  154
NP_001013949    158                                         .-F.RHTGYVVS.LD-----M  173
XP_001497171    131                    KIKD.K------D-------D.-F.RHMGYVMA.LD------.  154
XP_001089478    148                   KNIKA.K------D------D.--F.RHTGYVMA.ID------.  172
```

```
CAF90435        101                  ...KLSK------.------SD.-L.RH.GF.IA..D.-----.  126
CAC13971        125                  .NIKY.K------.-------D.-F.RHA.YVMA..D.-----.  149
XP_614819       145                  .NIKY.K------.-------D.-F.RHA.YVMA..D.-----.  169
XP_001157500    101                  KNIKE.K------T------D.--F.RHTGYVMA.ID.-----.  125
XP_520758       149                  KNIKE.K------T------D.--F.RHTGYVMA.ID.-----.  173
XP_637940       131   ..ILY.--------.Q.I-.LN------Q------ND.-..IN.N.V.Q.LN.-----.  162
NP_079105       148                  KNIKE.K------T------D.--F.RHTGYVMA.ID.-----.  172
Q6P4A8          147                  KNIKE.K------T------D.--F.RHTGYVMA.ID.-----.  171
BAB15442        101                  KNIKE.K------T------D.--F.RHTGYVMA.ID.-----.  125
EAW96323        35                   KNIKE.K------T------D.--F.RHTGYVMA.ID.-----.  59
EAW96322        148                  KNIKE.K------T------D.--F.RHTGYVMA.ID.-----.  172
XP_698232       120      .F.--------AK.D.WS.LQVKRNT------.D.-L.IHTG...A.LD.-----.  155
XP_647502       159  .TQMEFV--------RD..FENN------G------S.Q-..YSTG..MS.FD.-----.  192
XP_001370772    35                   ...GKNK------K-------..-F.RHIGY..S.LD.-----.  59
XP_638577       140   ..IESI--------HTFIVNN.------K-------.R-..N.IT.VMD.IN.M----.  172
XP_699488       115                   ..KLNG------K------SD.-L..H.G.LVA..D.-----.  139
XP_642656       129  SD.WEY.--------M.R.NSSS------T------.D.-..I.IRNAMS.QI.-----.  162
XP_793919       160                                        .-F.EG.G.V.A.FE.-----.  175
AAC31917        1                                    S.Q-..YSTG..MS.FD.-----.  18
XP_643736       147                        .------D------E.V-.DT..SNVIY.FE.-----F  166
XP_844857       155   .H..Y.--------D--AAKFE------.------SAF-.TQ----LR.ML.L-----I  181
AAC28456        155   .H..Y.--------D--AAKFE------.------SAF-.TQ----LR.ML.L-----I  181
XP_844855       155   .H..Y.--------D--AAKFE------.------SAF-.TQ----LR.ML.L-----I  181
XP_001454659    172  ..LSQYL--------V..Y.YS.------QLVKDNKSDE-.YEALAR..A.ND.-----.  211
XP_655293       122                               N------STQ-..KEQN..FK.FR.-----.  140
XP_650973       105  .K.I.RFMSLQFNFWRKHIKNNE------N------DDV-..KNQK..FE.FR.-----.  146
XP_001024340    126                   .LQ.NN------Q------..QG..GL.GSLMS.LN.TYDGYV  156
XP_649424       102  .TLINK.--------Q..LDFFK------TESSTN-.NE-..RRQKV.KY.FN.-----.  140
XP_001453083    105                     ..SQ------.Q-----NT.-..RM.KYL.I.LEN-----M  128

1_15640         35   ---------D----DA--RRGHTQY-F-----PSWSY-VNAT------DLL------FLN  60
NP_001076460    154  ---------E----.G--YN.RID--.-----..G.FSI.PF------GF.------LFQ  179
NP_775813       193  ---------E----.S--YE.RVS--.-----.AGKFTIKPL------GF.------L.Q  218
EAW98053        193  ---------E----.S--YE.RVS--.-----.AGKFTIKPL------GF.------L.Q  218
CAG08547        132  ---------E----.S--YNDQLSF-P-----TGPIS-F.PF------GFI------LFQ  157
BAC28089        198  ---------E----.S--YE.RLT--.-----.TGRFTIKPL------GF.------L.Q  223
BAE37687        198  ---------E----.S--YE.RLT--.-----.TGRFTIKPL------GF.------L.Q  223
BAE41761        198  ---------E----.S--YE.RLT--.-----.TGRFTIKPL------GF.------L.Q  223
NP_076114       198  ---------E----.S--YE.RLT--.-----.TGRFTIKPL------GF.------L.Q  223
BAC26858        192  ---------E----.S--YE.RLT--.-----.TGRFTIKPL------GF.------L.Q  217
AAH26395        201  ---------E----.S--YE.RLT--.-----.TGRFTIKPL------GF.------L.Q  226
EDM13780        62   ---------E----.S--YE.RLT--.-----.TGRFNIKPL------GF.------L.Q  87
BAE41924        198  ---------E----.S--YE.RLT--.-----.TGRFTIKPL------GF.------L.Q  223
AAG44101        198  ---------E----.S--YE.RLT--.-----.TGRFTIKPL------GF.------L.Q  223
Q4QQW8          189  ---------E----.S--YE.RLT--.-----.TGRFNIKPL------GF.------L.Q  214
XP_001104551    193  ---------E----.S--YE.RVS--.-----.AGKFTIKPL------GF.------L.Q  218
XP_509397       193  ---------E----.S--YE.RVS--.-----.AGKFTIKPL------GF.------L.Q  218
```

```
XP_534691      374  ---------E----.S--YE.-SMT-.------.TGRFTIKPL------GF.------L.Q  399
BAB23709       198  ---------E----.S--YE.RLT--.------.TGRFTIKPL------GF.------L.H  223
NP_001039635   193  ---------E----.S--YE.SVA--.------.TGKFT.KPL------GF.------L.Q  218
XP_001490227   153  ---------E----.S--YE.SVA--.------.TGRFTIKPL------GF.------L.Q  178
CAE69535       160  ---------I----HG--YENQLGA-P-----INFKE-IAVH------PIF------MIQ  185
NP_499668      159  ---------I----HG--YENQLGA-E-----IDFKQ-IAVH------PIF------MIQ  184
NP_510509      188  ---------I----..--YE.R-EF-K-----.RIT.--ELH------PI.------Y..  211
EAW98052       193  ---------E----.S--YE.RVS--.------.AGKFTIKPL------GF.------L.Q  218
CAE63260       156  ---------I----..--YE.R-EF-K-----.RVT.--ELH------PI.------Y..  179
XP_001378519   342  ---------E----.S--YQ.RIA--.------..KNFTITPF------GF.------LFQ  367
CAE65016       179  ---------Y----..YSK.NL.---------.EIGF--DLH------PIY------MMQ  202
NP_497570      179  ---------Y----..YSK.NL.---------.EIGF--DLH------PIY------MMQ  202
XP_645818      183  ---------V----.G--YNAAN.D-------..-RQ-LSFL------.FI------LI.  206
XP_001520206   171  ---------A----AG--AEEWANV-T-----HAKQP-.TRF------QVQ------...  196
EDL19769       205  ---------E----.S--YE.RLT--.------.TGRFTIKPL------GF.------L.Q  230
BAC32923       198  ---------E----.S--YE.RLT--.------.TGRFTIKPL------GF.------L.Q  223
EDL10564       146  YLG------A----QK--.ASEEKI-K----.-----MTMF------QIQ------...  170
Q8VCI0         174  YLG------A----QK--.ASEEKI-K----.-----MTMF------QIQ------...  198
BAE42780       75   YLG------A----QK--.ASEEKI-K----.-----MTMF------QIQ------...  99
XP_979751      173  YLG------A----QK--.ASEEKI-K----.-----MTMF------QIQ------...  197
NP_080082      174  YLG------A----QK--.ASEEKI-K----.-----MTMF------QIQ------...  198
XP_416206      161  ---------YMGALEW--AKL.K.----------TP-LSVF------.VQ------...  185
XP_543800      238  YV-------G----AM--..AMLEK-T-----KPMTL-FQ---------IQ------...  262
XP_642833      155  ------------------Q..YN.S-VIDNGVDASQS-LGI.------EFF------LM.  182
NP_001013949   174  YLG------A----QK--.ASEEEM-K----.-----MTMF------QIQ------...  198
XP_001497171   155  YV-------G----AM--K.ASLEG-I-----KPMTM-FQ---------IQ------...  179
EDM01610       8                                                              ...  10
XP_001089478   173  YV-------G----AK--..A---I-L-----EGTKP-MTLF------QIQ------...  197
CAF90435       127  ---------Q----AG---------L-----AD.AK-TKGKKPLSMF.IQ------...  151
CAC13971       150  ---------F-----.--GATKRAV-L-----EGKKP-MTLF------QIQ------...  174
XP_614819      170  ---------F-----.--GATKRAV-L-----EGKKP-MTLF------QIQ------...  194
XP_001157500   126  ---------Y----VG--AKKRA-I-L-----EGTKP-MTLF------QIQ------...  150
XP_520758      174  ---------Y----VG--AKKRA-I-L-----EGTKP-MTLF------QIQ------...  198
XP_637940      163  ---------T----NG--YSDAN.N-------.D-RQ-LSLM------.FI------L..  186
NP_079105      173  ---------Y----VG--AKKRA-I-L-----EGTKP-MTLF------QIQ------...  197
Q6P4A8         172  ---------Y----VG--AKKRA-I-L-----EGTKP-MTLF------QIQ------...  196
BAB15442       126  ---------Y----VG--AKKRA-I-L-----EGTKP-MTLF------QIQ------...  150
EAW96323       60   ---------Y----VG--AKKRA-I-L-----EGTKP-MTLF------QIQ------...  84
EAW96322       173  ---------Y----VG--AKKRA-I-L-----EGTKP-MTLF------QIQ------...  197
XP_698232      156  ------------------QA.VN.----------.AK-QHGR------KP.SQFAAQ...  180
XP_647502      193  ---------V----N-----.YQ.SP.-----.QL.E-IQ---------.Y------I.T  213
XP_001370772   60   ---------Y----FG--AAQRAKA-A-----QRKP--LTVF------QVQ------...  84
XP_638577      173  ---------.----GY--NEAN.N---------.SET-LSLH------.FF------V..  195
XP_699488      140  -------------------HAGAEF-W-----AKSRQ-KKPL------SMFAVQ---L..  164
XP_642656      163  YEGYNAAAGE----.Y--QKTFIEI-Y-----MINL.-GDMG------.IV------T.T  197
XP_793919      176  ---------I----KG--YE-MS.F-S-----NASTS-NGFL------AMQ------V..  200
AAC31917       19   ---------V----N-----.YQ.SP.-----.QL.E-IQ---------.Y------I.T  39
```

```
XP_643736       167  ------------------A..YQ.A-A-----D.DKQ-LTTL------Q..------L.Q  189
XP_844857       182  ---------.----GM--VA.YNAR-A-----.ADER-LDRM------K..------MY.  207
AAC28456        182  ---------.----GM--VA.YNAR-A-----.ADER-LDRM------K..------MY.  207
XP_844855       182  ---------.----GM--VA.YNAR-A-----.ADER-LDRM------K..------MY.  207
XP_001454659    212  ---------F----Q.--IQDKAPV-N-----KRLTW-.Q---------..------L.Q  234
XP_655293       141  ------------------LS.YNK.-C-----DNGKK-LSIL------EMY------YI.  163
XP_650973       147  ---------W----NG--YKKFGK-----------EP-MTLE------E.Y------L..  167
XP_001024340    157  ---------.----AM--.KNQQ.Q----------NI-LSFN------QFY------Y.T  178
XP_649424       141  ---------V----.-----.YMAA-S-----.AEEE-LPEI------E.Y------M.L  163
XP_001453083    129  ---------Y----EG--YNY.--.-G-----KTEKA-LSFD------QFY------Y.S  152

1_15640         61   ADGDLEDL-----------EGA-L------------K-----------R-----RVG---  77
NP_001076460    180  MG......-----------.A.-.------------N-----------K-----SSQ---  196
NP_775813       219  LS......-----------.L.-.------------N-----------K-----TKI---  235
EAW98053        219  LS......-----------.L.-.------------N-----------K-----TKI---  235
CAG08547        158  LG......-----------.S.-.------------N-----------K-----SSQ---  174
BAC28089        224  IS......-----------.P.-.------------N-----------K-----TNT---  240
BAE37687        224  IS......-----------.P.-.------------N-----------K-----TNT---  240
BAE41761        224  IS......-----------.P.-.------------N-----------K-----TNT---  240
NP_076114       224  IS......-----------.P.-.------------N-----------K-----TNT---  240
BAC26858        218  IS......-----------.P.-.------------N-----------K-----TNT---  234
AAH26395        227  IS......-----------.P.-.------------N-----------K-----TNT---  243
EDM13780        88   IS......-----------.P.-.------------N-----------K-----TNT---  104
BAE41924        224  IS......-----------.P.-.------------N-----------K-----TNT---  240
AAG44101        224  IS......-----------.P.-.------------N-----------K-----TNT---  240
Q4QQW8          215  IS......-----------.P.-.------------N-----------K-----TNT---  231
XP_001104551    219  LS......-----------.L.-.------------N-----------K-----TKI---  235
XP_509397       219  LS......-----------.L.-.------------N-----------K-----TKI---  235
XP_534691       400  LS...G..-----------.P.-.------------N-----------K-----TKT---  416
BAB23709        224  IS.Y..N.-----------KP.-.------------N-----------K-----TNT---  240
NP_001039635    219  IS......-----------.V.-.------------N-----------K-----TKT---  235
XP_001490227    179  IS...G..-----------.P.-.------------N-----------K-----TEA---  195
CAE69535        186  IA......-----------ALK-F------------.-----------K-----PEN---  202
NP_499668       185  IA......-----------AMK-F------------.-----------K-----PEN---  201
NP_510509       212  LN..FY..-----------.KK-.N-----------.-----------T-----.DP---  229
EAW98052        219  LS......-----------.L.-.------------N-----------K-----TKI---  235
CAE63260        180  LN..FY..-----------.KK-.N-----------.-----------T-----.DP---  197
XP_001378519    368  LS......-----------.P.-.------------N-----------K-----TAH---  384
CAE65016        203  LA..MF..-----------NKY-.------------N-----------K-----TPD---  219
NP_497570       203  LA..MF..-----------NKL-.------------N-----------K-----TADP--  220
XP_645818       207  M.A..G.I-----------SST-F------------NQSTSFSEISNFK-----KSM---  234
XP_001520206    197  .V...L..-----------IP.-.------------F-----------P-----.RA---  213
EDL19769        231  IS......-----------.P.-.------------N-----------K-----TNT---  247
BAC32923        224  IS......-----------.P.-.------------N-----------K-----TNT---  240
EDL10564        171  .V...L..-----------IPS-.------------SPTKSS------S-----MMK---  192
Q8VCI0          199  .V...L..-----------IPS-.------------SPTKSS------S-----MMK---  220
BAE42780        100  .V...L..-----------IPS-.------------SPTKSS------S-----MMK---  121
```

```
XP_979751      198  .V...L..-----------IPS-.------------SPTKSS------S-----MMK---  219
NP_080082      199  .V...L..-----------IPS-.------------SPTKSS------S-----MMK---  220
XP_416206      186  .V...L..-----------IP.-.FEY---------S-----------A-----.S.---  205
XP_543800      263  .V...L..-----------IPS-V------------SPT---------K-----NSS---  281
XP_642833      183  M...MI..-----------GP.-.------------N-----------L-----TN.---  199
NP_001013949   199  .V...L..-----------IPS-.------------S-----------P-----TKS---  215
XP_001497171   180  .V...L..-----------IPS-.------------S-----------P-----TQV---  196
EDM01610       11   .V...L..-----------IPS-.------------S-----------P-----TKS---  27
XP_001089478   198  .V...L..-----------IPS-.------------S-----------P-----TKN---  214
CAF90435       152  .V...L..-----------IP.-.------------A-----------P-----SS.PLP  171
CAC13971       175  .I...L..-----------IPS-.------------S-----------P-----TKN---  191
XP_614819      195  .I...L..-----------IPS-.------------S-----------P-----TKN---  211
XP_001157500   151  SV...L..-----------IPS-.------------SPT---------K-----NGS---  169
XP_520758      199  SV...L..-----------IPS-.------------SPT---------K-----NGS---  217
XP_637940      187  MNVEIY.I-----------MNS-.------------.-----------N-----NSSSFY  206
NP_079105      198  SV...L..-----------IPS-.------------SPT---------K-----NGS---  216
Q6P4A8         197  SV...L..-----------IPS-.------------SPT---------K-----NGS---  215
BAB15442       151  SV...L..-----------IPS-.------------SPT---------K-----NGS---  169
EAW96323       85   SV...L..-----------IPS-.------------SPT---------K-----NGS---  103
EAW96322       198  SV...L..-----------IPS-.------------SPT---------K-----NGS---  216
XP_698232      181  .V...L..-----------IP.-.------------S-----------PGESTV.HR---  202
XP_647502      214  SA....T.VTLFPSSSSSSSST-T------------N-----------E-----NKN---  241
XP_001370772   85   .I..TL..-----------LPS-.------------MVPGNV------S-----LDH---  106
XP_638577      196  MF...F..-----------MP.-.------------N-----------L-----DKE---  212
XP_699488      165  GI...L..-----------IPV-.------------.-----------.-----HSN---  181
XP_642656      198  TTPNN.FI-----------PMD--------------R-----------K-----E.E---  213
XP_793919      201  SC...L..-----------KS.-VMPSLIPDWDKLT.-----------K-----EFL---  229
AAC31917       40   SA....T.-----------VTL-F------------P-----------S-----SSS---  56
XP_643736      190  YA.....V-----------A.Y-.------------E-----------Y-----EMA---  206
XP_844857      208  MQAEIG.I-----------VR.-T------------SPAEVLDDM---.-----QMM---  232
AAC28456       208  MQAEIG.I-----------VR.-T------------SPAEVLDDM---.-----QMM---  232
XP_844855      208  MQAEIG.I-----------VR.-T------------SPAEVLDDM---.-----QMM---  232
XP_001454659   235  .C..IYE.-----------KE.-M------------.-----------P-----KAE---  251
XP_655293      164  .G...---------------.T-.------------N-----------K-----L.K---  176
XP_650973      168  SV...PTI-----------TDK-F------------E-----------K-----IHS---  184
XP_001024340   179  NM.....I-----------QN.V.------------.-----------T-----NKT---  196
XP_649424      164  SV....N.-----------NEI-Y------------.------------------T.---  178
XP_001453083   153  NM...Q..-----------WPS-V------------G-----------Q-----V-----  167

1_15640        78   -----R-------------KVL--G-----------------S-------------G---  84
NP_001076460   197  -----S-------------RSV--.-----------------.-------------.---  203
NP_775813      236  -----K-------------PS.--.-----------------.-------------.---  242
EAW98053       236  -----K-------------PS.--.-----------------.-------------.---  242
CAG08547       175  -----T-------------RP.--.-----------------.-------------.---  181
BAC28089       241  -----K-------------PS.--.-----------------.-------------.---  247
BAE37687       241  -----K-------------PS.--.-----------------.-------------.---  247
BAE41761       241  -----K-------------PS.--.-----------------.-------------.---  247
```

```
NP_076114       241  -----K-------------PS.--.-----------------.-------------.---  247
BAC26858        235  -----K-------------PS.--.-----------------.-------------.---  241
AAH26395        244  -----K-------------PS.--.-----------------.-------------.---  250
EDM13780        105  -----K-------------PSV--.-----------------.-------------.---  111
BAE41924        241  -----K-------------PS.--.-----------------.-------------.---  247
AAG44101        241  -----K-------------PS.--.-----------------.-------------.---  247
Q4QQW8          232  -----K-------------PSV--.-----------------.-------------.---  238
XP_001104551    236  -----K-------------PS.--.-----------------.-------------.---  242
XP_509397       236  -----K-------------PS.--.-----------------.-------------.---  242
XP_534691       417  -----K-------------HIM--.-----------------.-------------.---  423
BAB23709        241  -----K-------------PS.--.-----------------.-------------.---  247
NP_001039635    236  -----N-------------HAM--.-----------------.-------------.---  242
XP_001490227    196  -----.-------------RT.--.-----------------A-------------.---  202
CAE69535        203  -----P-------------.KVFS.-----------------P-------------.---  211
NP_499668       202  -----P-------------.KVFS.-----------------P-------------.---  210
NP_510509       230  -----A-------------FEQ--T-----------------G-------------.---  236
AAH97934        7                         V--.-----------------.-------------.---  10
EAW98052        236  -----K-------------PS.--.-----------------.-------------.---  242
CAE63260        198  -----A-------------LDQ--T-----------------G-------------.---  204
XP_001378519    385  -----.-------------R.M--.-----------------.-------------.---  391
CAE65016        220  -----PL------------EYP--E-----------------A-------------.---  227
NP_497570       221  -----M-------------EYP--E-----------------G-------------.---  227
XP_645818       235  -----D-------------HIK--K-----------------T-------------D---  241
XP_001520206    214  -----E-------------SW.--H-----------------AAPGPSGAWDM---.---  230
EDL19769        248  -----K-------------PS.--.-----------------.-------------.---  254
BAC32923        241  -----K-------------PS.--.-----------------.-------------.---  247
EDL10564        193  -----F-------------.IW--E-----------------M-------------.---  199
Q8VCI0          221  -----F-------------.IW--E-----------------M-------------.---  227
BAE42780        122  -----F-------------.IW--E-----------------M-------------.---  128
XP_979751       220  -----F-------------.IW--E-----------------M-------------.---  226
NP_080082       221  -----F-------------.IW--E-----------------M-------------.---  227
XP_416206       206  -----Q-------------CNV--E-----------------A-------------.---  212
XP_543800       282  -----L-------------..F--K-----------------RWDM----------.---  291
XP_642833       200  -----K-------------Q.T--SPATATSPKQAFKEFMRRT-------------.---  223
NP_001013949    216  -----SSLKKF--------.IW--E-----------------M-------------.---  227
XP_001497171    197  -----YNLKFF--------.RW--D-----------------M-------------.---  208
EDM01610        28   -----SSLKKF--------.IW--E-----------------M-------------.---  39
XP_001089478    215  -----SSLKFF--------.RW--D-----------------M-------------.---  226
CAF90435        172  R----H-------------.LP--.-----------------M-------------.---  179
CAC13971        192  -----SSLKFF--------.RW--D-----------------M-------------.---  203
XP_614819       212  -----SSLKFF--------.RW--D-----------------M-------------.---  223
XP_001157500    170  -----L-------------..F--K-----------------RWDM----------.---  179
XP_520758       218  -----L-------------..F--K-----------------RWDM----------.---  227
XP_637940       207  QQPNNN-------------SFD--N-----------------N-------------Q---  218
NP_079105       217  -----L-------------..F--K-----------------RWDM----------.---  226
Q6P4A8          216  -----L-------------..F--K-----------------RWDM----------.---  225
BAB15442        170  -----L-------------..F--K-----------------RWDM----------.---  179
```

```
EAW96323       104  -----L-------------..F--K-----------------RWDM----------.---  113
EAW96322       217  -----L-------------..F--K-----------------RWDM----------.---  226
XP_698232      203  -----Y-------------PDI--P-----------------M-------------.---  209
XP_647502      242  -----K-------------NIK--I-----------------.-------------SIKP  251
XP_001370772   107  -----S-------------E.D--E-----------------M-------------.---  113
XP_638577      213  -----Y-------------.YF--QKDLNDIQDWFKR-----.-------------Q---  231
XP_699488      182  -----SSQDSF--------RMP--.-----------------M-------------S---  193
XP_642656      214  -----Q-------------LMA--T-----------------T-------------.---  220
XP_793919      230  -----K-------------FIR--T-----------------.-------------.---  236
AAC31917       57   -----S-------------SSS--T-----------------T-------------N---  63
XP_643736      207  -----QNKTEYINRV----.S.--K-----------------EIETLFAVK-----.---  230
XP_844857      233  -----P-------------RWF--V-----------------D-------------T---  239
AAC28456       233  -----P-------------RWF--V-----------------D-------------T---  239
XP_844855      233  -----P-------------RWF--V-----------------D-------------T---  239
XP_001454659   252  -----.IQWHLLTPEEFEWERE--K-----------------R-------------I---  271
XP_655293      177  -----N-------------DN.--P-----------------K-------------Y---  183
XP_650973      185  -----E-------------NSM--L-----------------DLQDKNPDELY---H---  201
XP_001024340   197  -----E-------------.----------------------------------------  198
XP_649424      179  -----K-------------EPE--T-----------------.IVYMRKAFPDRIYH---  198
XP_001453083   168  -------------------QTQ--.-----------------I-------------.---  173

1_15640        85   -------S---------------------CSALVKVLPGNRDIYFSQVTWSTY-ASMLRI  115
NP_001076460   204  -------.---------------------....I.L...HK.LLV.HD..NN.-Q.....  234
NP_775813      243  -------.---------------------....I.L...QS.LLVAHN..NN.-QH...V  273
EAW98053       243  -------.---------------------....I.L...QS.LLVAHN..NN.-QH...V  273
CAG08547       182  -------.---------------------....I.L..NHK.LLV.HD..N..-Q.....  212
BAC28089       248  -------.---------------------....I.L...GH.LLVAHN..NS.-QN....  278
BAE37687       248  -------.---------------------....I.L...GH.LLVAHN..NS.-QN....  278
BAE41761       248  -------.---------------------....I.L...GH.LLVAHN..NS.-QN....  278
NP_076114      248  -------.---------------------....I.L...GH.LLVAHN..NS.-QN....  278
BAC26858       242  -------.---------------------....I.L...GH.LLVAHN..NS.-QN....  272
AAH26395       251  -------.---------------------....I.L...GH.LLVAHN..NS.-QN....  281
EDM13780       112  -------.---------------------....I.L...SH.LLVAHN..NS.-QN....  142
BAE41924       248  -------.---------------------....I.L...GH.LLVAHN..NS.-QN....  278
AAG44101       248  -------.---------------------....I.L...GH.LLVAHN..NS.-QN....  278
Q4QQW8         239  -------.---------------------....I.L...SH.LLVAHN..NS.-QN....  269
XP_001104551   243  -------.---------------------....I.L...QS.LLVAHN..NN.-QH...V  273
XP_509397      243  -------.---------------------....I.L...QS.LLVAHN..NN.-QH...V  273
XP_534691      424  -------.---------------------....I.L...QS.LLIAHN...S.-QN....  454
BAB23709       248  -------.---------------------....I.L...GH.LLVAHN..NS.-QN....  278
NP_001039635   243  -------.---------------------....I.L...Q..LLVAHN..HS.-QY....  273
XP_001490227   203  -------A---------------------......L...H..LLVAHN..NS.-Q.....  233
CAE69535       212  -------H---------------------......L..N.E..L..H....S.-GT....  242
NP_499668      211  -------H---------------------......L..K.E..L..H....S.-GT....  241
NP_510509      237  -------K---------------------..G.I..A...A.LFI....M.GF-QN...V  267
AAH97934       11   -------.---------------------....I.L...SH.LLVAHN..NS.-QN....  41
EAW98052       243  -------.---------------------....I.L...QS.LLVAHN..NN.-QH...V  273
```

```
CAE63260       205   -------K---------------------..G.I..A...A.LFI....M.GF-QN....   235
XP_001378519   392   -------.---------------------....I.L..NRKELLV.HD...S.-QH....   422
AAI26079       1                                                         .-QN....   7
CAE65016       228   -------R---------------------..GFI.LA...K.LFIAH.SM.SL-SW.Q..   258
NP_497570      228   -------R---------------------..GF..LA...K.MFMAH.SM.SL-SW.Q.V   258
XP_645818      242   -------H---------------------..G.I.LTDDLTEL.SAHTS..S.-IN....   272
XP_001520206   231   -------H---------------------....I.....YENV.LAHSS.FS.-.A...V   261
EDL19769       255   -------.---------------------....I.L...GH.LLVAHN..NS.-QN....   285
BAC32923       248   -------.---------------------....I.L...GH.LLVAHN..NS.-QN....   278
EDL10564       200   -------H---------------------....I.....FEN...AHSS.Y..-.A....   230
Q8VCI0         228   -------H---------------------....I.....FEN...AHSS.Y..-.A....   258
BAE42780       129   -------H---------------------....I.....FEN...AHSS.Y..-.A....   159
XP_979751      227   -------H---------------------....I.....FEN...AHSS.Y..-.A....   257
NP_080082      228   -------H---------------------....I.....FEN...AHSS.Y..-.A....   258
XP_416206      213   -------GHGKYQWDMGH-----------....I.....YEN...AHSS.F..-.AT...   253
XP_543800      292   -------H---------------------....I.....FEN.F.AHSS.Y..-.A....   322
XP_642833      224   -------H---------------------....I.MTDDLS.LFSGHT...S.-YE.V.M   254
NP_001013949   228   -------H---------------------....I.....FEN...AHSS.Y..-.A....   258
XP_001497171   209   -------H---------------------....I.....FEN.F.AHSS.Y..-.A....   239
EDM01610       40    -------H---------------------....I.....FEN...AHSS.Y..-.A....   70
XP_001089478   227   -------H---------------------....I.....FEN.L.AHSS.Y..-.A....   257
CAF90435       180   -------H---------------------....I.....FENLL.AHSS.Y..-.ATM..   210
AAW25845       1                                                             ...V   4
CAC13971       204   -------H---------------------....I.....FEN.F.AHSS.Y..-.A....   234
XP_614819      224   -------H---------------------....I.....FEN.F.AHSS.Y..-.A....   254
XP_001157500   180   -------H---------------------....I.....FEN.L.AHSS.Y..-.A....   210
XP_520758      228   -------H---------------------....I.....FEN.L.AHSS.Y..-.A....   258
XP_637940      219   -------H---------------------....I.LTDDLTEL.TGHT...D.-YQ.V.M   249
NP_079105      227   -------H---------------------....I.....FEN.L.AHSS.Y..-.A....   257
Q6P4A8         226   -------H---------------------....I.....FEN.L.AHSS.Y..-.A....   256
BAB15442       180   -------H---------------------....I.....FENVL.AHSS.Y..-.A....   210
EAW96323       114   -------H---------------------....I.....FEN.L.AHSS.Y..-.A....   144
EAW96322       227   -------H---------------------....I.....FEN.L.AHSS.Y..-.A....   257
XP_698232      210   -------H---------------------....I.M...FENLL.AHSS.Y..-.ATM..   240
XP_647502      252   NFKDELTD---------------------..GFIRI..DYS.V..GHT..RY.-YAL...   289
XP_001370772   114   -------H---------------------..V.I.....FEN.L..HSS.FS.-..T...   144
XP_638577      232   -------H---------------------....I..SSDYSEL.SGHT...G.-YT....   262
XP_803464      238                                .......T--EY.....HA...SF-N....Q   265
XP_699488      194   -------H---------------------....I.M...YENLLLGHSS.Y..-.ASM..   224
XP_642656      221   -------H---------------------.TSII.LTNNCS.LMSAHTS.ADF-SV.I..   251
XP_793919      237   -------H---------------------......ICA-------AL.KVGRF-.PPFQS   260
AAC31917       64    -------ENKNKNIKISSIKPNFKDELTD..GFIRI..DYS.V..GHT..RY.-YAL...   115
XP_643736      231   -------R---------------------..G..RIT.DYGELFI.HT..GS.FTAGY..   262
XP_844857      240   -------H---------------------...F...V--KD....GHA...SF-NT...Q   268
AAC28456       240   -------H---------------------...F...V--KD....GHA...SF-NT...Q   268
XP_844855      240   -------H---------------------...F...V--KD....GHA...SF-NT...Q   268
XP_001454659   272   -------.---------------------..S.I.A.DDWS.VWMAHT..TS.-QN....   302
```

```
XP_655293       184  -------.TINMEKMNQHEIFHE------.T.I..I..Q.Q..V..HN..RP.-YA.M..  229
XP_650973       202  -------E---------------------...Y..YNNKSH..I..HN..RP.-YA....  232
XP_001024340    199  -------N---------------------.D.Y..LT--HN.LVCAHS.FNI.-SL...V  227
XP_649424       199  -------E---------------------.....RAV--GDEV..AHG..RG.-YA....  227
XP_001453083    174  -------N---------------------.NSFY.--..----IVAHS.FNV.-QT....  198

1_15640         116  LKK--YSL--RFHK--T-FE-ST--E--LIPGHTLT-FSSAPGRIFSGDDFYLI-S---S  158
NP_001076460    235  M.R--...--SYRT--S-PT-EQ--D--V...A.QV-...Y..S..........-.---.  277
NP_775813       274  I..--.W.--Q.REG-P-WG-DY--P--.V..NK.V-...Y..T...C....IL-G---.  317
EAW98053        274  I..--.W.--Q.REG-P-WG-DY--P--.V..NK.V-...Y..T...C....IL-G---.  317
CAG08547        213  I.R--.NF--E.QV--S-PI-DN--D--PL..GIQA-...Y..S........IL-.---.  255
BAC28089        279  I..--.R.--Q.REG-P-Q.-EY--P--.VA.NN.V-...Y..T........IL-G---.  322
BAE37687        279  I..--.R.--Q.REG-P-Q.-EY--P--.VA.NN.V-...Y..T........IL-G---.  322
BAE41761        279  I..--.R.--Q.REG-P-Q.-EY--P--.VA.NN.V-...Y..T........IL-G---.  322
NP_076114       279  I..--.R.--Q.REG-P-Q.-EY--P--.VA.NN.V-...Y..T........IL-G---.  322
BAC26858        273  I..--.R.--Q.REG-P-Q.-EY--P--.VA.NN.V-...Y..T........IL-G---.  316
AAH26395        282  I..--.R.--Q.REG-P-Q.-EY--P--.VA.NN.V-...Y..T........IL-G---.  325
EDM13780        143  I..--.R.--Q.REG-P-Q.-EY--P--..A.NN.I-...Y..T........IL-G---.  186
BAE41924        279  I..--.R.--Q.REG-P-Q.-EY--P--.VA.NN.V-...Y..T........IL-G---.  322
AAG44101        279  I..--.R.--Q.REG-P-Q.-EY--P--.VA.NN.V-...Y..T........IL-G---.  322
Q4QQW8          270  I..--.R.--Q.REG-P-Q.-EY--P--..A.NN.I-...Y..T........IL-G---.  313
XP_001104551    274  I..--.W.--Q.RE--G-PQGDS--P--.V..NK.V-...Y..T...C....IL-G---.  317
XP_509397       274  I..--.W.--Q.RE--G-PR-GDY-P--.V..NK.V-...Y..T...C....IL-G---.  317
XP_534691       455  I..--.WF--Q.RE--DPQ.-NS--P--.A..NK.V-...Y..TL..C....IM-G---.  498
BAB23709        279  I..--.R.--Q.REG-P-Q.-EY--P--.VA.NN.V-...Y..T....N...IL-G---.  322
NP_001039635    274  M..--.WF--Q.REGPQ-A.-..--R---A..NKVI-...Y..T...C....IL-G---.  317
XP_001490227    234  I..--.YTPP.SPR--.--.-DA--P--PA..NR.V-...Y..T...C....IL-A---.  277
CAE69535        243  N..--..F--KTGD----------------..QVYS-...Y.AS.T.T...I.T-.---A  277
NP_499668       242  N..--..-----------.K-TG--D----..QIYS-...Y.AS.T.T...V.T-.---A  276
NP_510509       268  I.L--.KF--GYDR--Q-.----------Y..YASS-...Y..LLY.S...A.Q-T---.  305
AAH97934        42   I..--.R.--Q.REG-P-Q.-EY--P--..A.NN.I-...Y..T........IL-G---.  85
EAW98052        274  I..--.W.--Q.REG-P-WG-DY--P--.V..NK.V-...Y..T...C....IL-G---.  317
CAE63260        236  I.L--.KF--GYDR--Q-.----------Y..YGSS-...Y..LLY.S...A.Q-T---.  273
XP_001378519    423  I..--.KF--H.-R--.-LP-QG--.AP....NEQV-...Y..T...C....IL-G---.  466
AAI26079        8    I..--.R.--Q.REG-P-Q.-EY--P--..A.NN.I-...Y..T........IL-G---.  51
AAH86408        1                                ..A.NN.I-...Y..T........IL-G---.  27
CAE65016        259  ..I--.KF--GY---------DV--N--EV...IV.-..GY..ALI.S..YTIT-.---A  296
NP_497570       259  ..I--.KF--GY---------DV--N--EV...IV.-..GY..VLI.T..YTIT-.---A  296
AAI04716        1                                                    ...IL-G---.  7
XP_645818       273  F.S--.NF--K.-------S-.I--T--N.KSKLTL-..GY.AT.A.L.....L-D---T  311
XP_001520206    262  Y.H--WDF--NLRD--A-HT-RS--A-------R.S-...Y..FLM.L.....L-.---.  299
EDL19769        286  I..--.R.--Q.REG-P-Q.-EY--P--.VA.NN.V-...Y..T........IL-G---.  329
BAC32923        279  I..--.R.--Q.REG-P-Q.-EY--P--.VA.NN.V-...Y..T........IL-G---.  322
EDL10564        231  Y.H--WDF--NIKD--K-YT-LS--K-------R.S-...Y..FLE.L....IL-.---.  268
Q8VCI0          259  Y.H--WDF--NIKD--K-YT-LS--K-------R.S-...Y..FLE.L....IL-.---.  296
BAE42780        160  Y.H--WDF--NIKD--K-YT-LS--K-------R.S-...Y..FLE.L....IL-.---.  197
XP_979751       258  Y.H--WDF--NIKD--K-YT-LS--K-------R.S-...Y..FLE.L....IL-.---.  295
```

```
NP_080082      259  Y.H--WDF--NIKD--K-YT-LS--K-------R.S-...Y..FLE.L....IL-.---.  296
XP_416206      254  Y.H--WNF--NIAD--P-YT-.----------NRVS-...Y..FLV.L....IL-G---.  291
XP_543800      323  Y.H--WDF--NIKD--K-DT-.S---------NR.S-...Y..FLE.L....IL-.---.  360
XP_642833      255  F.V--.N.--KYLF--N-GQ-P--------.ASKV.M..GY..TLS.I.....L-D---T  294
NP_001013949   259  Y.H--WDF--NIKD--K-YT-AS---------NR.S-...Y..FLE.L....IL-.---.  296
XP_001497171   240  Y.HWDFNI--.DKD--.-IS-NR-----------.S-...Y..FLE.L....IL-.---.  277
EDM01610       71   Y.H--WDF--NIKD--K-YT-AS---------NR.S-...Y..FLE.L....IL-.---.  108
XP_001089478   258  Y.H--WDF--NIKD--K-DT-.----------.SR.S-...Y..FLE.L....IL-.---.  295
CAF90435       211  Y.H--WDF--SVAE--P-HA-A.---------GK.S-...Y..FLV.L.....L-G---.  248
AAW25845       5    ..H--.NF--PW-.--I-VD-N.GSQ--K...FAI.-...Y.TYTS.V....IT-.---A  48
CAC13971       235  Y.H--WD----.NI--V-DK-D.--S--SSRLSFSS-Y---..FLE.L.....L-.---.  272
XP_614819      255  Y.H--WD----.NI--V-DK-D.--S--SSRLSFSS-Y---..FLE.L.....L-.---.  292
XP_001157500   211  Y.H--WD----.NI--I-DK-D.--S--SSRLSFSS-Y---..FLE.L....IL-.---.  248
XP_520758      259  Y.H--WD----.NI--I-DK-D.--S--SSRLSFSS-Y---..FLE.L....IL-.---.  296
XP_637940      250  I.S--.NF--..S.--L-VA-AK--S------N.TM-..GY..VLM.V....ML-D---.  288
NP_079105      258  Y.H--WD----.NV--I-DK-D.--S--SSRLSFSS-Y---..FLE.L....IL-.---.  295
Q6P4A8         257  Y.H--WD----.NI--I-DK-D.--S--SSRLSFSS-Y---..FLE.L....IL-.---.  294
BAB15442       211  Y.H--WD----.NI--I-DK-D.--S--SSRLSFSS-Y---..FLE.L....IL-.---.  248
EAW96323       145  Y.H--WD----.NI--I-DK-D.--S--SSRLSFSS-Y---..FLE.L....IL-.---.  182
EAW96322       258  Y.H--WD----.NI--I-DK-D.--S--SSRLSFSS-Y---..FLE.L....IL-.---.  295
XP_698232      241  Y.H--WDF--.LTE--P-HT-A.---------GK.S-...Y..FLV.L.....L-G---.  278
XP_647502      290  Y.F--IN.--Q.N-----.Q-D.--P--M--EYKVS-...S..F.S.K....IT-G---N  328
XP_001370772   145  Y.H--WDF--SIKD--P-MT-KS--S------RHS-...Y..FLE.L....IL-.---.  182
XP_638577      263  F.S--.NQ--Q.SS--D-VS-G.--L-----SKRNI-...Y..ALI.V.....LGD---T  303
XP_803464      266  Y.T--.RV--E-.R--.-----------------V.-M..Y..M.H.I..W.MT-.---E  296
XP_699488      225  Y.H--WN.--K-----------R--K--CNGIRR.S-...Y..TLS.M.....L-G---T  260
XP_642656      252  Y.R--INI--PVAS--.-PY-GS--.--TL--------...Y..LLV.I....Q.-RP--.  289
XP_793919      261  .L---..I--SSYF--K-SQ-AI--L--KLNSPSCQ-LFGIE.FLE.L....IM-.---.  302
AAC31917       116  Y.F--IN.--Q.N-----.Q-D.--P--M--EYKVS-...S..F.S.K....IT-G---N  154
XP_643736      263  F.R--III-------------PD--P--TV..NEIL-.A.YA.VLT.D...FM.P.---T  299
XP_844857      269  Y.T--.AF----------------------G.RFV.-M..Y..LAH.V..W.MT-H---K  299
AAC28456       269  Y.T--.AF----------------------G.RFV.-M..Y..LAH.V..W.MT-H---K  299
XP_844855      269  Y.T--.AF----------------------G.RFV.-M..Y..LAH.V..W.MT-H---K  299
XP_001454659   303  Y.Y--.QF--SGPN--P-YR--------------VS-...K..LLY.K....VLPD---A  337
XP_655293      230  Y..--.TF--P.T.--N-----------AL.---M.-.A.S..FLH.K....IL-K---.  264
XP_650973      233  Y.H--.FF--PWT.--N--------------AQPMS-.A.S..L.H.K.....L-.---N  267
XP_001024340   228  Y.A--.KF--E..---------N--K--DVKASFQA-...R..DLE.K....IL-N---N  265
XP_649424      228  Y.V--.D.----------.Y-GG--R--RL---KMS-...S..L.H.K..Y.TV-.AEDT  265
XP_001453083   199  Y.S--.NF--KL-------K-DK--D--VVNP.-.S-.TAR..DLE.K...FVLWD---T  237

1_15640        159  G----LATMETT--IGNGNTDLY--QYI---TPQ-TN-L-E-FVRNIVANRLATTGKEWS  203
NP_001076460   278  .----.V.L..--...S.AA.W--KFV---Q.R-GSVM-.-WL.........GD.QS.A  323
NP_775813      318  .----.V.L..--...K.PA.W--K.V---R.RGCV-.-.-W..........SD.AT.A  363
EAW98053       318  .----.V.L..--...K.PA.W--K.V---R.RGCV-.-.-W..........SD.AT.A  363
CAG08547       256  .----.V.......--...N.PA.W--KHV---Q.TG.V-F-.-WL.............Q.A  301
BAC28089       323  .----.V.L..--...K.PA.W--K.V---Q..GCV-.-.-WI..V......LD.AT.A  368
BAE37687       323  .----.V.L..--...K.PA.W--K.V---Q..GCE-.-.-WI..V......LD.AT.A  368
```

```
BAE41761       323  .----.V.L....-...K.PA.W--K.V---Q..GCV-.-.-WI..V......LD.AT.A  368
NP_076114      323  .----.V.L....-...K.PA.W--K.V---Q..GCV-.-.-WI..V......LD.AT.A  368
BAC26858       317  .----.V.L....-...K.PA.W--K.V---Q..GCV-.-.-WI..V......LD.AT.A  362
AAH26395       326  .----.V.L....-...K.PA.W--K.V---Q..GCV-.-.-WI..V......LD.AT.A  371
EDM13780       187  .----.V.L....-...K.PA.W--K.V---Q..GCV-.-.-WI.........LD.AT.A  232
BAE41924       323  .----.V.L....-...K.PA.W--K.V---Q..GCV-.-.-WI..V......LD.AT.A  368
AAG44101       323  .----.V.L....-...K.PA.W--K.V---Q..GCV-.-.-WI..V......LD.AT.A  368
Q4QQW8         314  .----.V.L....-...K.PA.W--K.V---Q..GCV-.-.-WI.........LD.AT.A  359
XP_001104551   318  .----.V.L....-...K.PA.W--K.V---R.RGCV-.-.-W..........SD.AT.A  363
XP_509397      318  .----.V.L....-...K.PA.W--K.V---R.RGCV-.-.-W..........SD.AT.A  363
XP_534691      499  .----.V.L....-...R.PA.W--K.V---Q.K-NCV.-.-W...V......LD.DS.A  544
BAB23709       323  .----.V.L....-...K.PA.W--K.V---Q..GCV-.-.-WI..V......LD.AT.A  368
NP_001039635   318  .----.V.L....-...K.PA.W--K.V---Q.TGCV-.-.-WM..V......LD.DS.A  363
XP_001490227   278  .----.V.L....-...R.PA.W--K.V---Q.KHCV-.-.-W..........VD.DS.A  323
CAE69535       278  K----..IL....-...Y.EKSL--DL.---..N-.V-.-T-WI.AEI.H.TSSS.LQ.A  322
NP_499668      277  K----..IL....-...Y.EKSL--DL.---..N-.V-.-T-WI.AEI.H.T.SS.LQ.A  321
NP_510509      306  .-----..VI..---.SVF..S.F--ENT---K.V-GQ-.PT-WI.A..S.Q..RDAR..C  351
AAH97934       86   .----.V.L....-...K.PA.W--K.V---Q..GCV-.-.-WI.........LD.AT.A  131
EAW98052       318  .----.V.L....-...K.PA.W--K.V---R.RGCV-.-.-W..........SD.AT.A  363
CAE63260       274  .-----..VI..---.SVF..S.F--ENT---K.V-GQ-.PT-W..A.IS.Q..RDAR..C  319
XP_001378519   467  R----.V.L....-...Y.A..W--K.V---....-NSV.-.-WL..L......RD.AA.A  512
AAI26079       52   .----.V.L....-...K.PA.W--K.V---Q..GCV-.-.-WI.........LD.AT.A  97
AAH86408       28   .----.V.L....-...K.PA.W--K.V---Q..GCV-.-.-WI.........LD.AT.A  73
CAE65016       297  .----.TSI..---.AIF..S..TDK.M---SAE-GQ-V-HCW..SM.S.L.SK...Q.V  344
NP_497570      297  .----.TSI..---.AIF.QT..TDKFM---K.E-GQ-V-HCWI.SMIS.L.SR...Q.V  344
XP_780319      22          .I....-...S.NE.W--K.V---Q.T-GQI.-.-WA...I.....KNASH.A  64
AAI04716       8    .----.V.L....-...K.PA.W--K.V---Q..GCV-.-.-WI.........LD.AT.A  53
XP_645818      312  K----.VVL..---N.LN.N...--YL.---K.E-SV-.-T-WM.V.I.....NG.QS.C  356
XP_001520206   300  .-----..VLQ.--NNVF..S.L--RHV---V..-AL-.-A-WQ.VR...MV.QG.RD.A  344
EDL19769       330  .----.V.L....-...K.PA.W--K.V---Q..GCV-.-.-WI..V......LD.AT.A  375
BAC32923       323  .----.V.L....-...K.PA.W--K.V---Q..GCV-.-.-WI..V......LD.AT.A  368
EDL10564       269  .----.ILLQ.--NSVY.KT.L--KQV---V.K-.L-.-A-WQ.VR...MM.EG....A  313
Q8VCI0         297  .----.ILLQ.--NSVY.KT.L--KQV---V.K-.L-.-A-WQ.VR...MM.EG....A  341
BAE42780       198  .----.ILLQ.--NSVY.KT.L--KQV---V.K-.L-.-A-WQ.VR...MM.EG....A  242
XP_979751      296  .----.ILLQ.--NSVY.KT.L--KQV---V.K-.L-.-A-WQ.VR...MM.EG....A  340
NP_080082      297  .----.ILLQ.--NSVY.KT.L--KQV---V.K-.L-.-A-WQ.VR...MM.EG....A  341
XP_416206      292  .----.IMLQ.--NSVF.QT.I--KQV---V.E-SL-F-A-WQ.VRI..MM.DS..A.A  336
XP_543800      361  .----.ILLQ.--NSVY.KT.L--KLV---V..-SL-.-A-WQ.VR...MM.ND.RQ.A  405
XP_642833      295  K----IVVI..---N.LM.NN..--HL.---.SE-SV-.-S-WI.V........G.ES.C  339
NP_001013949   297  .----.ILLQ.--NSVY.KT.L--KLV---V.E-SL-.-A-WQ.VR...MM.QG.....  341
XP_001497171   278  .----.ILLQ.--NSVY.KT.L--KLV---V..-SL-.-A-WQ.VR...MM.NG.R..A  322
EDM01610       109  .----.ILLQ.--NSVY.KT.L--KLV---V.E-SL-.-A-WQ.VR...MM.QG.....  153
XP_001089478   296  .----.VLLQ.--NSVY.KT.L--KQ.---I.E-SL-.-A-WQ.VR...MM.DS..R.A  340
CAF90435       249  .----.MMTQ.S--NEIF.SS.F--DSV---..N-SL-F-A-WQ.VRL..S..H..E..A  293
AAW25845       49   N----.TIT..---NNVY.KT.W--EIVRNGSKN-AV-.-T-.M.GM..S...K..E..I  96
CAC13971       273  .----.VLLQ.--NSVY.KT.L--.HV---V..-SL-.-A-WQ.VR..SMM.NN..Q.A  317
XP_614819      293  .----.VLLQ.--NSVY.KT.L--.HV---V..-SL-.-A-WQ.VR..SMM.NN..Q.A  337
```

```
XP_001157500   249  .----.ILLQ..--NSVF.KT.L--KQV---I.E-.L-.-A-WQ.VR...MM.DS..R.A  293
XP_520758      297  .----.ILLQ..--NSVF.KT.L--KQV---I.E-.L-.-A-WQ.VR...MM.DS..R.A  341
XP_637940      289  K----.VVL...NG.KDNDSE.F--KL.---K..-SV-.-T-WI.I..T..I.HS..S.C  335
NP_079105      296  .----.ILLQ..--NSVF.KT.L--KQV---I.E-.L-.-S-WQ.VR...MM.DS..R.A  340
Q6P4A8         295  .----.ILLQ..--NSVF.KT.L--KQV---I.E-.L-.-S-WQ.VR...MM.DS..R.A  339
BAB15442       249  .----.ILLQ..--NSVF.KT.L--KQV---I.E-.L-.-S-WQ.VR...MM.DS..R.A  293
EAW96323       183  .----.ILLQ..--NSVF.KT.L--KQV---I.E-.L-.-S-WQ.VR...MM.DS..R.A  227
EAW96322       296  .----.ILLQ..--NSVF.KT.L--KQV---I.E-.L-.-S-WQ.VR...MM.DS..R.A  340
XP_698232      279  .----.MMTQ..--NNVF.MS.F--SSV---..A-SL-F-A-WQ.VRL..T..R..AQ.A  323
XP_647502      329  K----..I......--NNIY.ES..--..T---I..-SV-.-V-WQ.AMI..MI..NSSD.V  373
XP_001370772   183  .----.VLLQ..--NTVF.IT.L--KQV---I.E-SL-.-A-WQ.VRA..MM.DG..K.A  227
XP_638577      304  R----MVVI....--NSLVTN....--HL.---R.T-.V-.-S-WM.V..S..MS.N....C  348
XP_803464      297  R----..V......--N.IY.DS..--A.L---N.K-.V-S-.-.L.VMI..F...DSPS.V  341
XP_699488      261  .----FFVTQ..--NNVF..S.F--SLV---..K-AL-.-A-WQ.VRA.HS..C...Q.A  305
XP_642656      290  K----.HLT..L--NTIL.QT..--.Q.---NA.-SF-M-Y-W...L......NN.FQ.V  334
XP_793919      303  .----.SMLQ..--NNIF.KT..--K.V---K..-SL-.-A-WQ.VR...MM.RS..D.A  347
AAC31917       155  K----..I......--NNIY.ES..--..T---I..-SV-.-V-WQ.AMI..MI..NSSD.V  199
XP_643736      300  E----MVII....--NDIL..S..--..V---..N-SL-.-Y-...S.I....SN.AQ..T  344
XP_844857      300  R----..V....N-VIHNA.L.R--NHV---GSS-SV-A-T-.L.AMI..FI.VDAPS.V  345
AAC28456       300  R----..V....N-VIHNA.L.R--NHV---GSS-SV-A-T-.L.AMI..FI.VDAPS.V  345
XP_844855      300  R----..V....N-VIHNA.L.R--NHV---GSS-SV-A-T-.L.AMI..FI.VDAPS.V  345
XP_001454659   338  N----MVVI....--NSILDST..--DL.---V.T-.L-.-T-WQ.VPI.HAISNG.EK.T  382
XP_655293      265  SQNH-FSV......--NSIF.NK.FK-.FL---.FK-SV-.-S-WQ.ILGSMYY.PSIQKLL  313
XP_650973      268  AQQKRFGV......--NSFFFKK.N--KL.---..N-SL-.-S-WQ.IL.SMYFGQ.P..IT  316
AAH00909       2                                                   M.DS..R.A  10
XP_001024340   266  .----VVV...S--LN.Y.KSN.--D.L---HYD-SV-P-C-WM.VNL....SSN.S..A  310
XP_649424      266  Q----.FVS....--NSVY.SSV.--ED.---NDK-L--.-S-WQ.ILSGLYYEK.A..FV  309
XP_001181567   2            LQ..--NNIF.KT..--K.V---K..-SL-.-A-WQ.VR...MM.RS..D.A  42
XP_001453083   238  Q----MVVT..S--FN.Y.KEN.--A.F---HYD-SV-P-C-WM.ANI.S...K.PS..M  282

1_15640        204  DLFAEHNSGTYNNQWMVLDYKKF-TP-G---K-----P-L-P-D-G-LLYVLEQIPHY--  246
NP_001076460   324  QT.SRY...........V...N.-V.-.---Q-----A-V-AKQ-.-.FT......GM--  367
NP_775813      364  .I.KRF..........IV...A.-I.-.---G-----.-S-.-G-SRV.TI.....GM--  407
EAW98053       364  .I.KRF..........IV...A.-I.-.---G-----.-S-.-G-SRV.TI.....GM--  407
CAG08547       302  .I.SKY..........IVN.NL.-..-.---.-----TDI-T-N-.-.FV......GL--  345
BAC28089       369  .V.KRF..........IV...A.-L.-N---G-----.-S-.-G-SRV.TI.....GM--  412
BAE37687       369  .V.KRF..........IV...A.-L.-N---G-----.-S-.-G-SRV.TI.....GM--  412
BAE41761       369  .V.KRF..........IV...A.-L.-N---G-----.-S-.-G-SRV.TI.....GM--  412
NP_076114      369  .V.KRF..........IV...A.-L.-N---G-----.-S-.-G-SRV.TI.....GM--  412
BAC26858       363  .V.KRF..........IV...A.-L.-N---G-----.-S-.-G-SRV.TI.....GM--  406
AAH26395       372  .V.KRF..........IV...A.-L.-N---G-----.-S-.-G-SRV.TI.....GM--  415
EDM13780       233  .V.RRF..........IV...A.-I.-N---G-----.-S-.-G-SRV.TI.....GM--  276
BAE41924       369  .V.KRF..........IV...A.-L.-N---G-----.-S-.-G-SRV.TI.....GM--  412
AAG44101       369  .V.KRF......S...IV...A.-L.-N---G-----.-S-.-G-SRV.TI.....GM--  412
Q4QQW8         360  .V.RRF..........IV...A.-I.-N---G-----.-S-.-G-SRV.TI.....GM--  403
XP_001104551   364  .I.KRL..........IV...A.-I.-.---G-----.-S-.-G-SRV.TI.....GM--  407
XP_509397      364  .I.KRF..........IV...A.-I.-.---G-----.-S-.-G-SRV.TI.....GM--  407
```

XP_534691	545	.I.KKF..........IV...A.-V.-.---G-----.-S-.GN-R-V.TI.....GM--	588
BAB23709	369	.V.KRF..........IV...A.-L.-N---G-----.-S-.-G-SRV.TI.....GM--	412
NP_001039635	364	.I.KRF..........IV...A.-V.-.---G-----.-S-.-G-RRV.T......GM--	407
XP_001490227	324	.T.KRF..........IV...A.-V.-.---G-----.-S-.-G-SRV.TI...L.GM--	367
CAE69535	323	EA.GR........E.V.V...Q.-HR-.---.-----.-VQ.-ET.-IIH.V..M.GH--	367
NP_499668	322	EA.GR........E.V.V...Q.-HR-.---.-----E-VQ.-ET.-IIH.V..M.GH--	366
NP_510509	352	K.YSLY.........A.......-K.-N---Q-----.-.-.KN-.-.F.....M.GK--	395
AAH97934	132	.V.RRF..........IV...A.-I.-N---G-----.-S-.-G-SRV.TI.....GM--	175
EAW98052	364	.I.KRF...------------------------------------.-MVV.---------	378
CAE63260	320	K.Y.MY.........A.......-..-N---.-----.-.-A-KY.-.F.....M.GK--	363
XP_001378519	513	...KRF..........IV..NA.-..-.---Q-----A-E-.-TR.-..T......GM--	556
AAI26079	98	.V.RRF..........IV...A.-I.-N---G-----.-S-.-G-SRV.TI.....GM--	141
AAH86408	74	.V.RRF..........IV...A.-I.-N---G-----.-S-.-G-SRV.TI.....GM--	117
CAE65016	345	.T.GRY.........T...W.H.-..-.---Q-----.-I-.-.KD-V.WIS..T.G.--	388
NP_497570	345	.M.GRY.........T...W.Q.-..-E---.-----E-.-.-.KD-V.WIS..T.G.--	388
XP_780319	65	.I...Y..........IV..N..-..-.---Q-----T-P-S-.-.-..V......GK--	107
AAI04716	54	.V.RRF..........IV...A.-I.-N---G-----.-S-.-G-SRV.TI.....GM--	97
XP_645818	357	ET.ERE..........IV..N..-V.-.---V-----K-V-R-.-.-T.F....V.G.--	399
XP_001520206	345	QA.SRC........Y.I..L..V-K.-K---E-----S-.-G-.-.-T.FIV....TL--	387
EDL19769	376	.V.KRF..........IV...A.-L.-N---G-----.-S-.-G-SRV.TI.....GM--	419
BAC32923	369	.V.KRF..........IV...A.-L.-N---G-----.-S-.-G-SRV.TI.....GM--	412
EDL10564	314	QI.SK.........Y....L..V-.I-N---R-----S-.-D-K-.-T..IV....T.--	356
Q8VCI0	342	QI.SK.........Y....L..V-.I-N---R-----S-.-D-K-.-T..IV....T.--	384
BAE42780	243	QI.SK.........Y....L..V-.I-N---R-----S-.-D-K-.-T..IV....T.--	285
XP_979751	341	QI.SK.........Y....L..V-.I-N---R-----S-.-D-K-.-T..IV....T.--	383
NP_080082	342	QI.SK.........Y....L..V-.I-N---R-----S-.-D-K-.-T..IV....T.--	384
XP_416206	337	ET.SKC........Y....L..V-KL-R---.-----S-.-D-.-.-A..IV....TL--	379
XP_543800	406	EI.SS.........Y....L..V-K.-K---Y-----S-.-D-T-.-T..IV....T.--	448
XP_642833	340	QT.SLY.........IIV..N..-IK-.---Y-----G-A-L-.-.-T..I...V.D.--	382
NP_001013949	342	Q..SMY........Y....L..V-.L-K---.-----S-I-D-R-.-A...V....T.--	384
XP_001497171	323	EI.SKY........Y.I..L..V-NV-R---H-----S-.-D-K-.-T..IV....T.--	365
EDM01610	154	Q..SMY........Y....L..V-.L-K---.-----S-I-D-R-.-A...V....T.--	196
XP_001089478	341	.I.SKY........Y....L..V-KL-N---H-----S-.-D-K-.-T..IV....T.--	383
CAF90435	294	RT.SRY........Y....RS.V-RL-.---Y-----S-.-D-.-.-A.T.V....GL--	336
AAW25845	97	TY.KYN..........IF.A.QW--------------.-K-N-K-.-S.LIA..L.GI--	135
CAC13971	318	EV.SKY........Y....L..V-NL-N---H-----S-.-D-E-.-T..IV....T.--	360
XP_614819	338	EV.SKY........Y....L..V-NL-N---H-----S-.-D-E-.-T..IV....T.--	380
XP_001157500	294	.I.SKY........Y....L..V-KL-N---H-----S-.-D-K-.-T..IV....T.--	336
XP_520758	342	.I.SKY........Y....L..V-KL-N---H-----S-.-D-K-.-T..IV....T.--	384
XP_637940	336	EI.EKE..........IV..N..-IK-.---V-----R-V-Q-.-.-T...F..L.G.--	378
NP_079105	341	.I.SKY........Y....L..V-KL-N---H-----S-.-D-K-.-T..IV....T.--	383
Q6P4A8	340	.I.SKY........Y....L..V-KL-N---H-----S-.-D-K-.-T..IV....T.--	382
BAB15442	294	.I.SKY........Y....L..V-KL-N---H-----S-.-D-K-.-T..IV....T.--	336
EAW96323	228	.I.SKY........Y....L..V-KL-N---H-----S-.-D-K-.-T..IA....T.--	270
EAW96322	341	.I.SKY........Y....L..V-KL-N---H-----S-.-D-K-.-T..IA....T.--	383
XP_698232	324	NT.SRY........Y.IV.SS.V-NL-.---V-----S-V-D-.-.-A.T.V....GL--	366
XP_647502	374	KI.S.F.....Q.....F...L.-I.-N---.QQSSSS-.-.-P-N-TFWIA....GQ--	421

```
XP_001370772   228  NI.SRY........Y....L..V-KL-N---E-----S-.-G-N-E-T.FIV....KL--  270
XP_638577      349  EN.QRY..........IVS.NL.-V.-Y---N-----E-.-K-.-.-A........G.--  391
XP_803464      342  KY.SLN............NMAAVAN.-K---T-----.-M-.-A-N-TFW.A..L.GSTY  387
XP_699488      306  QI.SK.......S.Y..V.L.RI-SL-.---R-----Q-I-E-.-W-S.T.V....GL--  348
XP_642656      335  SI.V.N....N.I.FV.....L.-..YS---T-----E-.-Q-S-D-..WIV..Y.GG--  378
XP_793919      348  RIV.RY........Y..I.RT.I-K.-N---V-----A-I-L-.-D-A.W.V..V.TL--  390
AAC31917       200  KI.S.F.....Q.....F...L.-I.-N---.QQSSSS-.-.-P-N-TFWIA....GQ--  247
XP_643736      345  NN.IQY.....S....IV...L.-..-Y---Q-----.-.-Q-P-N-TFWII..L.GG--  387
XP_844857      346  SN.SRES..A........NMGAV-ES-EAMF.-----N-M-A-P-N-TFW....L.GTAP  393
AAC28456       346  SN.SRES..A........NMGAV-ES-EAMF.-----N-M-A-P-N-TFW....L.GTAP  393
XP_844855      346  SN.SRES..A........NMGAV-ES-EAMF.-----N-M-A-P-N-TFW....L.GTAP  393
XP_001454659   383  ST.IK.........YI.I.L.VV-KK-N---T-----.-P-A-K-D-FIWIS.TV.GF--  425
XP_655293      314  QN.EVY.........I.T.LN..-NL-A---G-----H-Q-.-NK.-..MIA....GK--  357
XP_650973      317  KTIGQF..........IINLNSI-EK-N---A-----Q-R-K-K-N-I..IG..M.GK--  359
AAH00909       11   .I.SKY........Y....L..V-KL-N---H-----S-.-D-K-.-T..IV....T.--  53
XP_001024340   311  QV.SNYR...H....I.V..NQW-RA-.---S---------R-K-N-IVWMV.ESFYL--  351
XP_649424      310  E.IKDY.........I.F.VNSW-KK-N---.-----A-E-S------.FIC..M.GL--  349
XP_001181567   43   RIV.RY........Y..I.RT.I-K.-N---V-----A-I-L-.-D-A.W.V..V.TL--  85
XP_979716      1                   ....L..V-.I-N---R-----S-.-D-K-.-T..IV....T.--  28
XP_001453083   283  .I.GQ.R...H....V.T..N.I-FM-.---E--------------------.AFF..--  315

1_15640        247  ---INITDAT-N-VL---RDQSYWPSYNVPSSEFIFNMSG-NQKQ-----VEKFGD----  288
NP_001076460   368  ---.KTA.K.-T-E.---FQTG..A...I.YF.EV..A..-G.EL-----.Q.Y.S----  409
NP_775813      408  ---VVVA.K.-S-E.---YQKT..A...I..F.TV..A..-L.AL-----.AQY..----  449
EAW98053       408  ---VVVA.K.-S-E.---YQKT..A...I..F.TV..A..-L.AL-----.AQY..----  449
CAG08547       346  ---.VS..K.-Q-E.---LQKGF.A...I.YYVD...A..-CNEL-----.....S----  387
BAC28089       413  ---VVVA.K.-A-E.---YKTT..A...I.YF.TV..A..-L.AL-----.AQY..----  454
BAE37687       413  ---VVVA.K.-A-E.---YKTT..A...I.YF.TV..A..-L.AL-----.AQY..----  454
BAE41761       413  ---VVVA.K.-A-E.---YKTT..A...I.YF.TV..A..-L.AL-----.AQY..----  454
NP_076114      413  ---VVVA.K.-A-E.---YKTT..A...I.YF.TV..A..-L.AL-----.AQY..----  454
BAC26858       407  ---VVVA.K.-A-E.---YKTT..A...I.YF.TV..A..-L.AL-----.AQY..----  448
AAH26395       416  ---VVVA.K.-A-E.---YKTT..A...I.YF.TV..A..-L.AL-----.AQY..----  457
EDM13780       277  ---VVVA.K.-A-E.---YKTT..A...I.YF.SV..A..-L.AL-----.AQY..----  318
BAE41924       413  ---VVVA.K.-A-E.---YKTT..A...I.YF.TV..A..-L.AL-----.AQY..----  454
AAG44101       413  ---VVVA.K.-A-E.---YKTT..A...I.YF.TV..A..-L.AL-----.AQY..----  454
Q4QQW8         404  ---VVVA.K.-A-E.---YKTT..A...I.YF.SV..A..-L.AL-----.AQY..----  445
XP_001104551   408  ---VVVA.K.-S-E.---YQKT..A...I..F.TV..A..-L.AL-----.AQY..----  449
XP_509397      408  ---VVVA.K.-S-E.---YQKT..A...I..F.TV..A..-L.AL-----.AQY..----  449
XP_534691      589  ---VVVA.K.-S-E.---YQKT..A...I.YF.SV..A..-L.AL-----.AQY..----  630
BAB23709       413  ---VVVA.K.-A-E.---YKTT..A...I.YF.TV..A..-L.AL-----.AQY..----  454
NP_001039635   408  ---VVVA.R.-S-E.---YQKT..A...I..F.SV..A..-LPAL-----.ARY.P----  449
XP_001490227   368  ---VVVA.K.-S-E.---YQKT..A...I..F.TV..T..-L.AL-----.AQY..----  409
CAE69535       368  ---.VHS.K.-A-H.---FRTT...G..Q.YYKQ.IRL.D-TD.M-----...Y..----  409
NP_499668      367  ---.VHS.K.-A-H.---FRET...G..Q.YYKQ.IRF.D-TD.M-----......----  408
NP_510509      396  ---.VYS.L.-W-FV---EKY..F....I.FFKE.TEI..-FIG.-----AA.M..----  437
AAH97934       176  ---VVVA.K.-A-E.---YKTT..A...I.YF.SV..A..-L.AL-----.AQY..----  217
EAW98052       379  ------A.K.-S-E.---YQKT..A...I..F.TV..A..-L.AL-----.AQY..----  417
```

CAE63260	364	---.VYS.L.-W-FI---EKY..F....I.FFKE.TEI..-FIG.-----AA.L..----	405
XP_001378519	557	---VVVA.R.-E-E.---YEKG..A...L.YF.SV..A..-MPDL-----.K........----	598
AAI26079	142	---VVVA.K.-A-E.---YKTT..A...I.YF.SV..A..-L.AL-----.AQY..----	183
AAH86408	118	---VVVA.K.-A-E.---YKTT..A...I.YF.SV..A..-L.AL-----.AQY..----	159
CAE65016	389	---YESR.M.-W-Y.---KKYT.FA...I.FLPK.SER..-FDTK-----AKQ.A-----	429
NP_497570	389	---YETR.M.-W-Y.---KKYT.FA...I.FLPKVSEI..-FDNK-----ARQ.A-----	429
XP_780319	108	---.HAE.M.-Y-F.---KEH.......T.FFVD.YDK..-WPAE-----KRRY..----	149
AAI04716	98	---VVVA.K.-A-E.---YKTT..A...I.YF.SV..A..-L.AL-----.AQY..----	139
XP_645818	400	---.EFA.V.-.-.-.---.-TG......I.YF.T.......-FNDE-----LTDSS.----	440
XP_001520206	388	---VKFS.Q.-.-.-.---.-AG........FHST.Y.L..-YPDF-----.R...L----	428
EDL19769	420	---VVVA.K.-A-E.---YKTT..A...I.YVALF.HVA	450
BAC32923	413	---VVVA.K.-A-E.---YKTT..A...I.YVALF.HVA	443
EDL10564	357	---VEYS.Q.-.-.-.---.-KG..A...I.FHKT.Y.W..-YPLL-----.H.L.L----	397
Q8VCI0	385	---VEYS.Q.-.-.-.---.-KG..A...I.FHKT.Y.W..-YPLL-----.H.L.L----	425
BAE42780	286	---VEYS.Q.-.-.-.---.-KG..A...I.FHKT.Y.W..-YPLL-----.H.L.L----	326
XP_979751	384	---VEYS.Q.-.-.-.---.-KG..A...I.FHKT.Y.W..-YPLL-----.H.L.L----	424
NP_080082	385	---VEYS.Q.-.-.-.---.-KG..A...I.FHKT.Y.W..-YPLL-----.H.L.L----	425
XP_416206	380	---VEYS.Q.-.-.-.---.-KG......I.FHQK.Y.L..-YASY-------...Y.L----	420
XP_543800	449	---VEYS.Q.-E-.-.---.-KG......I.FH.K.YSW..-YPML-----.Q.L.L----	489
XP_642833	383	---VEYG.Q.-A-I.---.-TG....F.I.FY.N.YGLT.-FNET-----YAQ..N----	423
NP_001013949	385	---VEYS.Q.-.-I.---.-KG..A...I.FHKTVY.W..-YPLL-----.H.L.L----	425
XP_001497171	366	---VEFSEQ.-D-.-.---.-KG......I.FH.K.Y.W..-YPML-----.Q.L.L----	406
EDM01610	197	---VEYS.Q.-.-I.---.-KG..A...I.FHKTVY.W..-YPLL-----.H.L.L----	237
XP_001089478	384	---VEYSEQ.-D-.-.---.-RG......I.FH.K.Y.W..-YPLL-----.Q.L.L----	424
CAF90435	337	---VEHS.Q.-G-A.---.-RG........FHPN.YAL-----.M-----W..H.E----	373
AAW25845	136	---VSSL.V.-K-I.---KING..A...L.FIGD.YTL..-TEEM-----AKM....----	177
CAC13971	361	---VEYSEQ.-A-.-.---.-RG......I.FH.KVY.W..-YPIL-----.K.L.L----	401
XP_614819	381	---VEYSEQ.-A-.-.---.-RG......I.FH.KVY.W..-YPIL-----.K.L.L----	421
XP_001157500	337	---VEYSEQ.-D-.-.---.-KG........FH.K.Y.W..-YPLL-----.Q.L.L----	377
XP_520758	385	---VEYSEQ.-D-.-.---.-KG........FH.K.Y.W..-YPLL-----.Q.L.L----	425
XP_637940	379	---VEYA.V.-.-I.---.-TG....F...YF.T.S.....-FNY.SSSSDSSSSSG----	424
NP_079105	384	---VEYSEQ.-D-.-.---.-KG........FH.K.Y.W..-YPLL-----.Q.L.L----	424
Q6P4A8	383	---VEYSEQ.-D-.-.---.-KG........FH.K.Y.W..-YPLL-----.Q.L.L----	423
BAB15442	337	---VEYSEQ.-D-.-.---.-KG........FH.K.Y.W..-YPLL-----.Q.L.L----	377
EAW96323	271	---VEYSEQ.-D-.-.---.-KG........FH.K.Y.W..-YPLL-----.Q.L.L----	311
EAW96322	384	---VEYSEQ.-D-.-.---.-KG........FH.K.Y.W..-YPLL-----.Q.L.L----	424
XP_698232	367	---VEFS.Q.-Q-T.---.-RG........FHRR.YEL..-YDEM-----WR.Y.E----	407
XP_647502	422	---VKTA.L.-.-I.---NE.G..K...I.YF.S.Y.I..-YSEK-----TDLPPS----	463
XP_001370772	271	---VEFS.Q.-D-I.---.-KG..A...I.FHKTTY.L..-YPML-------...L.L----	311
XP_638577	392	---.EFS.Q.-Q-A.---.-.GW.N...I.FY.T.YDA..Y.NYT-----ANNYS.STIY	437
XP_803464	388	PLGVTAA.M.-E-H.---NKYG..A...I.YF.NVYEI..-YL.K-----EKE....----	432
XP_699488	349	---VVFS.QS-Q-A.---.-.G..A...I.FHSD.YQA..-YGVM-----WK.H.Q----	389
XP_642656	379	---YQAA.V.-L-T.---WE.G......R.YF.EV.DIL.-YPYY-----..........----	420
XP_793919	391	---VASG.Q.-.-I.---.-AG........FY.E.Y.I..-YPEY-----AY.G.A----	431
AAC31917	248	---VKTA.L.-.-I.---NE.G..K...I.YF.S.Y.I..-YSEK-----TDLPPS----	289
XP_643736	388	---FMSA.M.-E-.-.---A-LGN...F.R.FFPE.YDAM.-YSYY-----EKLY..----	428
XP_844857	394	PLG.TSK.M.-S-.-.---NTTG..A...R.YFPNVY.L..-TL.M-----Q.EY..----	438

```
AAC28456       394  PLG.TSK.M.-S-..---NTTG..A...R.YFPNVY.L..-TL.M-----Q.EY..----  438
XP_844855      394  PLG.TSK.M.-S-..---NTTG..A...R.YFPNVY.L..-TL.M-----Q.EY..----  438
XP_001454659   426  ---AVAE.V.AK-.I---SNGN..K...I.FTTEVYDRA.-YTEA-----.K.DPI----  468
XP_655293      358  ---VQ.M.V.-K-L.---LQKG..A...S.F.KK.RDL..-YTLK-----EREY..----  399
XP_650973      360  ---YIVK.V.-P-I.---IN.T..A...I.YIKE..VS..-YL.K-----M.LGKK----  401
AAH00909       54   ---VEYSEQ.-D-..---.-KG........FH.K.Y.W..-YPLL-----.Q.L.L----  94
XP_001024340   352  ---YKPI.V.-Y-Q.---FS.G.VA.....FNQE...ITM-Y.Q----------.Y----  388
XP_649424      350  ---MKSH.V.-E-Y.TSEEK.NM.......FDKE.YSK..-YPT------------.----  388
XP_001181567   86   ---VASG.Q.-.-I.---.-AG........FY.E.Y.I..-YLEY-----AY.G.A----  126
XP_979716      29   ---VEYS.Q.-.-..---.-KG..A...I.FHKT.Y.W..-YPLL-----.H.L.L----  69
XP_001453083   316  ----KVH.L.-.TL.---AS.G.VA.....LDQE..DSL-------------.YPK----  350

1_15640        289  W-FTYDKTPRALIFKRDHGNVQDMDSMIKLMRYN-DYKNDPLS---RC-------N----  332
NP_001076460   410  .-.SFSQN...Q..R.NQTQ.T.IQ..ER.....-N.LE....----..E------E----  454
NP_775813      450  .-.S..GS...Q..R.NQSL.......VR.....-.FLH....---L.-------KA---  494
EAW98053       450  .-.S..GS...Q..R.NQSL.......VR.....-.FLH....---L.-------KA---  494
CAG08547       388  .-.SL.RN...Q....NQTA.T.VE...R.....-NF.E....---.Y-------EG---  432
BAC28089       455  .-.S.T.N...K..Q..QSL.E...A.VR.....-.FLH....---L.-------EA---  499
BAE37687       455  .-.S.T.N...K..Q..QSL.E...A.VR.....-.FLH....---L.-------EA---  499
BAE41761       455  .-.S.T.N...K..Q..QSL.E...A.VR.....-.FLH....---L.-------EA---  499
NP_076114      455  .-.S.T.N...K..Q..QSL.E...A.VR.....-.FLH....---L.-------EA---  499
BAC26858       449  .-.S.T.N...K..Q..QSL.E...A.VR.....-.FLH....---L.-------EA---  493
AAH26395       458  .-.S.T.N...K..Q..QSL.E...A.VR.....-.FLH....---L.-------EA---  502
EDM13780       319  .-.S.TRN...K..Q..QSL.E.V.T.VR.....-.FLH....---L.-------EA---  363
BAE41924       455  .-.S.T.N...K..Q..QSL.E...A.VR.....-.FLH....---L.-------EA---  499
AAG44101       455  .-.S.T.N...K..Q..QSL.E...A.VR.....-.FLH....---L.-------EA---  499
Q4QQW8         446  .-.S.TRN...K..Q..QSL.E.V.T.VR.....-.FLH....---L.-------EA---  490
XP_001104551   450  .-.S..GS...Q..R.NQSL.......VR.....-.FLH....---L.-------KA---  494
XP_509397      450  .-.S..GS...Q..R.NQSL.......VR.....-.FLH....---L.-------KA---  494
XP_534691      631  .-.S..GS...R..R.NQSL.H.L...LR.....-.FLH....---L.-------KA---  675
BAB23709       455  .-.S.T.N...K..Q..QSL.E...A.VR.....-.FLH....---L.-------EA---  499
NP_001039635   450  .-.S..GS...Q..R.N.SL.H.L...MR.....-.FLH....---L.-------KA---  494
XP_001490227   410  .-.S..GS...Q..R.NQSL.R.L....Q.....-.FLH....---L.-------KE---  454
CAE69535       410  .-YS..........A...K..H..E...A...S.-N.TK....---K.-------D----  453
NP_499668      409  .-YS..............NT.T..S...A...S.-N.TK....---K.-------D----  452
NP_510509      438  .-.KWGAS...K..E......H.L..LTA.....-.....EF.---K.-------K----  481
AAH97934       218  .-.S.TRN...K..Q..QSL.E.V.T.VR.....-.FLH....---L.-------EA---  262
EAW98052       418  .-.S..GS...Q..R.NQSL.......VR.....-.FLH....---L.-------KA---  462
CAE63260       406  .-.KWGAS...K..E...IK.H.L.TLTA.....-..Q..EF.---K.-------K----  449
XP_001378519   599  .-.S.K.S...R..E.NQSL.H......R...                              629
AAI26079       184  .-.S.TRN...K..Q..QSL.E.V.T.VR.....-.FLH....---L.-------EA---  228
AAH86408       160  .-.S.TRN...K..Q..QSL.E.V.T.VR.....-.FLH....---L.-------EA---  204
CAE65016       430  .-YEWGGS...R..D...QT.T.I..LT......-N..HEEFA---.-------K----  473
NP_497570      430  .-.DWGGS...R..D...SK.T.I..LT......-..THEEFA---.-------K----  473
XP_780319      150  .-.S.N..A..N..R.NQTE.T.LE.........-N.EH....---A.-------D----  193
AAI04716       140  .-.S.TRN...K..Q..QSL.E.V.T.VR.....-.FLH....---L.-------EA---  184
XP_645818      441  Y-EA.EEDA.SQ..R..ANK.YSLTDFQAI....-NFQ.....---HG-------D----  484
```

XP_001520206	429	D-.S.ELA...K..R..Q.K.T.LE..KFI....-N.QK..Y.---GG-------.----	472
EDL10564	398	D-YS..LA...K..R..Q...T..A..KYI....-N..E..Y.---KG-------D----	441
Q8VCI0	426	D-YS..LA...K..R..Q...T..A..KYI....-N..E..Y.---KG-------D----	469
BAE42780	327	D-YS..LA...K..R..Q...T..A..KYI....-N..E..Y.---KG-------D----	370
XP_979751	425	D-YS..LA...K..R..Q...T..A..KYI....-N..E..Y.---KG-------D----	468
NP_080082	426	D-YS..LA...K..R..Q...T..A..KYI....-N..E..Y.---KG-------D----	469
XP_416206	421	D-.S.ELA...K..R..Q.K.TNLE..KYI....-N.QH..YA---EH-------.----	464
XP_543800	490	E-YS..MAS..K..R..Q.K.I..A..KYI....-N..K..YT---KG-------D----	533
XP_642833	424	.-.S.QAS..SM.....AN.IHSLTQFQAML...-NWQ...F.---QG-------.----	467
NP_001013949	426	D-YS..LA...K..R..Q.T.T..A..KHI....-N..V..Y.---KG-------D----	469
XP_001497171	407	D-.S..LA...K..R..Q.K.T.VE..KYI....-N.EK..Y.---.G-------D----	450
EDM01610	238	D-YS..LA...K..R..Q.T.T..A..KHI....-N..V..Y.---KG-------D----	281
XP_001089478	425	D-YS..LA...K..R..Q.K.T.VA..KYI....-N..K..Y.---KG-------D----	468
CAF90435	374	D-.S..LC...K..R..QAD.E.L..LKHI..F.-...A..Y.---KG-------D----	417
AAW25845	178	.-YVHN..A..K..R...HK.V.FP..LS.....-.FM.......---T.-------P----	221
CAC13971	402	D-YS..LAS..K..R..Q.K.T..E..KYI....-N..Q..Y.---KG-------D----	445
XP_614819	422	D-YS..LAS..K..R..Q.K.T..E..KYI....-N..Q..Y.---KG-------D----	465
XP_001157500	378	D-YS..LA...K..R..Q.K.T..A..KYI....-N..K..Y.---.G-------D----	421
XP_520758	426	D-YS..LA...K..R..Q.K.T..A..KYI....-N..K..Y.---.G-------D----	469
XP_637940	425	S-IA.EQY..SQ..R..SNK.YSISDFQAF.....-.FQ....A---YG-------D----	468
NP_079105	425	D-YS..LA...K..R..Q.K.T.TA..KYI....-N..K..Y.---.G-------D----	468
Q6P4A8	424	D-YS..LA...K..R..Q.K.T.TA..KYI....-N..K..Y.---.G-------D----	467
BAB15442	378	D-YS..LA...K..R..Q.K.T.TA..KYI....-N..K..Y.---.G-------D----	421
EAW96323	312	D-YS..LA...K..R..Q.K.T.TA..KYI....-N..K..Y.---.G-------D----	355
EAW96322	425	D-YS..LA...K..R..Q.K.T.TA..KYI....-N..K..Y.---.G-------D----	468
XP_698232	408	D-.S..LC...K..R..QSA.T..S.LKHI....-E..S..Y.---QG-------.----	451
XP_647502	464	YQYS.E.C..S...S.NASD.LNFED.KS..QF.-N..T....---YS------------	507
XP_001370772	312	D-YS..LA...K..R..Q.Q.T.IK..K.I....-N..K..Y.---.G-------.----	355
XP_638577	438	Y-MS.QTC...E..RNFA.Y.ESLEDFQS.L...-.FEY...------------H----	479
XP_803464	433	Y-.S.TNCS..KM...NESD.VSLE..KR.....-N..E.N..LIQN.TGAGMNDT----	486
XP_699488	390	D-.S..LS...K..R..Q.K.TSLETLKDI.....-..RK..Y.---KK-------H----	433
XP_642656	421	L-...EYN...N..R...SKLETLQD.MNIID..-Q..T..F.---M-------------	462
XP_793919	432	D-IS.QLA...K..R..Q...V..E.FK.I..F.-......Y.---EG-------D----	475
AAC31917	290	YQYS.E.C..S...S.NASD.LNFED.KS..QF.-N..T....	331
XP_643736	429	I-IS..LN..SKM.R..VP..MSL.G.QQI.TQ.-N..S..F.---GG------------	471
XP_844857	439	F-YS.KNYS..R..E..Q.S.V.IE..KR.....-N.TK..F.LIPN.-------TGAVG	489
AAC28456	439	F-YS.KNYS..R..E..Q.S.V.IE..K......-N.TK..F.LIPN.-------TGAVG	489
XP_844855	439	F-YS.KNYS..R..E..Q.S.V.IE..KR.....-N.TK..F.LIPN.-------TGAVG	489
XP_001454659	469	K-YS.EQC..GQMMN..APK.KSL.D.KRFI..-.....AI.---GN-------.----	512
XP_655293	400	R-YS.TRC..RR..D.EGKSLKTINDIKRF.LL.-KF.T...T---EG-------R----	443
XP_650973	402	E-YN.TGSS.....T..GARIHSI.DVK...TM.-H..T..I.---N-------------	443
AAH00909	95	D-YS..LA...K..R..Q.K.T.TA..KYI....-N..K..Y.---.G-------D----	138
XP_001024340	389	G-.N.TND...IE.AQYSK..STTKDVM..I.L.HNATG..------------------	428
XP_649424	389	H-DE.TSCS..K..N..VK...TIED.KHI.L..-..Q..EF.---KN-------D----	432
XP_001181567	127	D-IS.QLA...K..R..Q.K.V..E.FK.I..F	158
XP_979716	70	D-YS..LA...K..R..Q...T..A..KYI....-N..E..Y.---KG-------D----	113
XP_001453083	351	T-TS.NDDV..RQ.RMYQ..LKTVKEVG..V...--------------------K----	384

1_15640	333	----------CTP-PYSA-ENAISARSDL---N----P----PDGTYPFAA----L----	361
NP_001076460	455	----------.D.-HQN.-..S.......---.----.----AN.S...G.----.----	483
NP_775813	495	----------.N.-QPNG-..........---.----.----AN.S...K.----.----	523
EAW98053	495	----------.N.-QPNG-..........---.----.----AN.S...Q.----.----	523
CAG08547	433	----------.D.-.ANG-..........---.----.----AN.....G.----.----	461
BAC28089	500	----------.N.-KPN.-..........---.----.----AN.S...Q.----.----	528
BAE37687	500	----------.N.-KPN.-..........---.----.----AN.S...Q.----.----	528
BAE41761	500	----------.N.-KPN.-..........---.----.----AN.S...Q.----.----	528
NP_076114	500	----------.N.-KPN.-..........---.----.----AN.S...Q.----.----	528
BAC26858	494	----------.N.-KPN.-..........---.----.----AN.S...Q.----.----	522
AAH26395	503	----------.N.-KPN.-..........---.----.----AN.S...Q.----.----	531
EDM13780	364	----------.S.-KPN.-..........---.----.----AN.S...Q.----.----	392
BAE41924	500	----------.N.-KPN.-..........---.----.----AN.S...Q.----.----	528
AAG44101	500	----------.N.-KPN.-..........---.----.----AN.S...Q.----.----	528
Q4QQW8	491	----------.S.-KPN.-..........---.----.----AN.S...Q.----.----	519
XP_001104551	495	----------.D.-QPNG-..........---.----.----AN.S...Q.----.----	523
XP_509397	495	----------.D.-QPNG-..........---.----.----AN.S...Q.----.----	523
XP_534691	676	----------.S.-QAN.-..........---.----.----AN.S...Q.----.----	704
BAB23709	500	----------.N.-KPN.-..........---.----.----AN.S...Q.----.----	528
NP_001039635	495	----------...-KPNG-..........---.----.----AN.S...Q.----.----	523
XP_001490227	455	----------.S.-QPNG-..........---.----.----AN.S...PV----.----	483
CAE69535	454	----------.N.-....-....AC....---.----.----VN.....KS----.----	482
NP_499668	453	----------.N.-....-....AC....---.----.----LN.....KS----.----	481
NP_510509	482	----------.N.-....-.AG....G..---.----.----AN...E.PG----Q----	510
AAH97934	263	----------.S.-KPN.-..........---.----.----AN.S...Q.----.----	291
EAW98052	463	----------.N.-QPNG-..........---.----.----AN.S...Q.----.----	491
CAE63260	450	----------.N.-....-.AG....G..---.----.----AN...E.PG----Q----	478
AAI26079	229	----------.S.-KPN.-..........---.----.----AN.S...Q.----.----	257
AAH86408	205	----------.S.-KPN.-..........---.----.----AN.S...Q.----.----	233
CAE65016	474	----------...D.WT.-.GG....G..---.----I----.G...EVES----M----	503
NP_497570	474	----------...N..TG-.GG....G..---.----T----.G...EVES----M----	503
XP_780319	194	----------.K.-....-....AS....---M----.----AK.R...S.----.----	222
AAI04716	185	----------.S.-KPN.-..........---.----.----AN.S...Q.----.----	213
XP_645818	485	-----------------.-A.Q..S.F..---.----S----..SQ--------------	501
XP_001520206	473	--------------.C.----T.CC.E..---.----L-------.A.VPG---------	491
EDL10564	442	--------------.C.----T.CC.E..---.-----------.AS.SPG---------	460
Q8VCI0	470	--------------.C.----T.CC.E..---.-----------.AS.SPG---------	488
BAE42780	371	--------------.C.----T.CC.E..---.-----------.AS.SPG---------	389
XP_979751	469	--------------.C.----T.CC.E..---.-----------.AS.SPG---------	487
NP_080082	470	--------------.C.----T.CC.E..---.-----------.AS.SPG---------	488
XP_416206	465	----------PC--------.T.CC.E..---.----.-------SF.VP.---------	483
XP_543800	534	----------PC--------.T.CC.E..---.-----------------S----.----	547
XP_642833	468	-----------------.-G.Q..S.F..VTAD----D----.NNQ.--------.----	489
NP_001013949	470	--------------.C.----T.CC.E..---.EASPS----.G.C.-------------	491
XP_001497171	451	----------PC--------.T.CC.E..---.----S----QNPS--------------	467
EDM01610	282	--------------.C.----T.CC.E..---.EASPS----.G.C.-------------	303

```
XP_001089478  469  ----------PC--------.T.CC.E..---.----S----.NPS--------------  485
CAF90435      418  ----------PCK-SICC-RGDLR.ETP.-------------.S.C.-------------  439
AAW25845      222  ----------.K.-..TS-NS.....DE.---.----D----.K.Q..IPS----W----  250
CAC13971      446  ----------PC--------.TVCC.E..---.SHSPS----.G.C.-------------  467
XP_614819     466  ----------PC--------.TVCC.E..---.SHSPS----.G.C.-------------  487
XP_001157500  422  ----------PC--------.T.CC.E..---.----S----.NPS--------------  438
XP_520758     470  ----------PC--------.T.CC.E..---.----S----.NPS--------------  486
XP_637940     469  -----------------P-G.Q..S.F..---I----T----.QNN--------------  485
NP_079105     469  ----------PC--------.T.CC.E..---.----S----.NPS--------------  485
Q6P4A8        468  ----------PC--------.T.CC.E..---.----S----.NPS--------------  484
BAB15442      422  ----------PC--------.T.CC.E..---.----S----.NPS--------------  438
EAW96323      356  ----------PC--------.T.CC.E..---.----S----.NPS--------------  372
EAW96322      469  ----------PC--------.T.CC.E..---.----S----.NPS--------------  485
XP_698232     452  ----------PC--------KS.CC.N..--------------------------.----  463
XP_647502     508  ----------------.P-L.S..S.G..---L----T----IENGNRS.V---------  530
XP_001370772  356  ----------PC--------.T.CC.E..---.----SKRPV.A.C.              377
XP_638577     480  ----------KL.-F.-----..AS.Y..---S----K----KNPS-..-----------  500
XP_803464     487  ----------.N.-.F..-MLT.AS.G..---.----.----AGDAKNYGPLYEY.----  519
XP_699488     434  ----------PCK-SICC-R.DLRL.RP---------H----.G.C.-------------  455
XP_642656     463  --------------G.PG--.S.N..F.IKGGS----L----.S.NPIYSW----F----  490
XP_793919     476  -----------------P-SKS.CM.G..MT-S----.M---.N.C.-------------  496
XP_643736     472  ----------------FP-G...A..Y..G--G----G----.AEPLSWSF----I----  496
XP_844857     490  MDDDGNVTNV.K.-....-MLS.A..G..---.----.----.GNATEYGP----.VRSV  532
AAC28456      490  MDDDGNVTNV.K.-....-MLS.A..G..---.----.----.GNATEYGP----.VRSV  532
XP_844855     490  MDDDGNVTNV.K.-....-MLS.A..G..---.----.----.GNATEYGP----.VRSV  532
XP_001454659  513  -----------------P-G....S.L..LET.----.----------------------  528
XP_655293     444  -----------------P-GAT.A..Y..---D----E----SRPS-..G.----V     465
XP_650973     444  ----------------KP-R.Q.A..Y..---EI---D----S.YKF..-----------  465
AAH00909      139  ----------PC--------.T.CC.E..---.----S----.NPS--------------  155
XP_001024340  429  -------------------SD..AP.F..--------------------LN----Q----  441
XP_649424     433  -----------------P-KES.AS.Y..RV-D----R----.N----------------  449
XP_979716     114  --------------.C.----T.CC.E..---.-----------.AS.SPG---------  132
XP_001453083  385  ----------V.A-EFD.CDG.L.P.C..---G-----------NG.T.-----------  408

1_15640       362  GHRAH--GSTDMKL-TN-SSLFANLE-FT-A--VGGPTXGPGASVPMEHVGTSGPTRR  411
NP_001076460  484  QQ.P.--.G....M-.S-..M.RQW.-LL-.--AS..S                      513
NP_775813     524  RQ.S.--.GI.V.V-.S-M..ARI.S-LL-.--AS...                      553
EAW98053      524  RQ.S.--.GI.V.V-.S-M..ARI.S-LL-.--AS...                      553
CAG08547      462  RQ.Q.--.G.....-.S-YQM.RDYA-MI-.--.S...                      491
BAC28089      529  HQ...--.GI.V.V-.S-FT.AKYMS-ML-.--AS...                      558
BAE37687      529  HQ...--.GI.V.V-.S-FT.AKYMS-ML-.--AS...                      558
BAE41761      529  HQ...--.GI.V.V-.S-FT.AKYMS-ML-.--AS...                      558
NP_076114     529  HQ...--.GI.V.V-.S-FT.AKYMS-ML-.--AS...                      558
BAC26858      523  HQ...--.GI.V.V-.S-FT.AKYMS-ML-.--AS...                      552
AAH26395      532  HQ...--.GI.V.V-.S-FT.AKYMS-ML-.--AS...                      561
EDM13780      393  RQ...--.GI.V.V-.S-VA.AKYMS-ML-.--AS...                      422
BAE41924      529  HQ...--.GI.V.V-.S-FT.AKYMS-ML-T--AS...                      558
```

AAG44101	529	HQ...--.GI.V.V-.S-FT.AKYMS-ML-.--AS...	558
Q4QQW8	520	RQ...--.GI.V.V-.S-VA.AKYMS-ML-.--AS...	549
XP_001104551	524	RQ.S.--.GI.V.V	535
XP_509397	524	RQ.S.--.GI.V.V	535
XP_534691	705	HQ.S.--.GI.V.M-.S-MA.AKAFH-II-.--.S...	734
BAB23709	529	HQ...--.GI.V.V-.S-FT.AKYMS-ML-.--AS...	558
NP_001039635	524	HQ.S.--.GI.V.V-.S-TA.AKA.R-LL-.--.S...	553
XP_001490227	484	RQ.S.--.GI.V.V	495
CAE69535	483	.F.D.--.AI.V.V-..-.K.IQD.Q-..-.--.S..PG.VTKD..IFDWR..SL.DK	532
NP_499668	482	.F.D.--.AI.V.V-..-.K.INS.Q-..-.--.S..PG.VTKD..I	520
NP_510509	511	..VN.--.AL.Y.G-..-VE.MKK.Q-.V-.--Q....W.	542
AAH97934	292	RQ...--.GI.V.V-.S-VA.AKYMS-ML-.--AS...	321
EAW98052	492	RQ.S.--.GI.V.V-.S-M..ARI.S-LL-.--AS...	521
CAE63260	479	..VN.--.AL.Y.G-..-VE.MKK.Q-.V-.--Q....W.	510
AAI26079	258	RQ...--.GI.V.V-.S-VA.AKYMS-ML-.--AS...	287
AAH86408	234	RQ...--.GI.V.V-.S-VA.AKYMS-ML-.--AS...	263
CAE65016	504	.F.D.--AGL.F.G-..-YEM.KK.R-.R-.--W...PY.	535
NP_497570	504	.F.D.--AGL.F.G-..-YEM.KKMR-.R-.--W...PYD.	536
XP_780319	223	...L.--.A....V-.T-..MVKS.S-MV-.--.C...	252
AAI04716	214	RQ...--.GI.V.V-.S-VA.AKYMS-ML-.--AS...	243
XP_645818	502	DYD.F--.GV.S.V-.S-F..VNQ.L-VI-.--QS...	531
XP_001520206	492	-------.CY.T.V-SD-MA.A.R.T-AH-.--IS...VQG.LP.	523
EDL10564	461	-------.CY.T.V-AD-IF.ASQYK-AY-.--IS...VQD.	489
Q8VCI0	489	-------.CY.T.V-AD-IF.ASQYK-AY-.--IS...VQD.	517
BAE42780	390	-------.CY.T.V-AD-IF.ASQYK-AY-.--IS...VQD.	418
XP_979751	488	-------.CY.T.V-AD-IF.ASQYK-AY-.--IS...VQD.	516
NP_080082	489	-------.CY.T.V-AD-IF.ASQYK-AY-.--IS...VQD.	517
XP_416206	484	-------.CY.S.V-SD-FR.ASAFT-A.-.--IN..PVQG.LP.	515
XP_543800	548	NPSPG--.CY.T.V-AD-IY.ASEYT-AY-.--IS...TQG.LP.	584
XP_642833	490	DPD.F--.GI.S.V-VS-ADMV.A.L-VN-.--QS..S	519
NP_001013949	492	----------.T.V-AD-IF.ASQYK-AY-.--IS...VQN.	517
XP_001497171	468	---PG--.CY.T.V-AD-IY.ASKYT-AY-.--IS...VQG.LP.	501
EDM01610	304	----------.T.V-AD-IF.ASQYK-AY-.--IS...VQN.	329
XP_001089478	486	---PG--.CY.T.V-AD-IY.ASQYT-SY-.--IS...VQG.LP.	519
CAF90435	440	----------.T.V-.D-FLMAGKFR-AE-.--IN...TQS.	465
AAW25845	251	SY.L.--.G..A.I-VD-L.MINQ.N-MI-.--IS...	280
CAC13971	468	----------.T.V-AD-IY.ASKYK-AY-.--IS...VQG.LP.	496
XP_614819	488	----------.T.V-AD-IY.ASKYK-AY-.--IS...VQG.LP.	516
XP_001157500	439	---PG--.CY.T.V-AD-IY.ASQYT-SY-.--IS...VQG.LP.	472
XP_520758	487	---PG--.CY.T.V-AD-IY.ASQYT-SY-.--IS...VQG.LP.	520
XP_637940	486	-AS.A--.GI.S.V-.S-LE.INQFL-MI-.--QS...	514
NP_079105	486	---PG--.CY.T.V-AD-IY.ASQYT-SY-.--IS...VQG.LP.	519
Q6P4A8	485	---PG--.CY.T.V-AD-IY.ASQYT-SY-.--IS...VQG.LP.	518
BAB15442	439	---PG--.CY.T.V-AD-IY.ASQYT-SY-.--IS...VQG.LP.	472
EAW96323	373	---PG--.CY.T.V-AD-IY.ASQYT-SY-.--IS...VQG.LP.	406
EAW96322	486	---PG--.CY.T.V-AD-IY.ASQYT-SY-.--IS...VQG.LP.	519
XP_698232	464	E.G.SPG.CY.T.V-.D-VR.ARSFM-AE-.--IN...TAG.	499

XP_647502	531	---.F--.GV.S.I-.S-FNQVLT.S-C.-.--IS..STNG.TLP.	565
XP_638577	501	-------.A..T.V-.C-N.MIDQNT-IV-.--IS...TSN.QPI	532
XP_803464	520	.R.D.--.A..A.I-AT-W.	535
XP_699488	456	----------.T.V-AD-YRMAQMFT-AE-.--.N...SQN.	481
XP_642656	491	Y.GT.--.GI.G.A-I.-YDMVNSFT-AV-.--RN...	520
XP_793919	497	----------.T.V-..-LAMA.KQT-SF-V--IN...R.D.SLP.FKW.A	531
XP_643736	497	.--L.--.AI.S.I-.S-Y..LQQNQ-AI-.--IN.M.VT.	527
XP_844857	533	..VNS--.AI.A.IA.W-TGMVK.P.SY.-.HV.C...	566
AAC28456	533	..VNS--.AI.A.IA.W-TGMVK.P.SY.-.HV.C...	566
XP_844855	533	..VNS--.AI.A.IA.W-TGMVK.P.SY.-.HV.C...	566
XP_001454659	529	--K..--..I.G.I-ASLA.VPQ.IA-YI-Q---S...	556
XP_650973	466	-------.AV.C.I-GA-A..--KYK-TL-.--YC...HEG.	492
AAH00909	156	---PG--.CY.T.V-AD-IY.ASQYT-SY-.--IS...VQG.LP.	189
XP_001024340	442	.AYTY--.AI.A.V-..-DK.LQV.Q-SF-M--ISS..	471
XP_649424	450	---.F--.AI.A.I-VS-T.---.PH-L.Y.--IS...	474
XP_979716	133	-------.CY.T.V-AD-IF.ASQYK-AY-.--IS...VQD.	161
XP_001453083	409	-------.GI.G.V-IS-QDMIK.KK-VH-L--ISS.S	433
