# Supplementary material for: Global comparative analysis of ESTs from the southern cattle tick, Rhipicephalus (Boophilus) microplus
Source: BMC Genomics. 2007 Oct 12;8:368. doi: 10.1186/1471-2164-8-368 (PMC2100071; doi:10.1186/1471-2164-8-368)
Supplement: Additional file 1 — Phylogenetic tree of TC14523. The data provided shows the entire tree generated from the TC14523 alignment [file 1471-2164-8-368-S1.pdf]

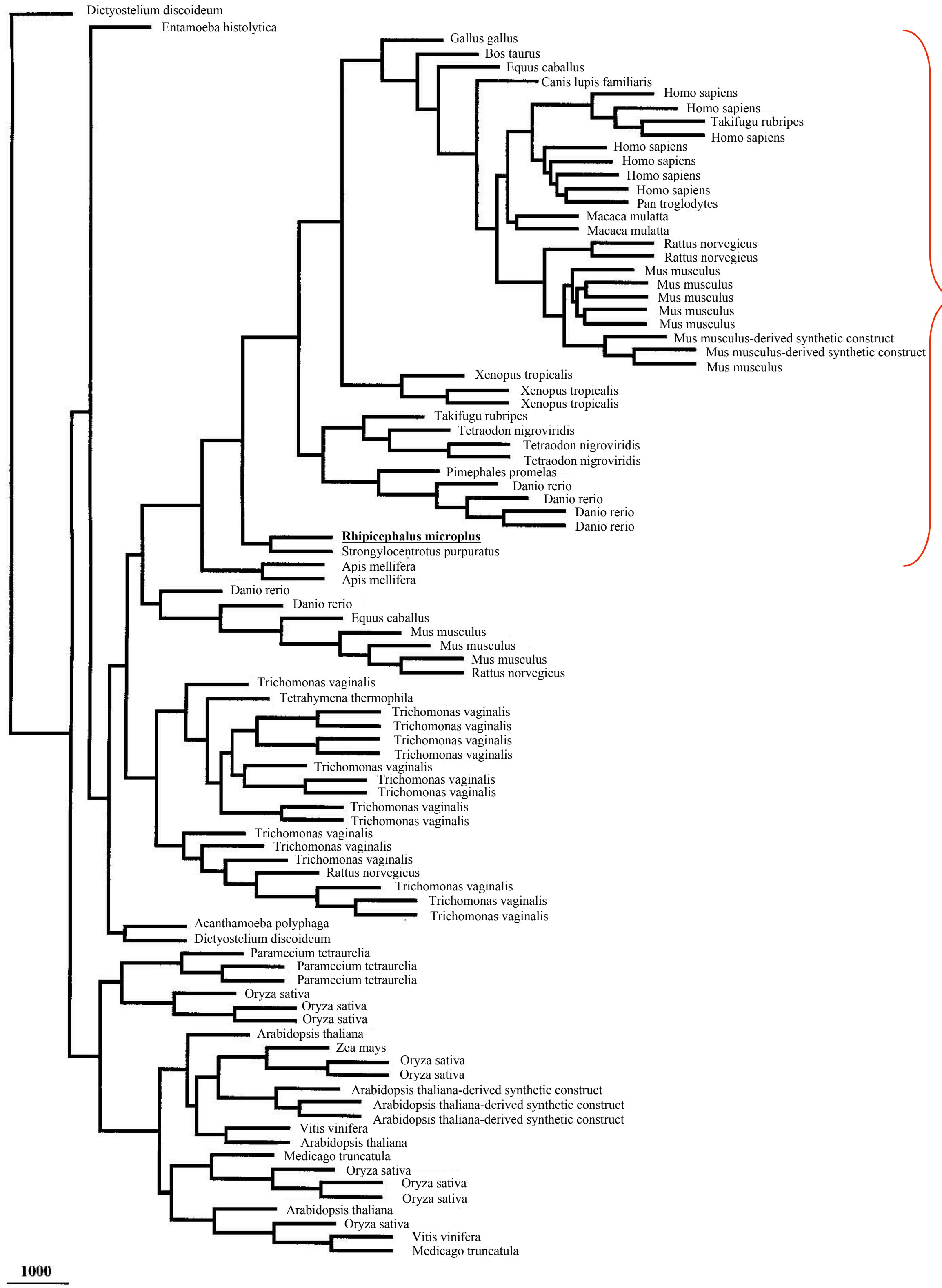

Dictyostelium discoideum
Entamoeba histolytica
Gallus gallus
Bos taurus
Equus caballus
Canis lupis familiaris
Homo sapiens
Homo sapiens
Takifugu rubripes
Homo sapiens
Homo sapiens
Homo sapiens
Homo sapiens
Homo sapiens
Pan troglodytes
Macaca mulatta
Macaca mulatta
Rattus norvegicus
Rattus norvegicus
Mus musculus
Mus musculus
Mus musculus
Mus musculus
Mus musculus
Mus musculus-derived synthetic construct
Mus musculus-derived synthetic construct
Mus musculus
Xenopus tropicalis
Xenopus tropicalis
Xenopus tropicalis
Takifugu rubripes
Tetraodon nigroviridis
Tetraodon nigroviridis
Tetraodon nigroviridis
Pimephales promelas
Danio rerio
Danio rerio
Danio rerio
Danio rerio
Rhipicephalus microplus
Strongylocentrotus purpuratus
Apis mellifera
Apis mellifera
Danio rerio
Danio rerio
Equus caballus
Mus musculus
Mus musculus
Mus musculus
Rattus norvegicus
Trichomonas vaginalis
Tetrahymena thermophila
Trichomonas vaginalis
Trichomonas vaginalis
Trichomonas vaginalis
Trichomonas vaginalis
Trichomonas vaginalis
Trichomonas vaginalis
Trichomonas vaginalis
Trichomonas vaginalis
Trichomonas vaginalis
Trichomonas vaginalis
Trichomonas vaginalis
Trichomonas vaginalis
Rattus norvegicus
Trichomonas vaginalis
Trichomonas vaginalis
Trichomonas vaginalis
Acanthamoeba polyphaga
Dictyostelium discoideum
Paramecium tetraurelia
Paramecium tetraurelia
Paramecium tetraurelia
Oryza sativa
Oryza sativa
Oryza sativa
Arabidopsis thaliana
Zea mays
Oryza sativa
Oryza sativa
Arabidopsis thaliana-derived synthetic construct
Arabidopsis thaliana-derived synthetic construct
Arabidopsis thaliana-derived synthetic construct
Vitis vinifera
Arabidopsis thaliana
Medicago truncatula
Oryza sativa
Oryza sativa
Oryza sativa
Arabidopsis thaliana
Oryza sativa
Vitis vinifera
Medicago truncatula
1000
